# General differential Hebbian learning: Capturing temporal relations between events in neural networks and the brain

**DOI:** 10.1371/journal.pcbi.1006227

**Published:** 2018-08-28

**Authors:** Stefano Zappacosta, Francesco Mannella, Marco Mirolli, Gianluca Baldassarre

**Affiliations:** Laboratory of Computational Embodied Neuroscience, Institute of Cognitive Sciences and Technologies, National Research Council of Italy (LOCEN-ISTC-CNR), Roma, Italy; University College London, UNITED KINGDOM

## Abstract

Learning in biologically relevant neural-network models usually relies on Hebb learning rules. The typical implementations of these rules change the synaptic strength on the basis of the co-occurrence of the neural events taking place at a certain time in the pre- and post-synaptic neurons. Differential Hebbian learning (DHL) rules, instead, are able to update the synapse by taking into account the temporal relation, captured with derivatives, between the neural events happening in the recent past. The few DHL rules proposed so far can update the synaptic weights only in few ways: this is a limitation for the study of dynamical neurons and neural-network models. Moreover, empirical evidence on brain spike-timing-dependent plasticity (STDP) shows that different neurons express a surprisingly rich repertoire of different learning processes going far beyond existing DHL rules. This opens up a second problem of how capturing such processes with DHL rules. Here we propose a general DHL (G-DHL) rule generating the existing rules and many others. The rule has a high expressiveness as it combines in different ways the pre- and post-synaptic neuron signals and derivatives. The rule flexibility is shown by applying it to various signals of artificial neurons and by fitting several different STDP experimental data sets. To these purposes, we propose techniques to pre-process the neural signals and capture the temporal relations between the neural events of interest. We also propose a procedure to automatically identify the rule components and parameters that best fit different STDP data sets, and show how the identified components might be used to heuristically guide the search of the biophysical mechanisms underlying STDP. Overall, the results show that the G-DHL rule represents a useful means to study time-sensitive learning processes in both artificial neural networks and brain.

## Author summary

Which learning rules can be used to capture the temporal relations between activation events involving pairs of neurons in artificial neural networks? Previous computational research proposed various differential Hebbian learning (DHL) rules that rely on the activation of neurons and time derivatives of their activations to capture specific temporal relations between neural events. However, empirical research of brain plasticity, in particular plasticity depending on sequences of pairs of spikes involving the pre- and the post-synaptic neurons, i.e., spike-timing-dependent plasticity (STDP), shows that the brain uses a surprisingly wide variety of different learning mechanisms that cannot be captured by the DHL rules proposed so far. Here we propose a general differential Hebbian learning (G-DHL) rule able to generate all existing DHL rules and many others. We show various examples of how the rule can be used to update the synapse in many different ways based on the temporal relation between neural events in pairs of artificial neurons. Moreover, we show the flexibility of the G-DHL rule by applying it to successfully fit several different STDP processes recorded in the brain. Overall, the G-DHL rule represents a new tool for conducting research on learning processes that depend on the timing of signal events.

## Introduction

Most learning rules used in bio-inspired or bio-constrained neural-network models of brain derive from Hebb’s idea [1, 2] for which “cells that fire together, wire together” [3]. The core of the mathematical implementations of this idea is *multiplication*. This captures the correlation between the pre- and post-synaptic neuron activation independently of the timing of their firing.

Time is however very important for brain processing and its learning processes [4]. *Differential Hebbian learning* (DHL) rules [5, 6] are learning rules that change the synapse in different ways depending on the specific timing of the events involving the pre- and post-synaptic neurons. For example, the synapse might tend to increase if the pre-synaptic neuron activates *before* the post-synaptic neuron, and decrease if it activates *after* it. As suggested by their name, DHL rules use *derivatives* to detect the temporal relations between neural events. Here we will use the term *event* to refer to a relatively short portion of a signal that first monotonically increases and then monotonically decreases. Events might for example involve the activation of a firing-rate unit in an artificial neural network, or the membrane potential of a real neuron, or a neurotransmitter concentration change. DHL rules use the positive part of the first derivative of signals to detect the *initial part* of events, and its negative part to detect their *final part*. By suitably multiplying the positive/negative parts of the derivative of events related to different signals, DHL rules can modify the synapse in different ways depending on how their initial/final parts overlap in time.

To the best of our knowledge, current DHL rules are basically two: one proposed by Kosko [5] and one proposed by Porr, Wörgötter and colleagues [6, 7]. These rules modify the synapse in specific ways based on the temporal relation between the pre- and post-synaptic events. Formulating other ways to modify synapses based on event timing is the first open problem that we face here.

The development of dynamical neural-network models and learning mechanisms that, as DHL, are able to take time into consideration is very important. Indeed, the brain is an exquisitely dynamical machine processing the continuous flow of information from sensors and issuing a continuous flow of commands to actuators so its understanding needs such types of models [8–11]. In this respect, neuroscientific research on *spike timing dependent plasticity* (STDP; [12]) clearly shows how synaptic changes strongly depend on the temporal relation between the spikes of the pre- and post-synaptic neurons. Given the typical shape of spikes, an important class of STDP models, called *phenomenological models* [13], abstracts over the features of the spike signals and directly links the synaptic strengthening, Δ*w*, to the time interval separating the pre-synaptic and post-synaptic spikes, Δ*t*, on the basis of a function of the type Δ*w* = *f*(Δ*t*) [12, 14]. Such a function is usually designed by hand and reflects the synaptic changes observed in experimental data. [15]. The function *f*(Δ*t*) generates a typical *learning kernel* that when plotted shows a curve where each Δ*t* causes a certain Δ*w*. Phenomenological models are simple but are applicable only to spike events. In comparison, DHL rules are more complex but have the advantage of computing the synaptic update as the step-by-step interaction (based on multiplication) between the pre-synaptic and post-synaptic events. Therefore they are applicable to any complex signal that might exhibit events with variable time courses.

When applied to the study of STDP, the property of DHL rules just mentioned also opens up the interesting possibility of using them to investigate the actual biophysical neural events following and caused by the spikes that actually lead to the synaptic change, as first done in [16]. The chain of processes changing the synapse is also captured by *biophysical models* (e.g., see [14, 17]). These models can capture those processes in much biological detail (mimicking specific neurons, neuromodulators, receptors, etc.) but at the cost of being tied to specific phenomena. Because the level of abstraction of DHL rules lies between that of phenomenological models and that of biophysical models, DHL represents an important additional research tool.

Experimental study of STDP [18, 19] shows that different types of neurons, for example excitatory/inhibitory neurons in different parts of the brain, implement a surprisingly rich repertoire of learning kernels. It is reasonable to assume that the brain employs such learning mechanisms to implement different computational functions. In this respect, an interesting fourth class of models appropriate for studying STDP, which might be called *functional models*, aims to derive, or to justify, specific STDP learning kernels based on normative computational principles [20–23].

Investigating the functions of different STDP kernels is not in the scope of this work. However, assuming that the variety of learning kernels discovered through STDP experiments supports different *functions* relevant to neural processing and that analogous functions might be needed in artificial neural networks, it is important to understand the computational *mechanisms* that might generate such a variety of learning kernels. In this respect, an important question is this: is there a DHL learning rule, or a set of them, that can generate the complete variety of learning kernels found in the brain? Some existing research shows how different STDP learning kernels can arise from the same biophysical mechanisms [17], or from the same DHL-based model [24]. However, these studies propose specific mechanisms to address a sub-set of STDP data sets rather than proposing a general way to systematically reproduce STDP learning kernels. Understanding the extent to which DHL can capture the known STDP phenomena, and how this can be done, is thus a second important open problem that we address here.

The rest of the paper addresses the two open problems indicated above in the following ways. As a first contribution of the paper, the Section ‘G-DHL and the systematisation of DHL’ considers the first open problem—how different DHL rules can be generated in a systematic fashion—by proposing a general framework to produce DHL rules. In particular, the section first reviews the DHL rules proposed so far in the literature; then it presents the G-DHL rule and shows how it is able to generate the existing DHL rules and many others; and finally it shows how one can filter the neural signals to generate events that correspond to the features of interest and can use memory traces to apply the G-DHL rule to events separated by time gaps.

As a second contribution of the paper, the Section ‘Using G-DHL to fit STDP data sets’ deals with the second open problem—understanding if and how G-DHL can be used to capture known STDP phenomena. To this end, the section first illustrates how the G-DHL synapse update caused by a pre- and post-synaptic spike pair can be computing analytically rather than numerically, and then it presents a collection of computational tools to automatically search the rule components and parameters to fit a given STDP data set.

Addressing the same second open problem, and as a third contribution of the paper, the Section ‘Using G-DHL to fit STDP data sets’ uses those computational tools to show how the G-DHL rule is able to reproduce several learning kernels from the STDP literature. To this end, the section first uses G-DHL to fit the classic STDP data set of Bi and Poo [25]; then it illustrates how the G-DHL components found by the fitting procedure can be heuristically useful to search for the biophysical mechanisms underlying a given STDP data set; and finally it shows how to apply the G-DHL rule to systematically capture different aspects of all the STDP data sets reviewed by Caporale and Dan [18] (such as their temporal span, long-term potentiation/depression, and variability around zero inter-spike intervals—e.g. sharp depression-potentiation passages, non-learning plateaus, Hebbian/anti-Hebbian learning).

The Section ‘Discussion’ closes the paper by analysing the main features of G-DHL and its possible development. All software used for this research is available for download from internet (https://github.com/GOAL-Robots/CNR_140618_GDHL).

## Methods

### G-DHL and the systematisation of DHL

#### Existing differential Hebbian learning rules

Since DHL rules have been contrasted to the Hebb rule [5], we start by presenting the continuous-time formulation of it:

$$\begin{array}{c} \dot{w}=\left(1/\tau \right)\cdot u_{2}\cdot u_{1}, \end{array}$$ (1)

where *u*_1_ and *u*_2_ are respectively the pre- and post-synaptic neuron activations, and $\dot{w}$ is the instantaneous change of the connection weight. Since this Hebb rule captures the pre- and post-synaptic neuron activation co-occurrence, it is ‘symmetric in time’: the more two neurons activate distantly in time, the lower the synaptic update, independently of the temporal order of their activation. Indeed, even in this dynamical formulation the Hebb rule is not a DHL rule (see Section 1.1 in S1 Supporting Information).

In a relevant work, Kosco [5] highlighted some key elements of Differential Hebbian Learning (DHL), also introducing this name. First, he shifted attention from correlation to causality and as a consequence stressed the importance of considering that a “cause temporally precedes its effect”. Second, he proposed that to capture causality one should focus on *concomitant variations* rather than on *concomitant activations* as in the Hebb rule. He thus proposed a learning rule leading to “impute causality” when the activations of the two neurons change in the same direction, and to impute “negative causality” when they move in opposite directions, based on the *first derivative* of the activation of the neurons:

$$\begin{array}{c} \dot{w}=\left(1/\tau \right)\cdot {\dot{u}}_{2}\cdot {\dot{u}}_{1}. \end{array}$$ (2)

Kosko learning rule indeed implies strengthening of the synapse when the pre- and post-synaptic neurons activate at the same time, as their activations increase/decrease at the same time, and to weaken it when their activations do not fully overlap in time (see Section 1.1 in S1 Supporting Information). However, the rule’s learning kernel is symmetric in time as it does not discriminate the sign of the temporal difference between the events.

The timing of events is a central element of most quantitative definitions of causality (e.g., in Granger causality, a popular statistical approach to capture ‘causality’ [26]). The importance of the temporal ordering of neural events was articulated by Porr, Wörgötter and colleagues [6, 7] who proposed the learning rule:

$$\begin{array}{c} \dot{w}=\left(1/\tau \right)\cdot {\dot{u}}_{2}\cdot u_{1}. \end{array}$$ (3)

This rule leads to an *asymmetric* learning kernel (see Section 1.1 in S1 Supporting Information). Indeed, when the activation *u*_1_ of the pre-synaptic neuron has a transient increase *before* an increase in the activation *u*_2_ of the post-synaptic neuron, then *u*_1_ mainly overlaps with the positive portion of the derivative ${\dot{u}}_{2}$, rather than with its following negative part, so the weight is enhanced. Conversely, if *u*_1_ has a transient increase *after* a transient increase of *u*_2_, then *u*_1_ mainly overlaps with the negative portion of the derivative ${\dot{u}}_{2}$, so the weight is depressed. This mechanism based on the derivative works only if the activations of the neurons exhibit a smooth increase followed by a smooth decrease (‘event’). In the case of a sharp activation (e.g., a neuron spike), such smoothness can be obtained by filtering the signals before applying the rule, for example with a low-pass filter. This filtering indeed formed an integral part of the original proposal of the rule [6]. For higher clarity and control, however, here we will separate the core of DHL rules from the filters possibly applied to the signals before the rules.

As discussed in [7], Porr-Wörgötter rule has a close relation with learning rules previously proposed within the reinforcement learning literature [27, 28]. Indeed, to our knowledge Barto and Sutton [29] were the first to propose various learning rules that might be now considered DHL rules (although not yet called and studied as such), for example to model how in classical conditioning experiments animals learn to anticipate an unconditioned stimulus *y* on the basis of a cue *x*: $\Delta w=\dot{y}\cdot \bar{x}$ (where $\bar{x}$ is a decaying memory trace).

The different behaviour of the three rules presented above can be best understood by considering their learning kernels. As mentioned in the introduction, learning kernels can be directly expressed as mathematical relations involving the time separating the events, Δ*t*, and the resulting synaptic update, Δ*w*. For example phenomenological models of STDP often use an exponential function to express such a relation [13, 14]:

$$\begin{array}{c} \Delta w=\{\begin{array}{cc} e^{-\frac{\Delta t}{\tau }} & \text{if}\;\Delta t>0\\ -e^{\frac{\Delta t}{\tau }} & \text{if}\;\Delta t\leq 0. \end{array} \end{array}$$ (4)

**Fig 1:**
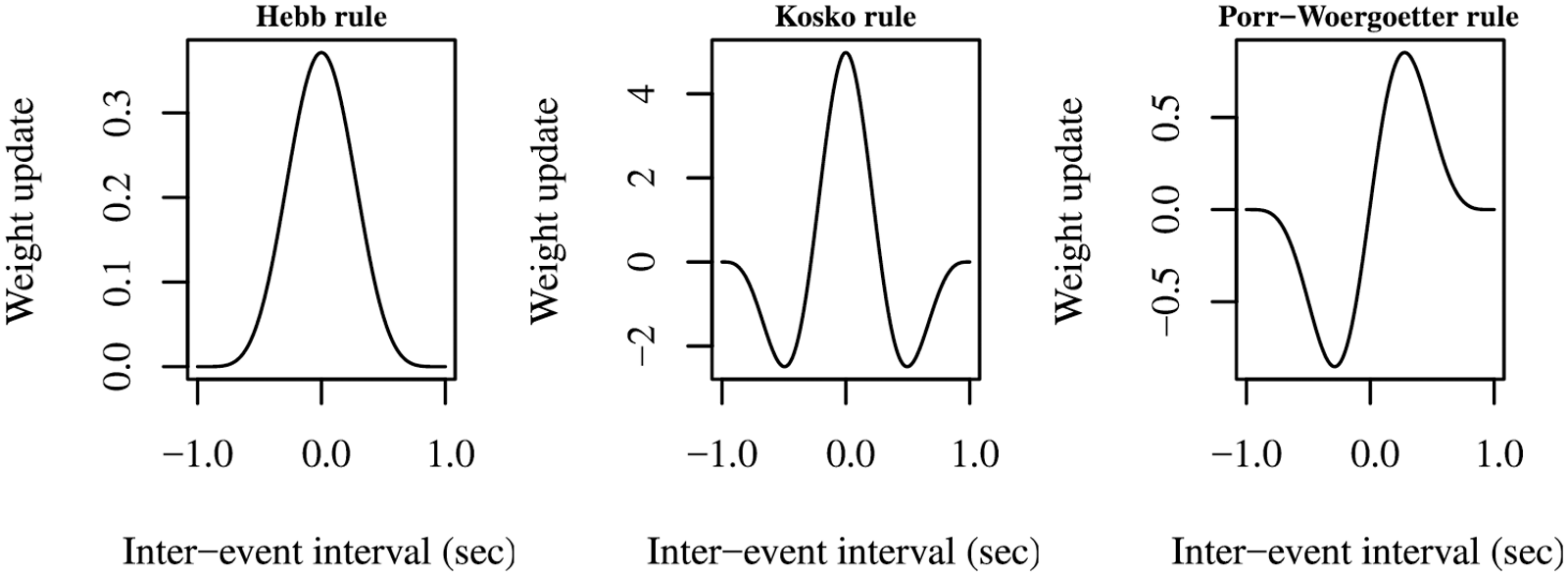
Learning kernels produced by the rules of Hebb, Kosco, and Porr-Wörgötter. Each graph has been plotted by computing the connection weight update resulting from different Δ*t* inter-event delays ranging in [−1.0, 1.0]. Events were represented by a cosine function ranging over (−*π*, +*π*) and suitably scaled and shifted (see Section 1.1 in S1 Supporting Information for details).

In the context of DHL, learning kernels can be computed by integrating (summing) over time the multiple instantaneous weight changes caused by the learning rule:

$$\begin{array}{c} \Delta w=\int_{-\infty }^{+\infty }\left(1/\tau \right)\;\dot{w}\left(u_{1}\left(t\right),u_{2}\left(t\right)\right)\;dt, \end{array}$$ (5)

where $\dot{w}\left(\cdot ,\;\cdot \right)$ is the function giving the instantaneous weight change produced by the learning rule, as given, for example, by Eqs 1, 2 or 3. Fig 1 shows the learning kernels of the Hebb, Kosko, and Porr-Wörgötter learning rules obtained with events based on a cosine function. Only the Porr-Wörgötter rule causes a positive synapse update for Δ*t* > 0 and a negative one for Δ*t* < 0.

The Porr-Wörgötter learning rule was the first DHL rule used to model empirical data on STDP [16]. In this respect, its learning kernel resembles the kernel observed in the most studied form of STDP [25]. This resemblance was also used to formulate hypotheses about the biophysical mechanisms underlying the target STDP data [16], an interesting idea also followed here.

#### The systematisation of Hebb rules

Given the large number and heterogeneity of Hebb rules, Gerstner and Kistler [30] proposed a way to systematise many of them into one composite formula. We now briefly describe the approach they used because the formulation of the G-DHL rule shares some analogies with it.

The rule proposed by Gerstner and Kistler combines the possible *multiplications* between the power functions of degree 0, 1, and 2 of the activations of the pre- and post-synaptic neurons. The elements multiplied are therefore $\left\{u_{1}^{0},u_{1}^{1},u_{1}^{2}\right\}$ for the pre-synaptic neuron and $\left\{u_{2}^{0},u_{2}^{1},u_{2}^{2}\right\}$ for the post-synaptic neurons (the subscript indexes, ‘1’ and ‘2’, respectively refer to the pre-/post-synaptic neurons; the superscript indexes indicate powers). The proposed rule was then:

$$\begin{array}{ccc} \Delta w & = & \alpha_{0,0}+\\ & & \alpha_{1,0}\cdot u_{1}+\alpha_{0,1}\cdot u_{2}+\\ & & \alpha_{1,1}\cdot u_{1}\cdot u_{2}+\alpha_{2,0}\cdot u_{1}^{2}+\alpha_{0,2}\cdot u_{2}^{2} \end{array}$$ (6)

Multiplications involving higher-degree powers, and other elements of the sum, might be needed to include other Hebb rules. For example, a power 4 is needed to represent an interesting Hebb rule implementing independent component analysis [31]: Δ**w** = **u**_1_ · *u*_2_^3^ − **w**.

#### General differential Hebbian learning (G-DHL) rule

While the Gerstner-Kistler’s systematisation relies on *power functions* of neuron activations, the systematisation of G-DHL relies on the positive and negative parts of the *derivatives* of such activations. To show this, we first give a more accurate definition of the events on the basis of which such derivatives are computed. As mentioned, an event is intended here as a portion of the signal, lasting for a relatively short time, featuring a monotonically increasing value followed by a monotonically decreasing value. The Section ‘From neural signals to events’ discusses how G-DHL can be applied to any signal, for example directly to the neural signals, thus responding to events embedded in them, or to pre-filtered signals, thus responding to events generated by the filters.

Events are important for G-DHL because it uses the increasing part and the decreasing part of the pre- and post-synaptic events to capture, through the derivatives, their temporal relation. Indeed, the increasing part of an event marks its *starting portion* whereas its decreasing part marks its following *ending portion* (see Section 1.2 in S1 Supporting Information). The time overlap between these portions of the events allows G-DHL to detect their temporal relation, as we now explain in detail.

G-DHL detects the increasing part of an event in the neural signal *u*_*i*_ on the basis of the positive part of the first derivative ${\dot{u}}_{i}$, namely with ${\left[{\dot{u}}_{i}\right]}^{+}$ (where [⋅]^+^ is the *positive-part function* for which [*u*_*i*_]^+^ = 0 if *u*_*i*_ < 0 and [*u*_*i*_]^+^ = *u*_*i*_ if *u*_*i*_ ≥ 0). G-DHL detects the decreasing part of the event on the basis of the absolute value of the negative part of the first derivative of the signal, namely with ${\left[{\dot{u}}_{i}\right]}^{-}$ (where [⋅]^−^ is the *negative-part function* for which [*u*_*i*_] = 0 if *u*_*i*_ > 0 and [*u*_*i*_]^−^ = −*u*_*i*_ if *u*_*i*_ ≤ 0). Assuming the neural signals *u*_*i*_ are positive (if they are not, they can be suitably pre-processed to this purpose), and since the functions [⋅]^+^ and [⋅]^−^ always return positive or null values, we can assume G-DHL always works on positive or null values.

The basic G-DHL rule studied here is formed by combining through multiplication the pre-synaptic elements up to the first order derivative, $\left\{u_{1},{\left[{\dot{u}}_{1}\right]}^{+},{\left[{\dot{u}}_{1}\right]}^{-}\right\}$, with the post-synaptic elements up to the first order derivative, $\left\{u_{2},{\left[{\dot{u}}_{2}\right]}^{+},{\left[{\dot{u}}_{2}\right]}^{-}\right\}$. This generates 3 × 3 = 9 possible combinations (but one combination is not used, as explained below) that are then summed. The G-DHL formula changing the synapse between two neurons is then:

$$\begin{array}{ccc} \dot{w} & = & \sigma_{p,p}\cdot {\left[{\dot{u}}_{1}\right]}^{+}\cdot {\left[{\dot{u}}_{2}\right]}^{+}+\sigma_{p,n}\cdot {\left[{\dot{u}}_{1}\right]}^{+}\cdot {\left[{\dot{u}}_{2}\right]}^{-}+\\ & & \sigma_{n,p}\cdot {\left[{\dot{u}}_{1}\right]}^{-}\cdot {\left[{\dot{u}}_{2}\right]}^{+}+\sigma_{n,n}\cdot {\left[{\dot{u}}_{1}\right]}^{-}\cdot {\left[{\dot{u}}_{2}\right]}^{-}+\\ & & \eta_{s,p}\cdot u_{1}\cdot {\left[{\dot{u}}_{2}\right]}^{+}+\eta_{s,n}\cdot u_{1}\cdot {\left[{\dot{u}}_{2}\right]}^{-}+\\ & & \eta_{p,s}\cdot {\left[{\dot{u}}_{1}\right]}^{+}\cdot u_{2}+\eta_{n,s}\cdot {\left[{\dot{u}}_{1}\right]}^{-}\cdot u_{2}, \end{array}$$ (7)

where *σ* and *η* are coefficients, $\dot{w}$ is the instantaneous change of the synapse, *u*_1_ and *u*_2_ are the activations of respectively the pre- and post-synaptic neurons, ${\dot{u}}_{1}$ and ${\dot{u}}_{2}$ are their derivatives, and the subscript indexes {*s*, *p*, *n*} refer respectively to the neuron activation *u*_*i*_, its derivative positive part ${\left[{\dot{u}}_{i}\right]}^{+}$, and its derivative negative part ${\left[{\dot{u}}_{i}\right]}^{-}$.

The *σ* and *η* coefficients are very important as: (a) they establish, with their positive/negative sign, if the components to which they are associated either depress or enhance the synapse: in a biological context they establish if the component causes a ‘long term potentiation’—LTP—or a ‘long term depression’—LTD (see the Section ‘Results’); (b) they assign a weight to the contribution of each component to the overall synaptic change. On this basis, the coefficients allow the generation of many different learning kernels. The fact that the rule is based on a linear combination of kernels also facilitates its application. In particular, it facilitates setting its parameters manually or through automatic search procedures.

G-DHL is formed by eight components: four components involving derivative×derivative multiplications and coefficients *σ*, henceforth called *differential components*; and four components involving signal×derivative multiplications and coefficients *η*, henceforth called *mixed components*. The signal×signal combination is not considered as it gives rise to the ‘non-differential’ Hebb rule that is already obtained by two differential components, namely the ‘positive derivative×positive derivative’ component and the ‘negative derivative×negative derivative’ component. In general, any DHL rule based on the multiplication between two events that are derived in the same way from the pre- and post-synaptic signals leads to a symmetric Hebb rule that maximally changes the synapse when the two events coincide in time.

**Fig 2:**
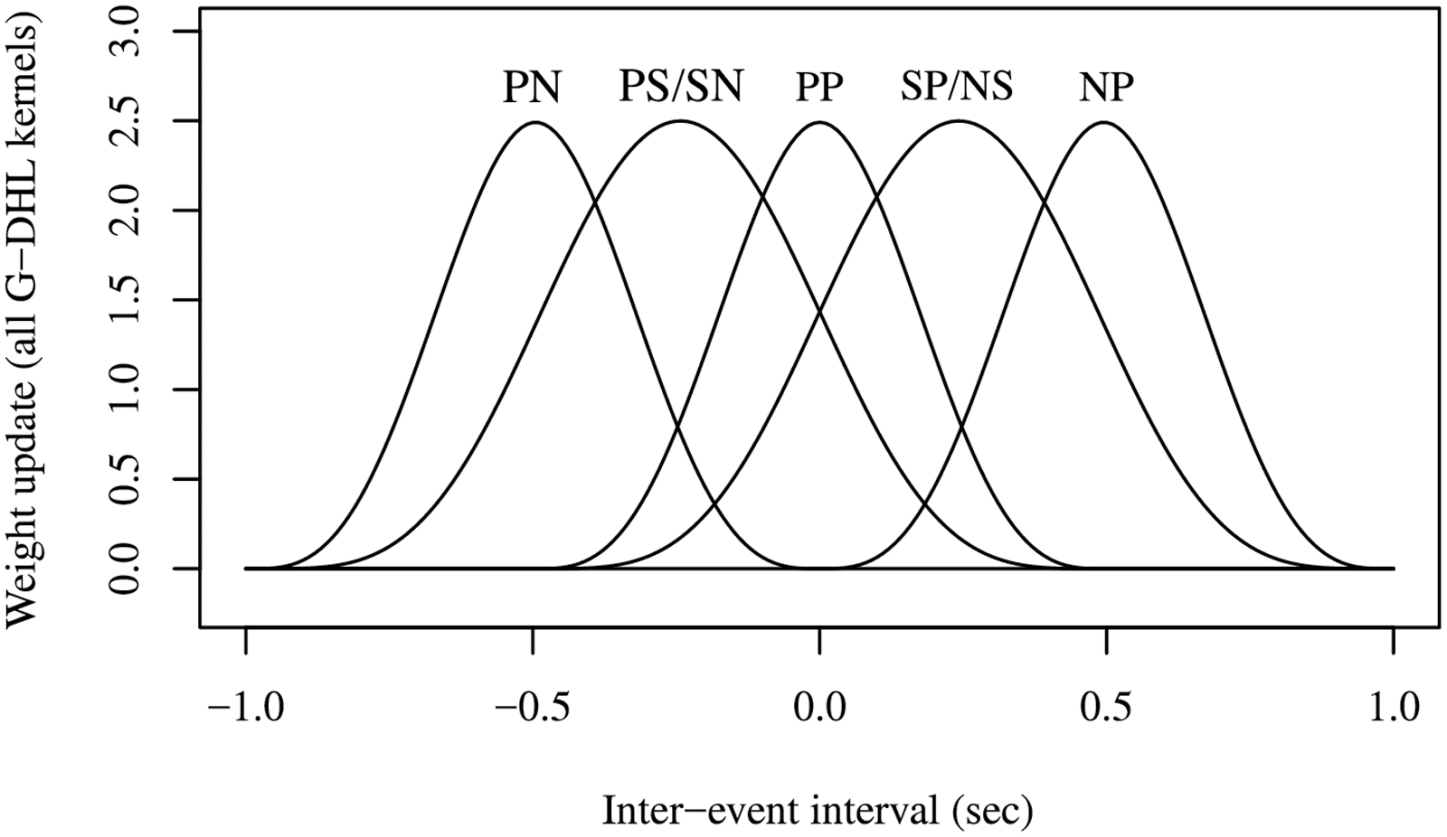
Superposition of learning kernels of the G-DHL rule components. The learning kernels considered correspond to different inter-event intervals, with events represented by a cosine function as in Fig 1. The kernels are indicated with pairs of letters referring respectively to the pre- and post-synaptic neuron, where ‘S’ refers to [*u*_*i*_], ‘P’ to ${\left[{\dot{u}}_{i}\right]}^{+}$, and ‘N’ to ${\left[{\dot{u}}_{i}\right]}^{-}$. PS/SN kernels overlap, and so do SP/NS kernels.

Fig 2 shows the learning kernels of the G-DHL components. Some components overlap because here we considered symmetric events (cosine functions). Section 1.3 in S1 Supporting Information shows the learning kernels of the different G-DHL components resulting from both symmetric and asymmetric events: in the asymmetric case the eight kernels do not overlap.

Analogously to the combination of exponential terms of the neural activations in [30], the G-DHL rule could be extended by considering derivatives beyond the first order, i.e., by multiplying the pre-synaptic elements $\left\{u_{1},{\left[{\dot{u}}_{1}\right]}^{+},{\left[{\dot{u}}_{1}\right]}^{-},{\left[{\ddot{u}}_{1}\right]}^{+},{\left[{\ddot{u}}_{1}\right]}^{-},...\right\}$ with the post-synaptic elements $\left\{u_{2},{\left[{\dot{u}}_{2}\right]}^{+},{\left[{\dot{u}}_{2}\right]}^{-},{\left[{\ddot{u}}_{2}\right]}^{+},{\left[{\ddot{u}}_{2}\right]}^{-},...\right\}$ [32, 33]. Here we focus only on DHL involving first-order derivatives: the study of DHL rules involving higher-order derivatives might be carried out in the future.

#### The G-DHL captures different DHL rules

Fig 2 illustrates that the G-DHL kernels cover the time intervals around the critical value of zero in a regular fashion. This implies that the linear combination of the kernels implemented by the rule through the *σ* and *η* coefficients can be very expressive, i.e., it is able to capture several different possible temporal relations between the pre- and post-synaptic events (hence the name *General* DHL—G-DHL). Linearly combining kernels is commonly used in machine learning to approximate target functions, for example, in radial-basis-function neural networks and support vector machines [34, 35]. The number of G-DHL kernels is small compared to the number used in common machine learning algorithms, but as we shall see it is rich enough to incorporate existing DHL rules and to model a large set of STDP phenomena.

Note that although this relationship to kernel methods is relevant, it is also important to consider that the kernels of the G-DHL rule are not directly designed to capture the ‘time-delay/weight-update’ mapping of a specific STDP dataset, as it would happen in machine learning kernel-based regression methods. Rather, the G-DHL kernels are generated by the step-by-step interaction of different combinations of the pre-/post-synaptic events and their derivative positive/negative parts. Thus, the fact that the resulting kernel profiles form a set of basis functions covering the inter-event interval in a regular fashion is a rather surprising and welcome result. As shown in Section 1.3 in S1 Supporting Information, if the events are asymmetric then none of the eight kernels overlap and they form an even more dense set of regularly distributed basis functions.

The DHL rules proposed in the literature are special cases of the G-DHL rule. As a first case, we consider the Kosko learning rule [5] (Eq 2). G-DHL generates this rule with the following parameter values:

$$\begin{array}{c} \sigma_{p,p}=1,\;\sigma_{p,n}=-1,\sigma_{n,p}=-1,\;\sigma_{n,n}=1,\\ \eta_{s,p}=0,\;\eta_{s,n}=0,\;\eta_{p,s}=0,\;\eta_{n,s}=0,\; \end{array}$$ (8)

for which the G-DHL rule becomes:

$$\begin{array}{l} \dot{w}={\left[{\dot{u}}_{1}\right]}^{+}\cdot {\left[{\dot{u}}_{2}\right]}^{+}-{\left[{\dot{u}}_{1}\right]}^{+}\cdot {\left[{\dot{u}}_{2}\right]}^{-}-{\left[{\dot{u}}_{1}\right]}^{-}\cdot {\left[{\dot{u}}_{2}\right]}^{+}+{\left[{\dot{u}}_{1}\right]}^{-}\cdot {\left[{\dot{u}}_{2}\right]}^{-}=\\ {\;[{\dot{u}}_{1}]}^{+}\cdot \left({\left[{\dot{u}}_{2}\right]}^{+}-{\left[{\dot{u}}_{2}\right]}^{-}\right)+\left(-{\left[{\dot{u}}_{1}\right]}^{-}\right)\cdot \left({\left[{\dot{u}}_{2}\right]}^{+}-{\left[{\dot{u}}_{2}\right]}^{-}\right)={\dot{u}}_{2}\cdot {\dot{u}}_{1}, \end{array}$$ (9)

where ${\dot{u}}_{2}\cdot {\dot{u}}_{1}$ is Kosco DHL rule.

As a second case, we consider the Porr-Wörgötter rule [6] (Eq 3). The G-DHL generates this rule using the following coefficients:

$$\begin{array}{c} \sigma_{p,p}=0,\;\sigma_{p,n}=0,\;\sigma_{n,p}=0,\;\sigma_{n,n}=0\\ \eta_{s,p}=\lambda ,\;\eta_{s,n}=-\lambda ,\;\eta_{p,s}=0,\;\eta_{n,s}=0, \end{array}$$ (10)

where λ is a positive parameter. With these parameters the G-DHL rule becomes:

$$\begin{array}{l} \dot{w}=\lambda \cdot u_{1}\cdot {\left[{\dot{u}}_{2}\right]}^{+}-\lambda \cdot u_{1}\cdot {\left[{\dot{u}}_{2}\right]}^{-}=\\ \lambda \cdot u_{1}\cdot \left({\left[{\dot{u}}_{2}\right]}^{+}-{\left[{\dot{u}}_{2}\right]}^{-}\right)=\lambda \cdot {\dot{u}}_{2}\cdot u_{1}, \end{array}$$ (11)

where ${\dot{u}}_{2}\cdot u_{1}$ is the Porr-Wörgötter DHL rule.

**Fig 3:**
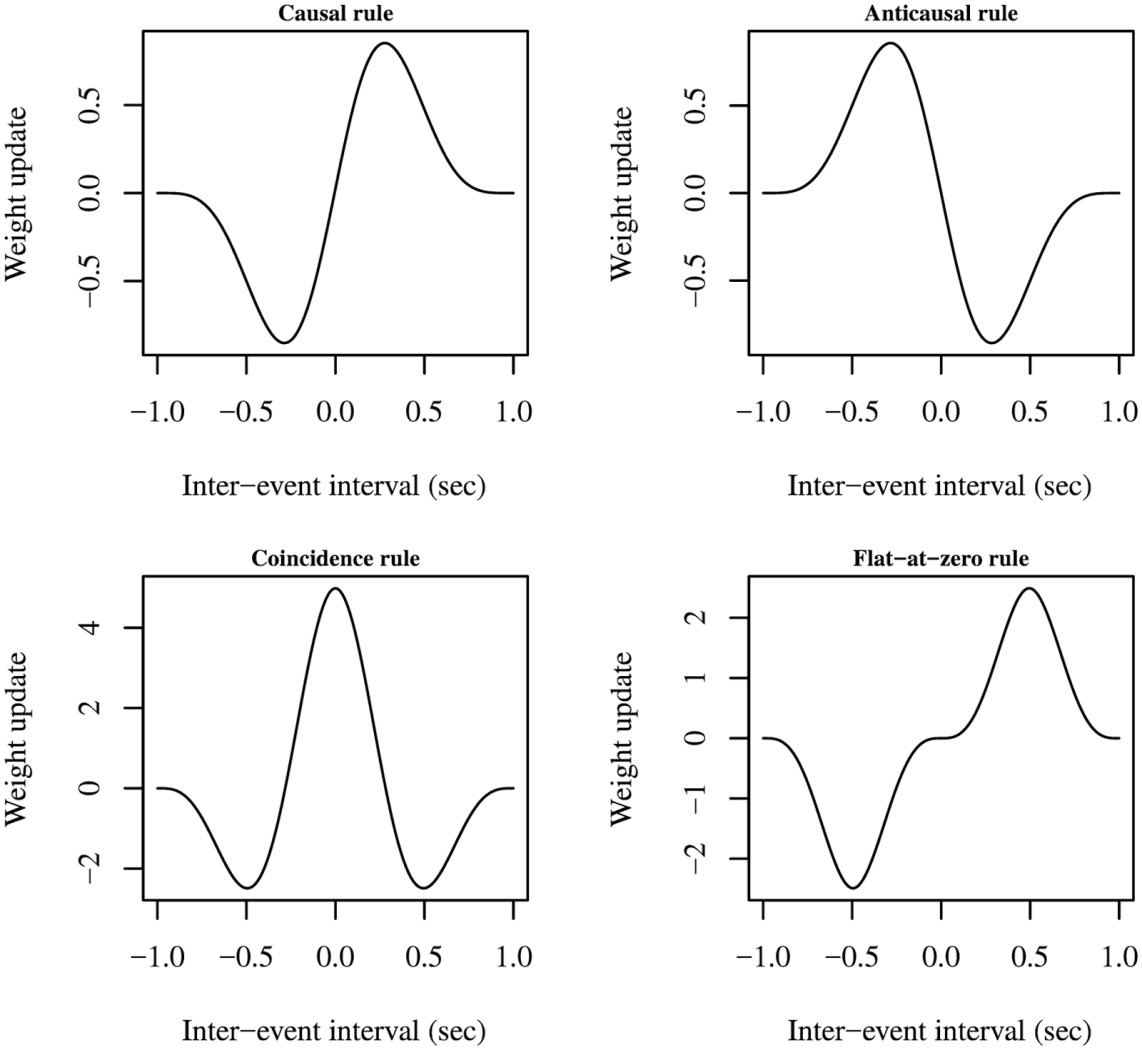
Examples of learning kernels generated by the G-DHL rule. The signals involved events generated with a cosine function (as in Fig 1). In the examples, the G-DHL coefficients were set as follows (the rule names are arbitrary): Causal rule: *σ*_*p*,*p*_ = *σ*_*p*,*n*_ = *σ*_*n*,*p*_ = *σ*_*n*,*n*_ = *η*_*p*,*s*_ = *η*_*n*,*s*_ = 0, *η*_*s*,*p*_ = 1, *η*_*s*,*n*_ = −1. Anticausal rule: *σ*_*p*,*p*_ = *σ*_*p*,*n*_ = *σ*_*n*,*p*_ = *σ*_*n*,*n*_ = *η*_*s*,*p*_ = *η*_*p*,*s*_ = 0, *η*_*s*,*n*_ = 1, *η*_*n*,*s*_ = −1. Coincidence rule: *η*_*s*,*p*_ = *η*_*s*,*n*_ = *η*_*p*,*s*_ = *η*_*n*,*s*_ = 0, *σ*_*p*,*p*_ = *σ*_*n*,*n*_ = 1, *σ*_*p*,*n*_ = *σ*_*n*,*p*_ = −1. Flat-at-zero rule: *σ*_*p*,*p*_ = *σ*_*n*,*n*_ = *η*_*s*,*p*_ = *η*_*s*,*n*_ = *η*_*p*,*s*_ = *η*_*n*,*s*_ = 0, *σ*_*p*,*n*_ = −1, *σ*_*n*,*p*_ = 1.

The G-DHL rule can generate many other possible DHL rules. As an example, Fig 3 shows how different combinations of the G-DHL components can generate a ‘causal’ rule (similar to the Porr-Wörgötter rule), a truly ‘anticausal’ rule (using Kosko’s expression), a ‘coincidence-detection’ rule (similar to the Kosko rule), and a causal rule not changing the synapse for intervals around zero (called here ‘flat-at-zero causal rule’). These examples were not chosen arbitrarily: the Section ‘Results’ will show that each of these rules models one class of STDP processes found in the brain.

#### From neural signals to events

We have seen that G-DHL operates on neural events defined as relatively short portions of a signal that first monotonically increases and then monotonically decreases. This aspect of the G-DHL requires some specifications. First, the fact that the G-DHL operates on events might seem to restrict its applicability. This is not the case because [36]: (a) signals can carry information only if they *change*; (b) events can be generated *from any type of signal change*, as we show here.

Second, there are different possible signal changes that an artificial neural network or the brain might need to process: which changes are relevant depends on the specific filters applied to the signals before they enter the G-DHL rule. For example, the models proposed in [6] and [37] use bandpass/resonator filters. Many other filters could be used to detect different changes [36]. In the brain, these filters might be implemented by the multitude of electro-chemical processes responding in cascade to neuron activation and operating up-stream with respect to other processes implementing the DHL synaptic update (see [38, 39] for some reviews).

Clearly distinguishing between the information processing done by filters, which associate events to the features of interest of the neural signals, and the effects of G-DHL, which modify the synapse on the basis of the temporal relation between those events, is important for best understanding G-DHL. It is also important for the application of G-DHL that involves a sequence of two operations related to such distinct functions: (a) the application of filters to detect the events of interest (in some cases this operation might be omitted, as discussed below); (b) the application of the G-DHL to the resulting signals encompassing such events. The function of filters related to the generation of events should not be confused with their possible second use to create memory traces and smooth signal with discontinuities, e.g. neural signals with spikes (see the Section ‘Traces: overcoming the time gaps between events’).

**Fig 4:**
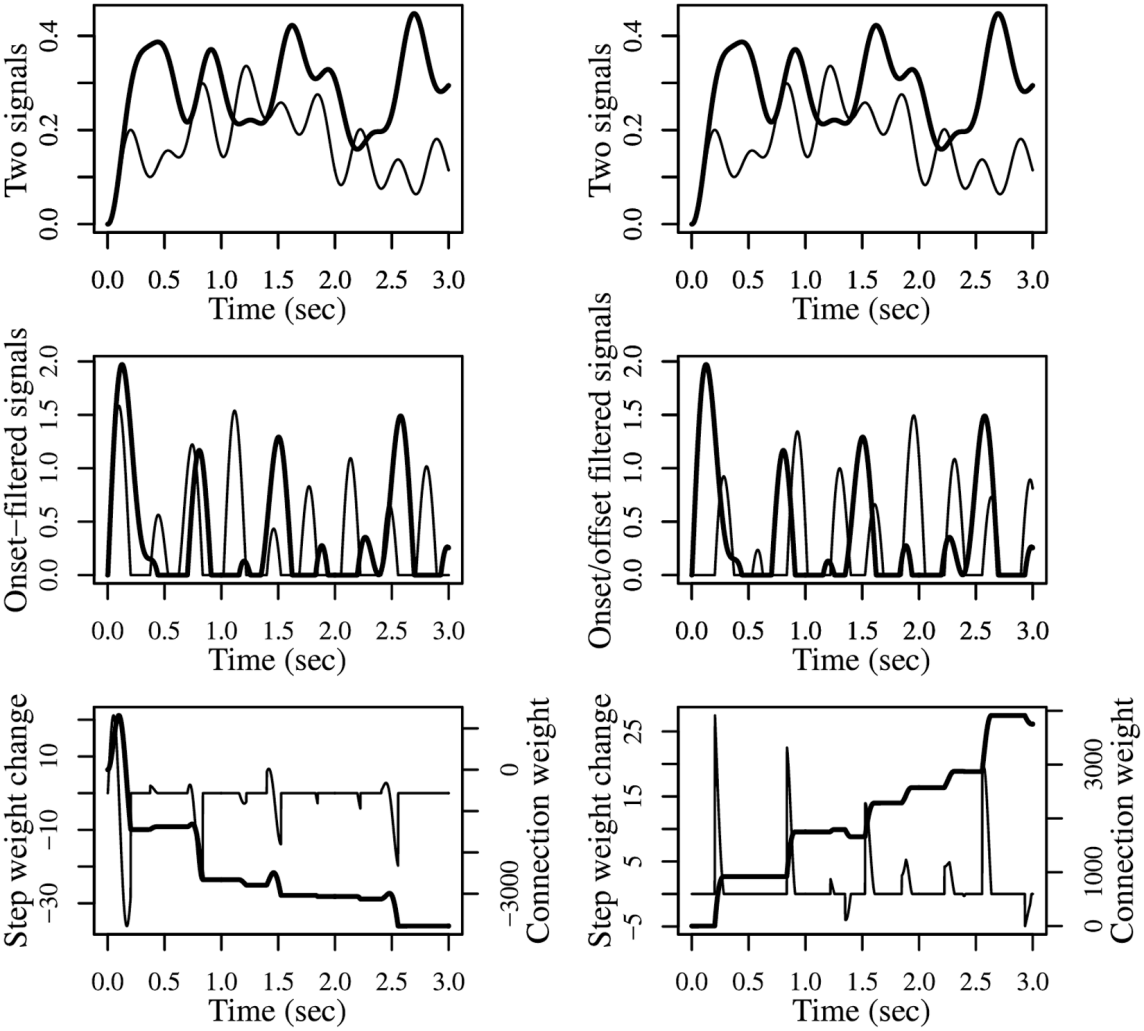
Different filters applied to the same neural signals detect different desired changes and produce different events on which the G-DHL rules can work. The two columns of graphs refer to two different simulations. The simulations start from the same neural signals (top graphs) but use different filters (middle graphs) leading to a different synaptic update even if the same DHL rule is applied (bottom graphs). Top graphs: each graph represents two signals *u*_1_ and *u*_2_ each generated as an average of 4 cosine functions having random frequency (uniformly drawn in [0.1, 3]) and random amplitude (each cosine function was first scaled to (0, 1) and then multiplied by a random value uniformly drawn in (0, 1)). Middle graphs: events resulting from the filters ${\left[{\dot{u}}_{1}\right]}^{+}$ and ${\left[{\dot{u}}_{2}\right]}^{+}$ (left) and from the filters ${\left[{\dot{u}}_{1}\right]}^{+}$ and ${\left[{\dot{u}}_{2}\right]}^{-}$ (right; these filters should not be confused with the analogous filters used within the G-DHL rule). Bottom graphs: step-by-step update of the connection weight (thin curve), and its level (bold curve), obtained in the two simulations by applying the Porr-Wörgötter DHL rule to the filtered signals.

Fig 4 presents the results of two simulations showing how different filters can be applied to the same signals to detect different changes of interest, and how this leads to different synaptic updates even when using the same DHL rule. The example also shows that the G-DHL can be applied to any type of complex signal beyond the simple ones used in previous sections. The two simulations are implemented through three steps: (a) both simulations start from the same pair of signals: these might represent the activation of two firing-rate neural units linked by a connection; (b) the simulations apply different filters to those signals: the first applies a filter to both signals that generates an event for each ‘increase change’; the second applies a filter to the first signal that generates an event for each ‘increase change’, and a filter to the second signal that generates an event for each ‘decrease change’; (c) both simulations then use the same DHL rule to compute the update of the connection weight, here the ‘causal’ Porr-Wörgötter DHL rule (but any other DHL rule might have been used to show the point).

The results show that in the first simulation the connection weight tends to decrease because the increase-changes of the first signal tend to follow the increase-changes of the second signal. In contrast, in the second simulation the connection weight tends to increase as the increase-changes of the first signal tend to anticipate the decrease-changes of the second-signal. Overall, the simulations show how deciding on the filters to use to associate events to the changes of interest is as important as deciding on which DHL rules to use.

The simulations also show how, through the use of suitable filters, one can apply G-DHL to any pair of signals independently of their complexity. The G-DHL can also be directly applied to the initial signals without any pre-filtering, as done in the examples of the Section ‘The G-DHL captures different DHL rules’. In this case the rule will work on the events already present in the signals. G-DHL can even be applied to capture the temporal relations between changes not resembling ‘canonical’ events (i.e., a transient increase followed by a transient decrease). For example, assume there is a first signal having a constant positive value and a second signal that is generally constant but also increases of a random amount at each second. Even if none of the two signals exhibits canonical events, one could still apply some G-DHL rule components to capture some information. For example, the component $\left[u_{1}\right]{\left[{\dot{u}}_{2}\right]}^{+}$ would train a connection weight keeping track of the sum of all increases of the second signal. In general, however, the lack of canonical events prevents a useful application of some G-DHL components (e.g., in the example just considered the G-DHL differential components would leave the connection weight unaltered).

A second observation concerns the fact that in the simulations of Fig 4 we used ${\left[\dot{u}\right]}^{+}$ and ${\left[\dot{u}\right]}^{-}$ as filters to detect events in the signals. These are the same functions used inside the G-DHL rule. This is not by chance. Indeed, when such functions are used inside the G-DHL they are employed to detect two ‘sub-events’ inside the original-signal neural event, namely its ‘increasing part’ and its ‘decreasing part’ that are then temporally related with those of the other signal. This observation suggests that one might generate other versions of the G-DHL rule by using other types of filters, in place of ${\left[\dot{u}\right]}^{+}$ and ${\left[\dot{u}\right]}^{-}$, inside the rule itself.

#### Traces: Overcoming the time gaps between events

A brain or an artificial neural network might need to capture the relations between events separated by a time gap. In this case, G-DHL, like any other learning process, can capture the temporal relation between the events only if the first event leaves some ‘memory representation’ (or ‘eligibility trace’) that lasts after the first event ceases for a time sufficient to overlap at least in part with the second event. Memory traces, obtained with suitable filters, have been largely employed in STDP modeling and machine-learning (e.g. [28, 40]).

**Fig 5:**
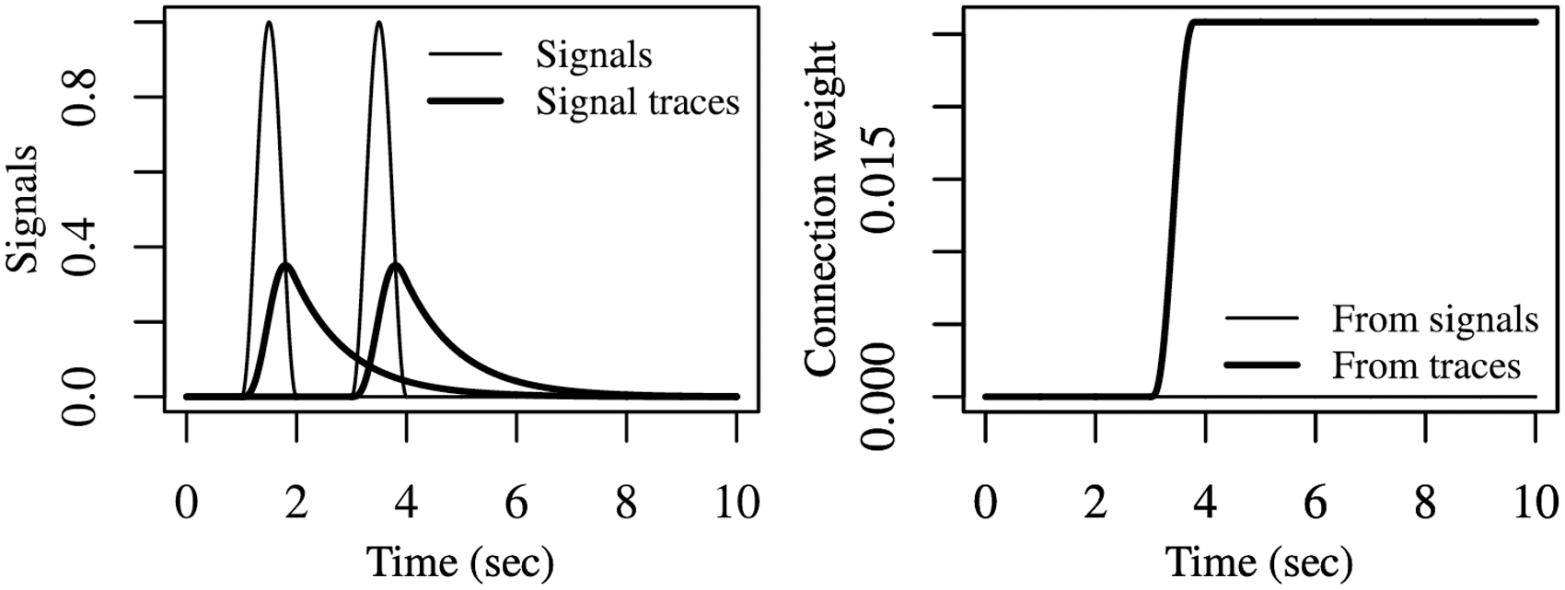
Example of how eligibility traces allow the G-DHL rule to capture temporal interactions between events separated by a time gap. Left: Two neural signals exhibiting an event each, and the related traces. The trace signals *m*_*i*,*t*_ at time step *t* were numerically computed by applying a leaky accumulator process to the initial signals *u*_*i*,*t*_ as follows: *m*_*i*,*t*_ = *m*_*i*,*t*−1_ + (Δ*t*/*τ*) ⋅ (−*m*_*i*,*t*−1_ + *u*_*i*,*t*−1_), with Δ*t* = 0.001 and *τ* = 1. Right: the connection weight resulting from the application of the G-DHL rule component $\left[u_{1}\right]{\left[{\dot{u}}_{2}\right]}^{+}$ to the initial signals or to their memory traces.

Fig 5 shows an example of how DHL rules applied to events separated by a time gap cannot produce a synapse change, whereas they can if applied to memory traces of them. The trace of each event was obtained with a leaky integrator filter (called ‘leaky accumulator’ if discrete time is considered), one of the most popular and simple operators usable to this purpose (other non-memoryless operators might be used to this purpose, [36]). The implementation of traces can be obtained through the same filter functions used to create events. This means that the same filter can be used for two purposes: capturing the events of interest and facing the time-gap problem.

### Using G-DHL to fit STDP data sets

As discussed in the introduction, different types of neurons exhibit surprisingly different STDP learning kernels. For this reason we tested the flexibility of G-DHL by using it to capture several different STDP learning kernels involving pairs of pre- and post-synaptic spikes. In the future G-DHL could be extended to capture STDP processes involving spike triplets or quadruplets ([41]; see [42] for a model) by considering three or more multiplication elements rather than only two as done here.

To apply G-DHL to spike pairs, we first outline the procedure used to derive the formulas to compute G-DHL analytically, rather than numerically as done so far. The procedure is illustrated in detail in Section 2.1 in S1 Supporting Information in the case in which one assumes that spikes and traces are described with some commonly used formulas. Sections 2.7 and 2.8 in S1 Supporting Information show a method that leverages these formulas to use G-DHL to fit STDP data sets; examples of this fitting are shown in the Section ‘Results’.

Before presenting the formulas, we discuss two important points. The closed-form formulas for synaptic updates by the G-DHL rule have two main advantages. First, they allow the mathematical study of the G-DHL rule (see Sections 2.2 and 2.6 in S1 Supporting Information). Second, the formulas allow a computationally fast application of G-DHL by computing the synaptic update through a single formula rather than as a sum of many step-by-step synaptic updates as done in its numerical application, an advantage exploited in the computationally intensive simulations of the Section ‘Results’.

A second observation concerns the relation between the G-DHL explicit formulas and phenomenological models discussed in the introduction. The G-DHL explicit formulas have the form Δ*w* = *f*(Δ*t*) typical of phenomenological models. This shortcut is possible because spikes have a fixed shape: this implies that Δ*t* is the only information relevant for computing G-DHL. The resulting synaptic update is however the same as the one that would be obtained by numerically simulating the step-by-step interaction between the pre- and post-synaptic neural events mimicking more closely what happens in the real brain. Therefore, the possibility of computing Δ*w* = *f*(Δ*t*) formulas for DHL rules does not violate what we said in the introduction, namely that G-DHL captures the mechanisms causing the synaptic update at a deeper level with respect to phenomenological models.

#### Computing G-DHL explicit formulas for spike pairs

The procedure to compute the explicit formulas of G-DHL leads to different results depending on the mathematical expression of the spikes and eligibility traces. The steps of the procedure are however general: (a) decide the mathematical function to represent all spikes and a second function to represent all eligibility traces: this is necessary to abstract over their shape; (b) compute the time derivatives of the eligibility traces: this is necessary to compute the G-DHL components; (c) for each G-DHL component, identify the zero points of the functions corresponding to the pre-/post-synaptic trace signals, and of their derivative positive/negative parts: these points are needed to compute the definite integrals of the next step; (d) for each G-DHL component, formulate the definite integrals (usually 3 to 4) ‘summing up over time’ the instantaneous synaptic update of the component for a given Δ*t*: the lower and upper limits of these integrals depend on the time-overlap between the signal/derivative parts considered by the component and can be different for different Δ*t* values; (e) for each G-DHL component, compute the explicit formulas of its definite integrals, e.g. using a symbolic computation software. Notice how this procedure could be applied to only the sub-set of G-DHL components of interest.

Section 2.1 in S1 Supporting Information applies the procedure to compute all G-DHL component formulas assuming: (a) a spike represented with a *Dirac* *δ*-*function*, as commonly done in the literature [14, 43]; (b) an eligibility trace represented by an *α*-*function*, as often done to model the excitatory post-synaptic potentials (EPSP) evoked by pre-synaptic spikes [14, 43]. Sections 2.2 and 2.4 in S1 Supporting Information present the formulas computed under these conditions in the cases in which the time constants of the eligibility traces, *τ*_1_ and *τ*_2_, either differ or are equal.

**Fig 6:**
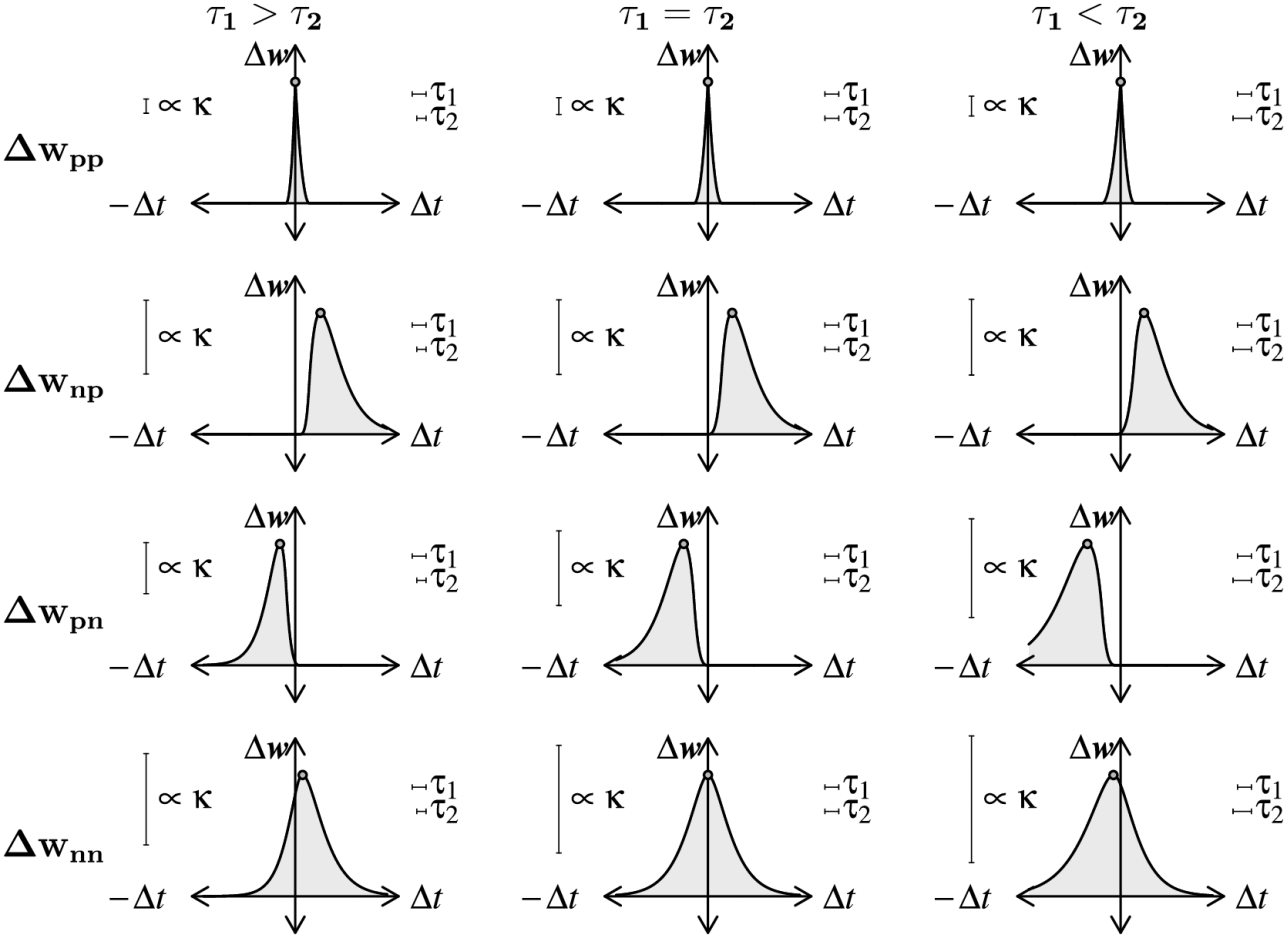
Learning kernels of the four G-DHL differential components for a pair of pre-/post-synaptic spikes. The three columns of graphs refer respectively to: $\tau_{1}=\frac{3}{2}\tau_{2}$; *τ*_1_ = *τ*_2_; $\tau_{1}=\frac{3}{4}\tau_{2}$. The four rows of graphs refer to the G-DHL different components: ‘p’ indicates the positive part of the eligibility-trace derivative and ‘n’ indicates its negative part. Small gray circles indicate maximum synaptic changes.

**Fig 7:**
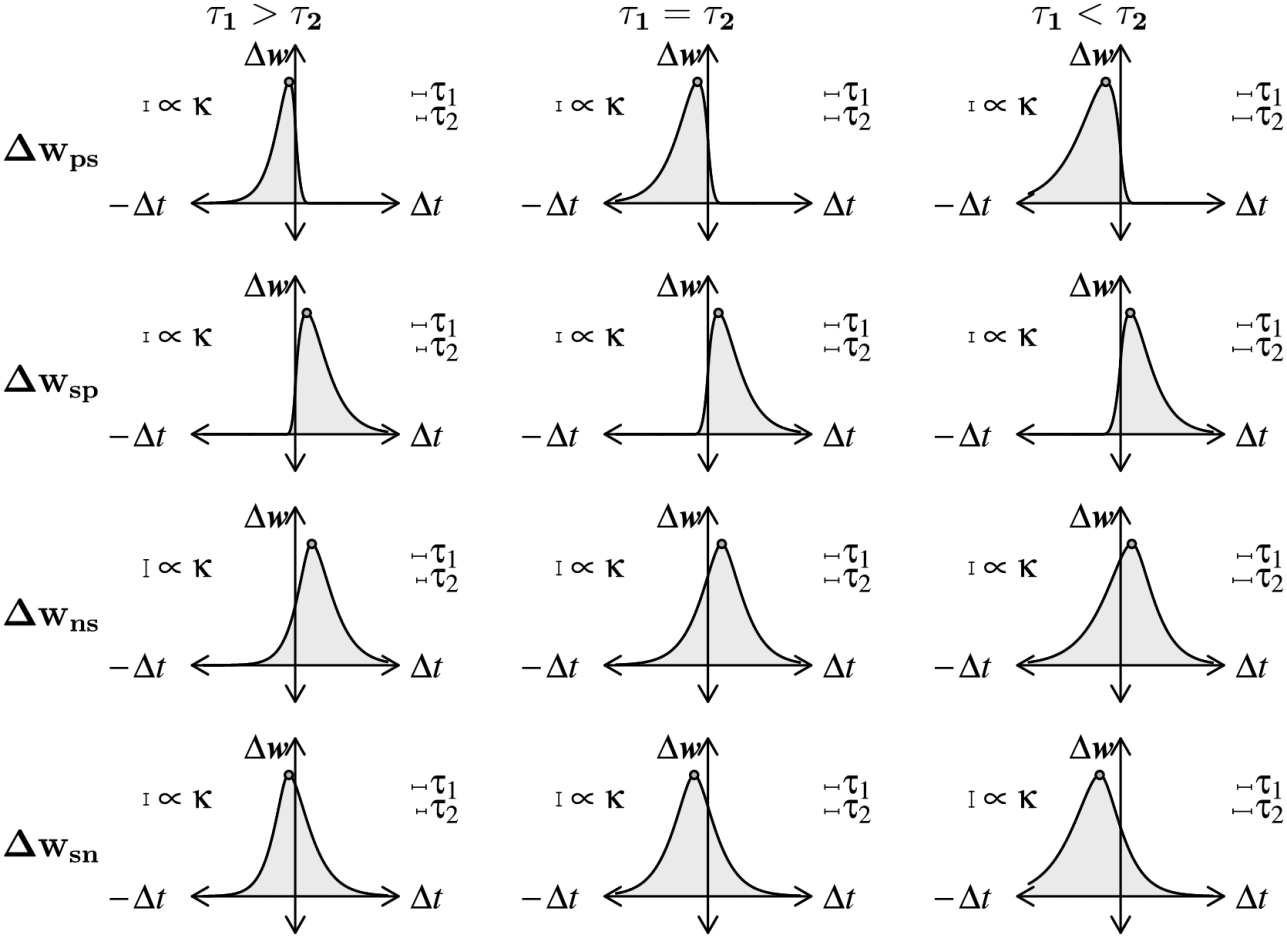
Learning kernels generated by the four mixed components of the G-DHL rule applied to a pair of pre-/post-synaptic spikes. Graphs are plotted as in Fig 6, with ‘s’ indicating the eligibility-trace signal.

The explicit formulas can be used to compute the synaptic update for a given Δ*t*, and hence yield the learning kernels of the G-DHL components, as shown in Figs 6 and 7. For each component, the figures show the case in which *τ*_1_ = *τ*_2_ and also two example cases in which *τ*_1_ > *τ*_2_ and *τ*_1_ < *τ*_2_. In the figures, the *σ* and *η* parameters are both set to +1, thus producing a synaptic enhancement. Negative values would produce a synaptic depression. The maximum of each curve is also shown: this can be computed analytically (see Sections 2.3 and 2.5 in S1 Supporting Information) and marks the delay between the pre- and post-synaptic events causing the maximum change.

The analysis of the figures and formulas indicates that the different G-DHL components have distinct features. These differences are at the basis of the G-DHL capacity to generate different STDP learning kernels and to allow one to select the G-DHL components, or combinations of them, required to obtain different synaptic updates in artificial neural networks. The features can be summarised as follows. The component ‘pp’ is strongly Hebbian, leading to a sharp synaptic update for Δ*t* values close to zero and always peaking at zero. The components ‘np’ and ‘pn’ change the synapse only for respectively Δ*t* > 0 and Δ*t* < 0, and leave it unaltered for Δ*t* values with opposite signs. The component ‘nn’ is Hebbian, like pp, but it has a larger Δ*t* scope; for *τ*_1_ > *τ*_2_ and *τ*_1_ < *τ*_2_ it leads to a maximum synaptic change for respectively positive and negative Δ*t*. The components ‘ps’ and ‘sp’ lead to strong synaptic updates for respectively negative and positive Δ*t* values close to zero, and to a modest synaptic change for Δ*t* values having opposite signs. The components ‘ns’ and ‘sn’ are similar to the previous two components, but in this case they cause a relevant synaptic change for Δ*t* values having opposite signs.

#### Automatic procedure to fit STDP data sets with G-DHL

G-DHL can be used to obtain particular STDP kernels by hand-tuning its parameters, for example to fit STDP data to some degree of approximation. This can be done on the basis of the synaptic updates caused by the different G-DHL components, shown in Figs 6 and 7, and it is facilitated by the linear-combination structure of the rule.

Alternatively, one can employ an automatic procedure to fit the data more accurately. To show this, we used a procedure illustrated in detail in Sections 2.7 and 2.8 in S1 Supporting Information and for which we now provide an overview. For a given STDP data set, the procedure searches for the best combination of the rule components (combinations can have from 1 to 8 components), their parameters *σ* and *η*, and the parameters *κ*, *τ*_1_, and *τ*_2_. The search for the best combination of components employs a model-comparison approach using the *Bayesian information criterion* (BIC; [44]) to ensure an optimal balance between model complexity (number of components, and hence parameters, used) and accuracy of fit. The search for the parameter values is done via a *genetic algorithm* [45] optimising the accuracy of fit as measured by the *fraction of variance unexplained* (FVU).

The Section ‘Results’ shows how this procedure produces an accurate and stable fit of several different STDP data sets. This outcome might appear to be limited by the fact that the G-DHL rule involves many parameters. This is not the case because: (a) G-DHL can be seen as a set of DHL rules corresponding to its eight components; (b) each combination of the G-DHL components (formed by 1 to 8 components) is considered as a single model to perform an independent regression and the model comparison procedure penalises the models using a higher number of parameters; (c) as a consequence, the best model usually has only few components/parameters, about 2 or 3 (in addition to *κ*, *τ*_1_, *τ*_2_).

## Results

### Using G-DHL to fit the STDP data set from Bi and Poo

**Fig 8:**
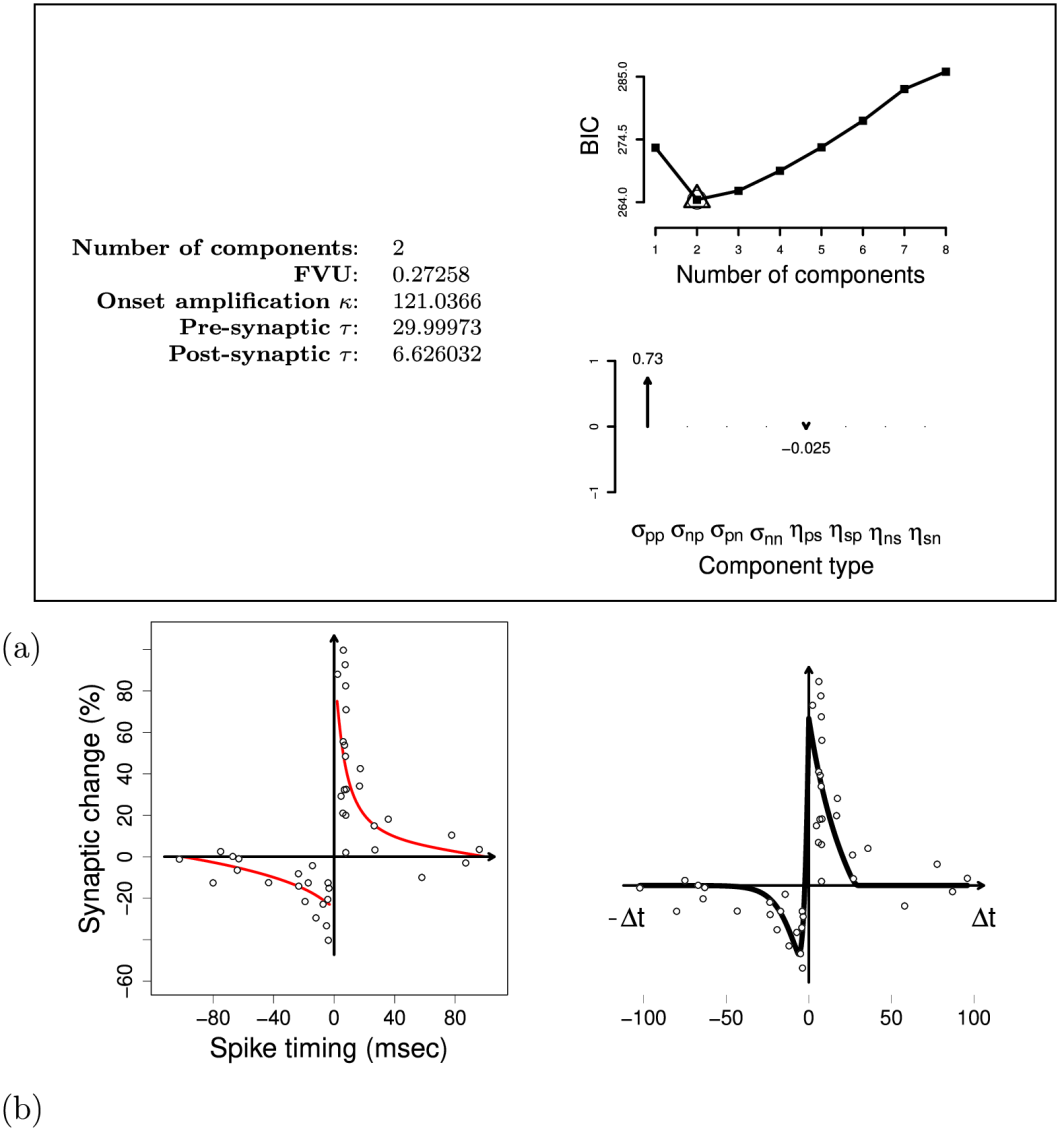
Results of the model comparison and fitting procedures used to regress the classic STDP data set from Bi and Poo [25]. (a) Data on the left: regression results. Top-right graph: BIC values obtained using 1 to 8 G-DHL components. Bottom-right graph: size of the parameters of the selected components. (b) Left graph: data points and exponential regression from [46] (reproduced from data). Right graph: G-DHL fit using the parameters in ‘a’.

The procedure for the automatic fit of STDP data sets was first employed to fit the classic STDP data set of Bi and Poo from rat hippocampal neurons [25]. Fig 8a summarises the results (for ease of reference, henceforth we will refer to synapse strengthening/weakening as ‘LTP—long term potentiation’ and ‘LTD—long term depression’). The model comparison technique selected two G-DHL components: an LTP component (*σ*_*pp*_ = 0.73) and an LTD component (*η*_*ps*_ = −0.025). The parameters *σ* and *η* differ in scale as they refer to differential and mixed G-DHL components involving signal-derivative or derivative-derivative multiplications.

Fig 8b shows the target data and their fit obtained with the G-DHL components and parameters shown in Fig 8a. The G-DHL regression fits the data accurately (*FVU* = 0.2725). While the original paper performed the fit with the usual exponential function for both positive and negative Δ*t*, the G-DHL regression captures the LTP with the *σ*_*pp*_ ‘sharp’ component (Fig 6), concentrated on small positive inter-spike intervals, and the LTD with the *η*_*ps*_ = −0.025 ‘softer’ component (Fig 7), concentrated on negative intervals.

### Searching for biophysical mechanisms underlying STDP

We now illustrate with an example the idea of using the components found by the G-DHL regression to heuristically search for biophysical mechanisms possibly underlying a target STDP data set. This example involves the Bi and Poo’s data set [25] analysed in the previous section. The idea relies on the observation that each multiplication factor of the G-DHL components identified by the regression procedure has a temporal profile that might correspond to the temporal profile of the pre-/post-synaptic neuron electrochemical processes causing the synaptic change.

The steps of the procedure used to search the biophysical mechanisms are as follows: (a) identify with an automatic procedure the G-DHL components and parameters fitting the target STDP data set; (b) define the temporal profile of the two pre-/post-synaptic factors of each found component, and the LTP/LTD effects caused by the component; (c) identify possible biophysical processes having a temporal profile similar to the one of the identified factors; (d) design experiments to verify if the hypothesised biophysical processes actually underlie the target STDP phenomenon in the brain. We now give an example of how to apply the steps ‘a’ and ‘b’, and some initial indications on the step ‘c’, in relation to the Bi and Poo’s data set [25]. The example aims to only furnish an illustration of the procedure, not to propose an in-depth analysis of this STDP data set.

Regarding step ‘a’, Fig 8 shows that the G-DHL regression identified two LTP and LTD components.

**Fig 9:**
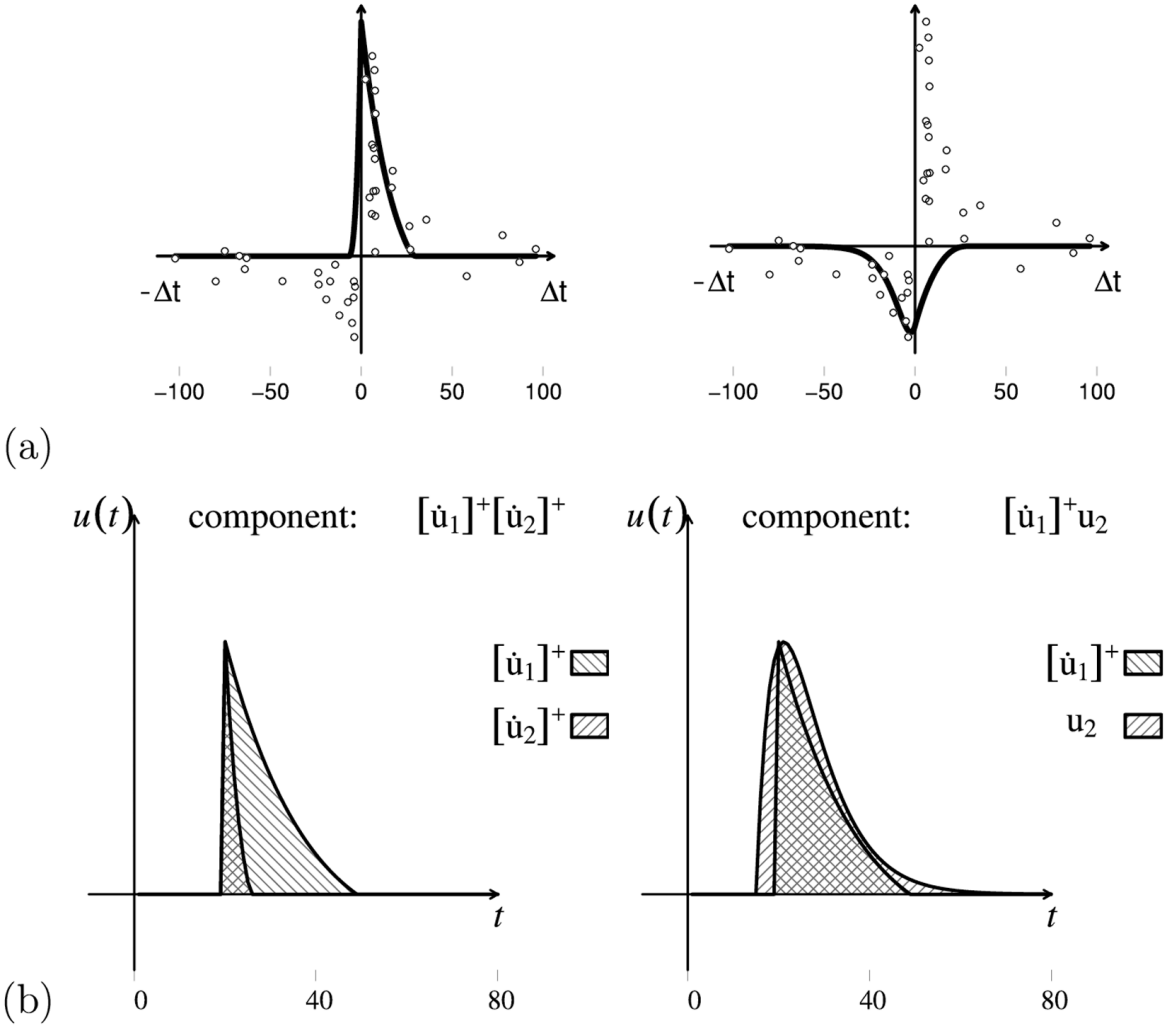
G-DHL components, and related factors, for the Bi and Poo’s learning kernel. (a) Components found by the G-DHL regression of the Bi and Poo’s data set [25]. (b) Temporal profile of the factors of the components shown in ‘a’, plotted for the Δ*t* that causes the maximum synaptic change.

Regarding step ‘b’, Fig 9 shows the temporal profile of the factors of the two components. The first component is a ‘positive-derivative/positive-derivative’ component (${\left[{\dot{u}}_{1}\right]}^{+}{\left[{\dot{u}}_{2}\right]}^{+}$; Fig 9a, left graph) with two factors (Fig 9b, left graph): (a) a relatively long pre-synaptic factor (${\left[{\dot{u}}_{1}\right]}^{+}$) lasting about 30 ms; (b) a shorter post-synaptic factor (${\left[{\dot{u}}_{2}\right]}^{+}$) lasting about 7 ms. These two factors, amplified by a positive coefficient (*σ*_*pp*_ = + 0.73), produce LTP concentrated on small positive inter-spike intervals (0 ms < Δ*t* < 30 ms; Fig 9a, left graph).

The second component is a ‘positive-derivative/signal’ component (${\left[{\dot{u}}_{1}\right]}^{+}u_{2}$; Fig 9a, right graph) with other two factors (Fig 9b, right graph): (a) a relatively long pre-synaptic factor (${\left[{\dot{u}}_{1}\right]}^{+}$) lasting about 30 ms; (b) a longer post-synaptic factor (*u*_2_) lasting about 50 ms. The two factors, amplified by a negative coefficient (*η*_*ps*_ = −0.025), produce LTD covering negative-positive inter-spike intervals (−30*ms* < Δ*t* < 20*ms*; see Fig 9a, right graph).

When the two components are summed, LTP more than cancels out LTD for positive delays (0*ms* < Δ*t* < 20*ms*). This causes the sharp passage from LTD to LTP around the critical Δ*t* values close to zero, which characterise the target kernel (Fig 8).

Regarding step ‘c’ of the procedure, directed to identify possible biological correspondents of the component factors identified in step ‘b’, we now discuss some possible candidate mechanisms that might underlie the factors identified for the Bi and Poo’s data set. Note that these brief indications are only intended to show the possible application of the procedure, not to make any strong claim on the possible specific mechanisms underlying such STDP data set.

Pioneering studies on hippocampus have shown that a repeated stimulation of the perforant path fibres enhances the population response of downstream dentate granulate cells (*long-term potentiation–LTP*; [47–49]). LTP also takes place in other parts of brain such as the cortex [50], amygdala [51], and the midbrain reward circuit [52]. Other studies have shown the existence of *long-term depression* (*LTD*), complementary to LTP, in various parts of brain, for example hippocampus [53, 54] and motoneurons [55]. More recent research has shown that LTP and LTD, and their intensity, depend on the duration of the *temporal gap* separating the pre- and post-synaptic spikes (*spike time-dependent plasticity—STDP*; e.g. [56], see [18] for a review). The relation between the time-delay and the synaptic change depends on the types of neurons involved (e.g., glutamatergic vs. GABAergic neurons [57, 58]), the position of the synapse (e.g., [59]), and the experimental protocols used (e.g., [60]).

Early findings that blocking NMDA receptors (NMDARs) can prevent both LTP and LTD, while a partial blocking can turn an LTP effect into an LTD, has led to the proposal of several calcium-based models of synaptic plasticity (e.g., [61–64]). One view proposes that *two independent mechanisms* can account for the classic STDP learning kernel [19, 65]. This is in line with the two components, and their factors, found by our G-DHL based regression of Bi and Poo data set. The first component was an LTP ‘positive-derivative/positive-derivative’ component (${\left[{\dot{u}}_{1}\right]}^{+}{\left[{\dot{u}}_{2}\right]}^{+}$) formed by two factors. The first factor was a pre-synaptic factor (${\left[{\dot{u}}_{1}\right]}^{+}$) lasting about 30 ms, compatible with a short-lived effect involving the pre-synaptic glutamatergic neuron spike and affecting the post-synaptic NMDARs [66]. The second factor was a post-synaptic factor (${\left[{\dot{u}}_{2}\right]}^{+}$) lasting about 7 ms, compatible with a back-propagating action potential (BAP; [67]). The second component was a ‘positive-derivative/signal’ LTD component (${\left[{\dot{u}}_{1}\right]}^{+}u_{2}$) formed by two factors: a relatively slow pre-synaptic element, (${\left[{\dot{u}}_{1}\right]}^{+}$), lasting about 30 ms, and a slow post-synaptic element, (*u*_2_), lasting about 50 ms. Different biological mechanisms might underlie these two factors. In this respect, there is evidence that post-synaptic NMDARs might not be necessary for spike-timing-dependent LTD [68], while this might be caused by metabotropic glutamate receptors (mGluR; [69]), voltage gated calcium channels (VGCC; [25, 69]), pre-synaptic NMDAR [70], or cannabinoid receptors [68, 69].

### Modelling different STDP classes with G-DHL

We tested the generality of G-DHL by fitting all STDP kernels reported in the review of Caporale and Dan [18]. The data sets addressed in this review encompass many different STDP experiments reported in the literature and proposes a taxonomy to group them into distinct, and possibly exhaustive, classes. The taxonomy is first based on the excitatory or inhibitory nature of the pre- and post-synaptic neurons, giving the classes: (a) excitatory-excitatory; (b) excitatory-inhibitory; (c) inhibitory-excitatory; (d) inhibitory-inhibitory. Some neurons in different parts of brain belong to the same class but exhibit different STDP learning kernels: in [18], these have been grouped in ‘subtypes’ (sub-classes) called ‘Type I’, ‘Type II’, etc.

**Fig 10:**
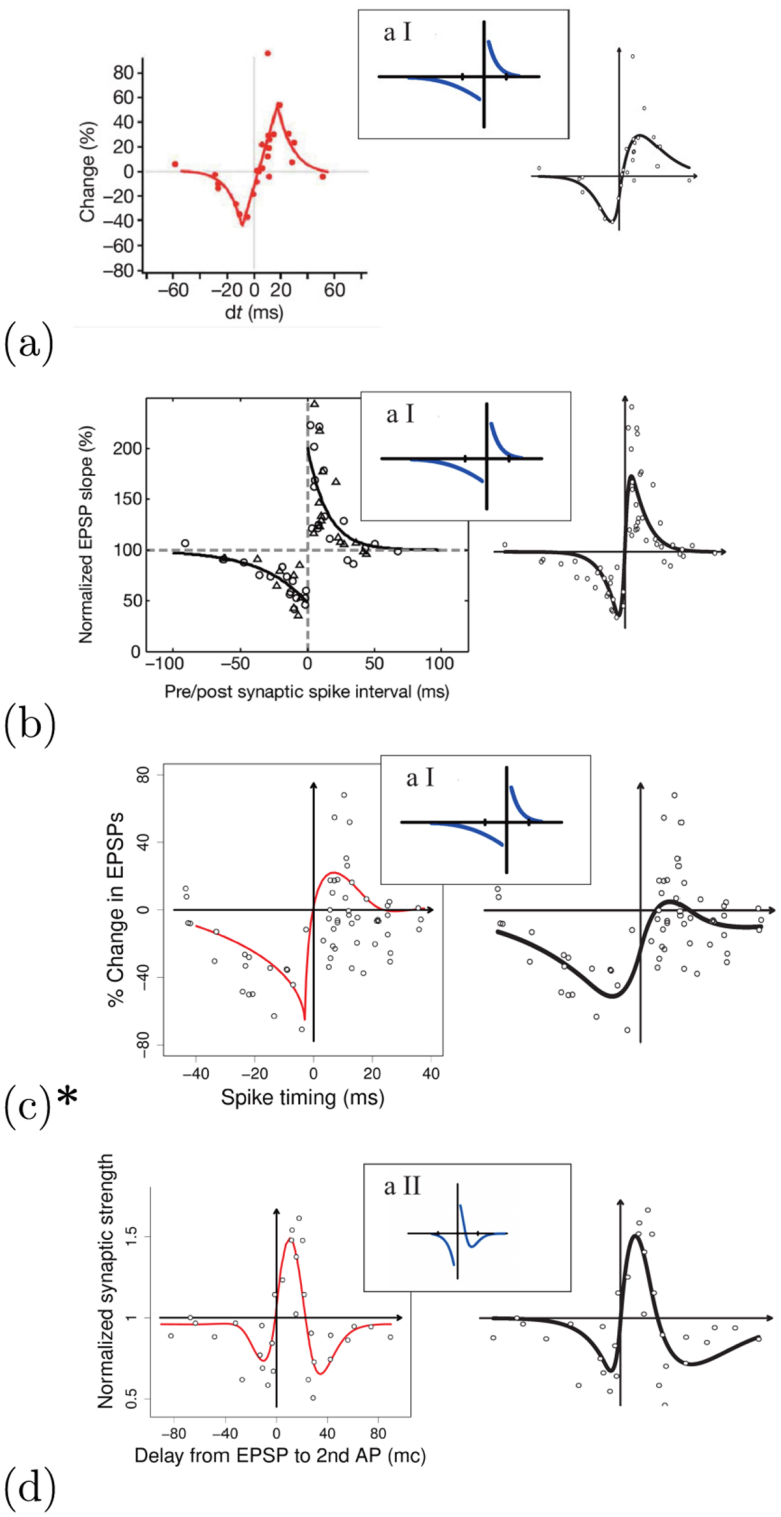
Different STDP data sets, representative of typical STDP learning kernels, fitted with the G-DHL rule. Each group of graphs refers to one STDP class/subtype and shows: (1) left graph: data and fitting curve from the original article (in ‘a’ and ‘b’: reprinted with permission from respectively [71] and [72]; in ‘c’ and ‘d’: reproduced from data and graphs published in respectively [73] and [74]); (2) right graph: data and fitting curve obtained with the G-DHL regression; (3) top-central graph: learning curve suggested in [18] to capture the STDP kernel (reprinted with permission). When available, the G-DHL regression was based on the original data (graphs with a star: *), otherwise it used the data extrapolated from the published graphs: (a) data extrapolated from [71]; (b) data extrapolated from [72]; (c) original data from [73]; (d) data extrapolated from [74]. Section 3 in S1 Supporting Information presents more detailed data on the regressions as in Fig 8.

**Fig 11:**
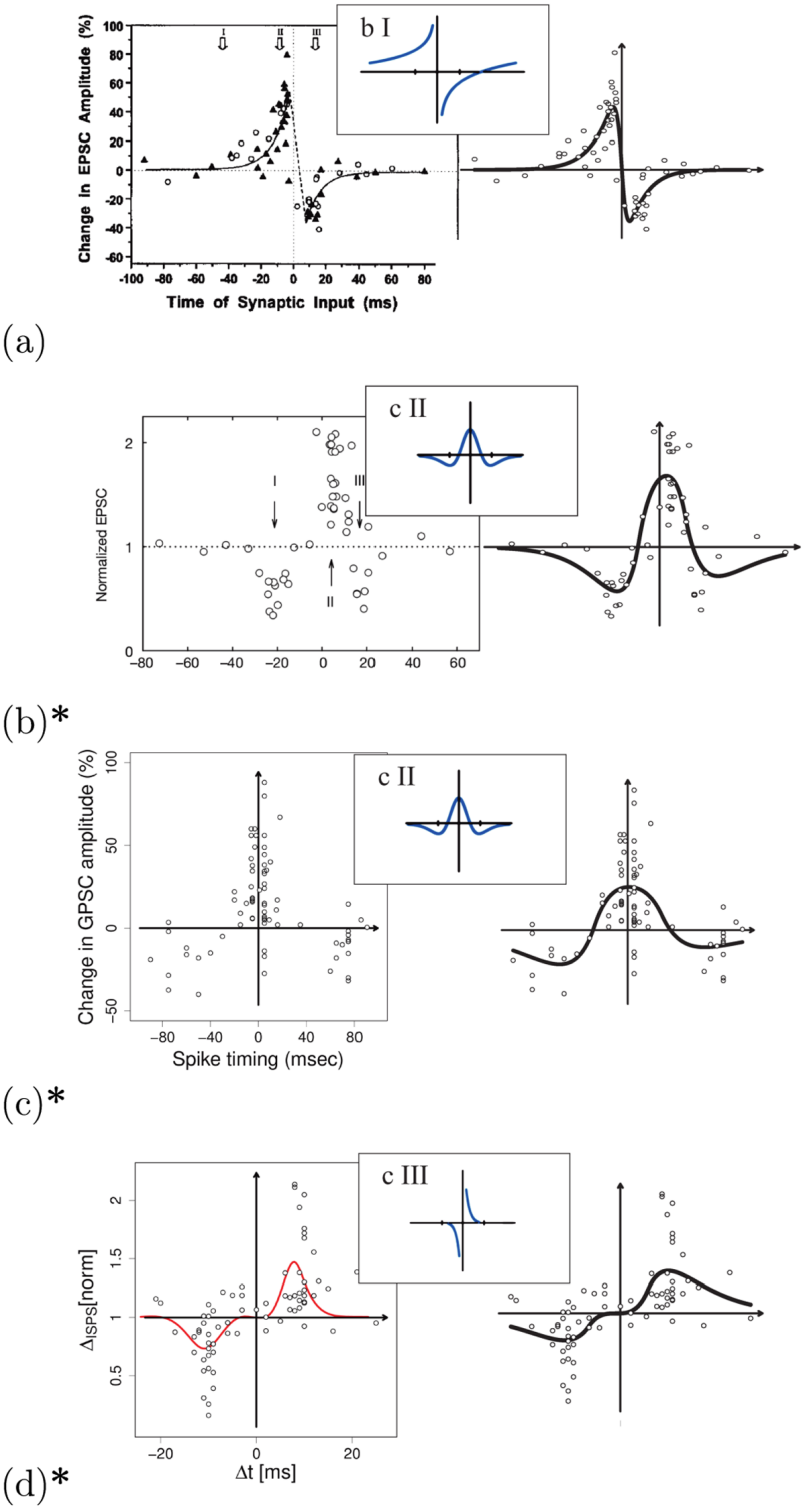
Other STDP data sets and classes/types fitted with G-DHL. Graphs plotted as in Fig 10 (graphs on the left in ‘a’ and ‘b’: reprinted with permission from respectively [76] and [77]; graphs on the left in ‘c’ and ‘d’: reproduced from data and graphs from respectively [78] and [79]). Right graphs: (a) data extrapolated from [76]; (b) original data from [77]; (c) original data from [78]; (d) original data from [79]. Section 3 in S1 Supporting Information presents more detailed data on the regressions as in Fig 8.

For the G-DHL regressions we used the original data when the authors of the experiments could furnish them. When this was not possible, we used the data extracted from graphs in the publications. Figs 10 and 11 summarise the outcome of the G-DHL-based regressions for the different data sets. For each data set, the figures report this information: (a) left graph: original data and, when available, regression curve of the original paper; (b) right graph: regression curve based on G-DHL; (c) top-center small graph: function with which the review [18] proposed to represent the STDP class of the data set. In the following, we illustrate the salient features of these regressions. Section 3 in S1 Supporting Information presents more detailed data on all the regressions as those presented in Fig 8 for the data set of Bi and Poo.

#### Excitatory-excitatory synapses

Fig 10 addresses data sets published in [71] (Kenyon cells onto downstream targets in locust), [72] (pyramidal neurons in layer 2/3 of rat visual cortical slices), [73] (pyramidal neurons in layer 2/3 of rat entorhinal cortex), and [74] (rat hippocampus CA1-CA3 synapses). Based on the similarity of the curves, Caporale and Dan [18] proposed to group the first three data sets into a first sub-type corresponding to the classic STDP kernel modelled with two exponential curves. Instead, they classified the fourth data set as a second sub-type captured with an exponential function for negative inter-spike intervals and a positive/negative function for positive intervals. Exponential fitting curves were also used by the authors of the second data set [72] (Fig 10b). Instead, for the first data set [71] (Fig 10a) the authors used a piece-wise regression based on three components, namely two exponential curves connected by a linear segment centred on *t* = +4 ms. For the third data set [73] (Fig 10c), the authors used a specific biophysical model capturing the effects on STDP of spike width, two-amino-5-phosphonovalerate (APV), and nifedipine. For the fourth data set [74], (Fig 10d) the authors used a difference between two Gaussian functions generating a ‘Mexican-hat’ kernel. Notice the heterogeneity of the approaches used to fit the different data sets, suggested by their different features.

The G-DHL-based automatic regression applied to the four data sets found two components (hence parameters) for each of them: data set of Fig 10a: *η*_*ps*_ = −0.47 and *η*_*ns*_ = 0.66; data set of Fig 10b: *η*_*ns*_ = −0.60 and *η*_*sp*_ = 0.66; data set of Fig 10c: *η*_*ns*_ = −0.4 and *η*_*sp*_ = 0.27; data set of Fig 10d: *η*_*ns*_ = −0.38 and *η*_*sp*_ = 0.17. The fitting of the four data sets show how the procedure can capture all data sets with the G-DHL smooth kernels, even in cases where the data show a sharp passage between LTD and LTD (e.g., see Fig 10b). The results also suggest interesting possibilities with respect to Caporale and Dan’s classification [18]. The first data set is characterised by LTD for negative spike delays and LTP for positive ones. Accordingly, the G-DHL-based regression found two components (the first, ${\left[{\dot{u}}_{1}\right]}^{+}u_{2}$, with a negative coefficient *η*_*ps*_ = −0.47; the second, ${\left[{\dot{u}}_{1}\right]}^{-}u_{2}$, with a positive coefficient *η*_*ns*_ = 0.66) producing LTD and LTP effects for respectively negative and positive spike delays (see also Fig 7). Instead, for the second, third, and fourth data sets the regression identified the same components, ${\left[{\dot{u}}_{1}\right]}^{+}u_{2}$ and ${\left[{\dot{u}}_{1}\right]}^{-}u_{2}$, notwithstanding the different graphical appearance of their kernels. A closer consideration of these three data sets shows the reason of this. The three cases tend to exhibit an LTD-LTP-LTD sequence (the latter LTD covers ‘large’ *positive* delays). This is caused by an LTP component (${\left[{\dot{u}}_{1}\right]}^{-}u_{2}$) having a narrow temporal scope fully contained within the larger temporal scope of the LTD component (${\left[{\dot{u}}_{1}\right]}^{+}u_{2}$). Since the synaptic changes of the two components sum, the resulting learning kernel shows LTD for negative and for large positive spike delays, and an intermediate LTP for small positive delays (cf. [75]).

These results prompt two observations. First, there might actually be two distinct biophysical mechanisms underlying the last three data sets, with LTD spanning beyond LTD for both negative and positive inter-spike intervals. Second, G-DHL might be used to classify STDP kernels based on their underlying components rather than their graphical appearance. For example, the four data sets discussed above would be clustered into two subtypes, one encompassing the first data set and the second encompassing the last three data sets. This classification would separate STDP data sets involving ‘classic’ LTD-LTP kernels (first group) and more sophisticated LTD-LTP-LTD kernels showing a ‘pre-post LTD’ for large positive spike delays in addition to the standard LTD for negative delays [74, 75]. These might suggest experiments to seek the biophysical mechanisms actually underlying the different STDP kernels.

#### Excitatory-inhibitory synapses

Fig 11a addresses a data set from [76] belonging to the second STDP class proposed by Caporale and Dan [18] (neurons from tadpole tectum). The kernel mirrors, with respect to the x-axis, the excitatory-excitatory case (Fig 10b). This means that the synapse is enhanced when the pre-synaptic spike follows the post-synaptic spike, and is depressed in the opposite condition. The G-DHL regression produced two components/parameters: *η*_*sp*_ = −.65 and *η*_*ns*_ = .61. These are the same components that the algorithm used for the data sets of Fig 10b–10d, but with opposite signs: it would be interesting to consider the actual biophysical mechanisms corresponding to the two different STDP kernels to evaluate if they share some relations. Notice how the model has no problem in capturing the steep part of the data curve around Δ*t* = 0. Instead, the authors of the original paper used two exponential curves to fit the data but had to ignore the data points around Δ*t* = 0 where such curves get −∞ or + ∞ values [76].

#### Inhibitory-excitatory synapses

Fig 11b and 11c refers to data sets related to the third STDP class of Caporale and Dan’s taxonomy [18]. Data of Fig 10b are from [77] (neurons from rat CA1 hippocampus region) and data of Fig 10c are from [78] (culture neurons of embryonic rat hippocampal). The original papers did not fit the data whereas in [18] they are fitted with a ‘Mexican hat’ function. The G-DHL regression captured both data sets with the same three components confirming their class consistency: first data set, *σ*_*np*_ = −.52, *σ*_*pn*_ = −.48, and *σ*_*nn*_ = .77; second data set, *σ*_*np*_ = −.36, *σ*_*pn*_ = −.53, and *σ*_*nn*_ = .63. The two components of *σ*_*np*_ and *σ*_*pn*_ are symmetric with respect to the y-axis (Fig 6) and, having a negative sign, cause the two LTD parts of the target STDP kernels for ‘large’ negative and ‘large’ positive Δ*t* values. The *σ*_*nn*_ component is instead centred on Δ*t* = 0 and, having a positive sign, is responsible for the LTP central part of the kernels.

#### Inhibitory-inhibitory synapses

Finally, Fig 11d addresses the STDP data set presented in [79] (neurons of the rat entorhinal cortex). To fit the data the authors used a function based on the two usual exponential functions but multiplied them by (Δ*t*)^10^ to have a low STDP around Δ*t* = 0. Instead, Caporale and Dan [18] proposed to capture this kernel with the standard exponential model also used for the excitatory-excitatory class. In line with the regression used by the authors of the original paper [79], the G-DHL rule found components different from the excitatory-excitatory class: *σ*_*np*_ = .78 and *σ*_*pn*_ = −.56. These components (Fig 6) can generate the zero-level plateau shown by the data in proximity of Δ*t* = 0, for which the kernel does not update the synapse for null or small positive/negative time intervals. The G-DHL regression thus suggests that this particular feature, not captured by exponential functions, might characterise a different STDP type.

#### A new STDP taxonomy

Overall, the regressions based on G-DHL suggest the existence of different STDP classes with respect to those proposed in [18]. These classes are summarised in Table 1 and might be useful to guide a systematic search for the biophysical mechanisms underlying different STDP phenomena.

**Table 1:** Summary of the regressions of the nine STDP data sets regressed with the G-DHL rule. The table indicates: the species and brain area from which the neurons have been taken (Hip: hippocampus; VisCtx: visual cortex; EntCtx: enthorinal cortex; Tec: Tectum); the reference where the data were published (Ref.); the parameters of the G-DHL selected model (i.e., the 2 or 3 parameters of the components of the model chosen by the model comparison technique); the type of pre- and post-synaptic neuron (Exc: excitatory; Inh: inhibitory); the taxonomy with which the STDP data set has been classified in Caporale and Dan [18] (C.&D. classes); our taxonomy proposed on the basis of the components found by the G-DHL regression. Our classes: ‘E’ and ‘I’ refer to the excitatory/inhibitory neurons involved, specifying the class, and the numbers refer to the subtypes within the class.

Species	Brain Area	Ref.	Component								Neurons		C.&D. classes	Our classes
			*σ*_*pp*_	*σ*_*np*_	*σ*_*pn*_	*σ*_*nn*_	*η*_*ps*_	*η*_*sp*_	*η*_*ns*_	*η*_*sn*_	In	Out		
Rat	Hip	[25]	+.73				-.02				Exc	Exc	A I	EE 1
Locust	Kenion	[71]					-.47		+.66		Exc	Exc	A I	EE 2
Rat	VisCtx	[72]						+.66	-.60		Exc	Exc	A I	EE 3
Rat	EntCtx	[73]						+.27	-.40		Exc	Exc	A I	EE 3
Rat	Hip	[74]						+.17	-.38		Exc	Exc	A II	EE 3
Tadpole	Tec	[76]						-.65	+.61		Exc	Inh	B I	EI 1
Rat	Hip	[77]		-.52	-.48	+.77					Inh	Exc	C II	IE 1
Rat	Hip	[78]		-.36	-.53	+.63					Inh	Exc	C II	IE 1
Rat	EntCtx	[79]		+.78	-.56						Inh	Inh	C III	II 1

## Discussion

Understanding the functioning and learning in dynamical neural networks is challenging but also very important for advancing our theories and models of the brain—an exquisitely dynamical machine. Differential Hebbian Learning (DHL) might become a fundamental means to do so. Existing DHL rules are few, basically two [5, 7], and are not able to model most spike-timing dependent plasticity (STDP) phenomena found so far in the brain. Building on previous pioneering research, this work addresses these limitations in multiple ways. First, it proposes a framework to understand, use, and further develop DHL rules. In particular, it proposes a general DHL (G-DHL) rule encompassing existing DHL rules and generating many others, and highlights key issues related to the pre-processing of neural signals before the application of DHL rules. Second, it proposes procedures and formulas for applying DHL to model STDP in the brain. Third, it shows how the proposed G-DHL rule can model many classes of STDP observed in the brain and reviewed in [18].

With respect to other approaches for modelling STDP, DHL represents a complementary tool in the toolbox of the modeller and neuroscientist. First, DHL differs from ‘phenomenological models’. Although simple and elegant, these models update the synapse based on mathematical functions directly mimicking the synaptic changes observed in empirical experiments in correspondence to different inter-spike intervals [14, 15]. Instead, DHL rules compute the synaptic update on the basis of the step-by-step interactions between levels of and changes in the neural variables of interest. DHL rules also differ from ‘biophysical models’. These models can reproduce many biological details but have high complexity and rely on phenomenon-specific mechanisms (e.g., [14, 17]). Instead, DHL rules reproduce fewer empirical details but at the same time, after the systematisation proposed here, they represent ‘universal mechanisms’ able to capture many STDP phenomena.

G-DHL relies on two main ideas. The first idea, elaborated starting from previous proposals [5] (see also [29]), is that the derivative of an ‘event’, intended as a monotonic increase followed by a monototic decrease of a signal, gives information on when the event starts and terminates. This information is used by G-DHL to update the connection weight depending on the time interval separating the pre- and post-synaptic neural events. The second idea is that the actual synaptic update can rely on different combinations of the possible interactions between the pre-/post-synaptic events and their derivatives, thus leading to a whole family of DHL rules.

Mathematically, this gives rise to a compound structure of the G-DHL rule which is formed by a linear combination of multiple components. In this respect, the capacity of G-DHL to capture different STDP phenomena is linked to the power of kernel methods used in machine learning [34, 35]. The linear form of the rule facilitates its application through manual tuning of its parameters, as shown here and in some previous neural-network models of animal behaviour using some components of the rule [80–82]. The linear form of the rule also facilitates the automatic estimation of its coefficients when used to capture STDP data sets, as also shown here.

G-DHL has a high expressiveness, as shown here by the fact that we could use it to accurately fit multiple STDP data sets. In particular, the G-DHL components form basis functions that are well suited to model key aspects of STDP, in particular its long-term potentiation/depression features, its time span, and its variability around the zero inter-spike interval (e.g., sharp depression-potentiation passages, non-learning plateau, Hebbian/anti-Hebbian learning). The regressions of the data sets targeted here employed seven out of eight components of the rule. The regressions are particularly reliable because the optimisation procedure used here is highly robust with respect to local minima, so they show the utility of most G-DHL components for modelling different STDP data sets. Future empirical experiments might search for STDP processes corresponding to the eighth non-used G-DHL component (encompassing a multiplication between the pre-synaptic stimulus and the post-synaptic derivative negative part): this corresponds to a relatively long LTD peaking at a negative inter-spike interval but also involving low-value positive intervals.

The results of our regression based on G-DHL of the classic STDP kernel, represented by the classic Bi and Poo data set [25], suggests the possible existence of two distinct mechanisms underlying LTP and LTD involved in such STDP learning kernel, so it is interesting to compare this result with different views in the literature. A specific hypothesis on calcium control of plasticity was formulated in [83] and was followed by significant experimental evidence. According to this hypothesis, post-synaptic calcium transients above a lower threshold cause LTD whereas calcium transients above a second higher threshold produce LTP. In a detail model [84], this phenomenon is captured with a single mechanism for which the synaptic change is caused by calcium concentrations at the post-synaptic neuron modulated by the temporal relation between the current at the pre-synaptic neuron (causing NMDAR opening) and the back-propagating action potential (BAP) at the post-synaptic neuron [67]: low levels of post-synaptic calcium cause the synapse depression whereas high levels cause its enhancement. Models of such type have been criticised on the basis of empirical evidence. According to [65], calcium models require a long-fading BAP-induced transients to account for LTD when the BAP occurs before the pre-synaptic action potential [12]. Moreover, calcium models also predict a pre-post form of LTD even when the BAP occurs beyond a given time from the pre-synaptic action potential. While this pre-post form of LTD has been registered in hippocampal slices [74], other data [25] indicate that it is not a general feature of STDP. In this respect, our findings agree with other proposals for which *two* independent mechanisms account for LTP and LTD in the classic STDP learning kernel [19, 65]. Future work might extend these preliminary results. In particular, it could aim to understand in detail how some of the mechanisms mentioned above implement *change detectors* and these lead to STDP, as predicted by the G-DHL core functioning mechanisms based on *derivatives*. Moreover, G-DHL could be used to heuristically guide the identification of the biophysical mechanisms underlying different STDP data sets beyond the classic kernel.

Future work might also investigate, both computationally and empirically, DHL rules different from G-DHL, namely: (a) DHL rules formed by three or more components (useful to model STDP involving more than two spikes [41]); (b) DHL rules using orders of derivatives higher than the first one used in G-DHL [32, 33]; (c) DHL rules generated by other types of filters, rather than ${\left[\dot{u}\right]}^{+}$ and ${\left[\dot{u}\right]}^{-}$ used in G-DHL, to detect the increasing and decreasing parts of events.

Another line of research might aim to investigate the possible computational and behavioural functions of the different G-DHL components. In this respect, the analysis presented here on the computational *mechanisms* underlying STDP might contribute to the current research on the possible *functions* of such plasticity [20–23]. Indeed, this research mainly focuses on the computational function of the classic STDP learning kernel [25], whereas the research presented here, by stressing how the brain uses different DHL rules, calls for the investigation of their different possible functions.

A different approach to understand the functions of different DHL rules and STDP kernels might use embodied neural models to understand their utility to support adaptive behaviour. The development of G-DHL was in fact inspired by the need to implement specific learning processes in neural-network models able to autonomously acquire adaptive behaviours [80–82]. Thus, it could for example be possible to establish a particular target computation or behaviour and then automatically search (e.g. with genetic algorithms or other optimisation techniques) the rule components and coefficients that are best suited for them. For example, previous work [85] used a learning rule based on Kosco’s DHL rule [5] to obtain interesting/surprising emergent behaviours in physical simulated agents. This approach might test other G-DHL components to produce different behaviours.

## Supporting information

S1 Supporting Information All supporting information of the article is contained in a pdf file with such name. (PDF)
