# Supplementary material for: General differential Hebbian learning: Capturing temporal relations between events in neural networks and the brain
Source: PLoS Comput Biol. 2018 Aug 28;14(8):e1006227. doi: 10.1371/journal.pcbi.1006227 (PMC6130884; doi:10.1371/journal.pcbi.1006227)
Supplement: S1 Supporting Information — (PDF) [file pcbi.1006227.s001.pdf]

Supporting Information of the Article: General Differential Hebbian Learning: Capturing Temporal Relations between Events in Neural Networks and the Brain

Stefano Zappacosta, Francesco Mannella, Marco Mirolli, Gianluca Baldassarre*

Laboratory of Computational Embodied Neuroscience,
Institute of Cognitive Sciences and Technologies,
National Research Council of Italy (LOCEN-ISTC-CNR),
Via San Martino della Battaglia 44, I-00185 Roma, Italy

* Corresponding author: Gianluca Baldassarre
Email: gianluca.baldassarre@istc.cnr.it
Learning, differential Hebb rules, continuous-time artificial neural networks, brain, spike-timing-dependent plasticity.

Abstract

Learning in biologically relevant neural-network models usually relies on Hebb learning rules. The typical implementations of these rules change the synaptic strength on the basis of the co-occurrence of the neural events taking place at a certain time in the pre- and post-synaptic neurons. Differential Hebbian learning (DHL) rules, instead, are able to update the synapse by taking into account the temporal relation, captured with derivatives, between the neural events happening in the recent past. The few DHL rules proposed so far can update the synaptic weights only in few ways: this is a limitation for the study of dynamical neurons and neural-network models. Moreover, empirical evidence on brain spike-timing-dependent plasticity (STDP) shows that different neurons express a surprisingly rich repertoire of different learning processes going far beyond existing DHL rules. This opens up a second problem of how capturing such processes with DHL rules. Here we propose a general DHL (G-DHL) rule generating the existing rules and many others. The rule has a high expressiveness as it combines in different ways the pre- and post- synaptic neuron signals and derivatives. The rule flexibility is shown by applying it to various signals of artificial neurons and by fitting several different STDP experimental data sets. To these purposes, we propose techniques to pre-process the neural signals and capture the temporal relations between the neural events of interest. We also propose a procedure to automatically identify the rule components and parameters that best fit different STDP data sets, and show how the identified components might be used to heuristically guide the search of the biophysical mechanisms underlying STDP. Overall, the results show that the G-DHL rule represents a useful means to study time-sensitive learning processes in both artificial neural networks and brain.

Author Summary

Which learning rules can be used to capture the temporal relations between activation events involving pairs of neurons in artificial neural networks? Previous computational

research proposed various differential Hebbian learning (DHL) rules that rely on the activation of neurons and time derivatives of their activations to capture specific temporal relations between neural events. However, empirical research of brain plasticity, in particular plasticity depending on sequences of pairs of spikes involving the pre- and the post synaptic neurons, i.e., spike-timing-dependent plasticity (STDP), shows that the brain uses a surprisingly wide variety of different learning mechanisms that cannot be captured by the DHL rules proposed so far. Here we propose a general differential Hebbian learning (G-DHL) rule able to generate all existing DHL rules and many others. We show various examples of how the rule can be used to update the synapse in many different ways based on the temporal relation between neural events in pairs of artificial neurons. Moreover, we show the flexibility of the G-DHL rule by applying it to successfully fit several different STDP processes recorded in the brain. Overall, the G-DHL rule represents a new tool for conducting research on learning processes that depend on the timing of signal events.

S1 Supporting Information

This document presents the Supporting Information of the article: Zappacosta S., Mannella F., Mirolli M., Baldassarre G. (2018), *General Differential Hebbian Learning: Capturing Temporal Relations between Events in Neural Networks and the Brain*, Plos Computational Biology.

1 G-DHL and the systematisation of DHL rules

This section presents additional figures related to the Section ‘G-DHL and the systematisation of DHL’ in the main article.

1.1 Existing differential Hebbian learning rules

Fig. 1 shows the ‘dynamical’ continuous-time formulation of Hebb rule:

$$\dot{w} = (1/\tau) \cdot u_2 \cdot u_1 \tag{1}$$

where u_1 is the pre-synaptic neuron activation, u_2 is the post-synaptic neuron activation, and $\dot{w}$ is the instantaneous change of the connection weight.

Fig. 2 shows Kosko learning rule:

$$\dot{w} = (1/\tau) \cdot \dot{u}_2 \cdot \dot{u}_1 \tag{2}$$

Fig. 3 shows Porr and Wörgötter learning rule:

$$\dot{w} = (1/\tau) \cdot \dot{u}_2 \cdot u_1 \tag{3}$$

1.2 The three key elements for the computation of DHL rules and G-DHL: event, event derivative positive part, and event derivative negative part

An event is intended in the paper as a portion of the signal, lasting for a relatively short time, characterised by a *monotonically increasing value followed by a monotonically decreasing value*. Events are important for G-DHL because it uses the increasing part and the decreasing part of each event, captured through a first order derivative, to extract information on the temporal relation between the pre- and

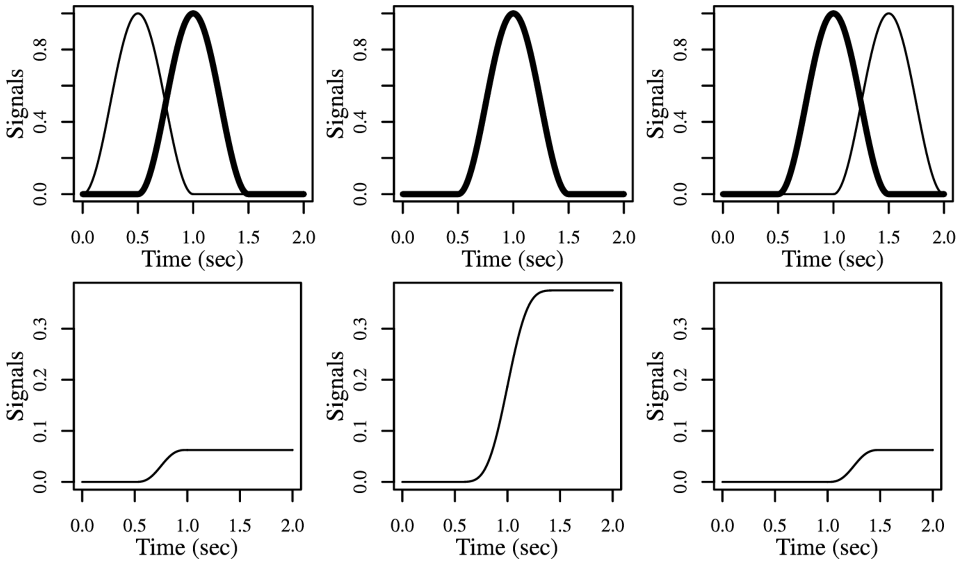

Figure 1. Hebb learning rule. Top: three examples of different possible temporal ordering of transient activation of two neurons. The non-zero activation part of the signal was obtained with a cosine function receiving input values ranging in $(-\pi, \pi)$: the output of this function was mapped onto $(0.5, 1.5)$ for the bold-curve signal, and onto $(0.0, 1.0)$, $(0.5, 1.5)$, and $(1.0, 2.0)$ for the thin-curve signal in respectively the three graphs. Bottom: weight update resulting from the application of Eq. 1 to the corresponding top graphs. The connection weight at a certain time T, given by $w(T) = \int_{-\infty}^{T} (1/\tau) u_2 u_1 \; dt$ in continuous time, was computed in discrete time as $\Sigma_{t=0}^{T} (dt/\tau) u_{2,t} u_{1,t}$ with $dt = 0.001$ and $\tau = 1$ ($t = 0$ could be used in place of $t = -\infty$ as in the example the weight does not change before $t = 0$).

post-synaptic events. Indeed, the increasing part of an event marks its *starting* early portion whereas its decreasing part marks its *ending* late portion, as shown in Fig. 4. The time overlap between the increasing and decreasing parts of the pre- and post-synaptic events thus allows the DHL rules to detect their temporal relation.

1.3 G-DHL: numerical equations and learning kernels with symmetric and asymmetric events

Fig. 5 shows the learning kernels produced by the eight G-DHL components using a symmetric event, in this case a cosine function. For completeness, the figure also shows the Hebb rule. Note how the learning kernels of some components have the same shape due to the symmetry in time of the cosine event considered (symmetry with respect to the vertical axis passing on the maximum of the cosine event) .

Fig. 6, presented in the main article but also reported here for ease of reference, shows all the learning kernels for the symmetric cosine events plotted together. Some of the kernels overlap due to the symmetry of the events considered, but they still form a set of basis functions that cover in a regular fashion the inter-event interval around the critical value of zero.

Fig. 7 shows the learning kernels of the G-DHL components using an asymmetric event given by an α-function. This figure, when compared with Fig. 5, shows what happens when the shape of the events is asymmetric in time. The first component, considering the derivative positive parts of both events, has now a spike-like shape because, contrary to cosine events, the α-function events start with a non-zero positive

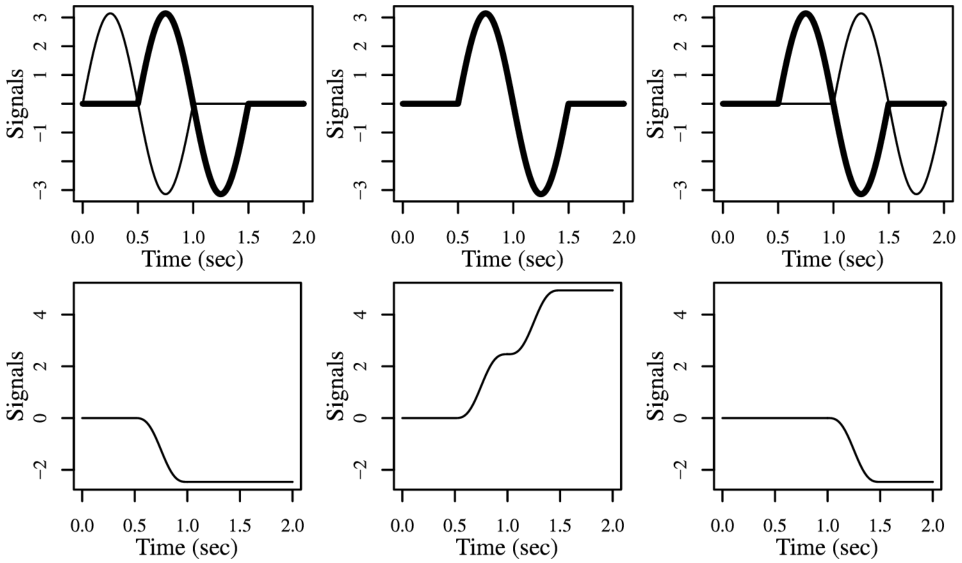

Figure 2. Kosko DHL rule. Top: first-order derivatives of the activation of two neurons (as those shown in Fig. 1, top) considered by the Kosko learning rule [1]. The step-by-step value of the derivatives, $\dot{u}_t$, was numerically computed at discrete times as $\dot{u}_t = (u_t - u_{t-1})/\Delta t$. Bottom: weight update resulting from the application of the Kosko learning rule of Eq. 2 on the basis of the derivatives shown in the corresponding top graphs. The connection weight at a certain time T was computed with the same procedure and parameters used for Fig. 1.

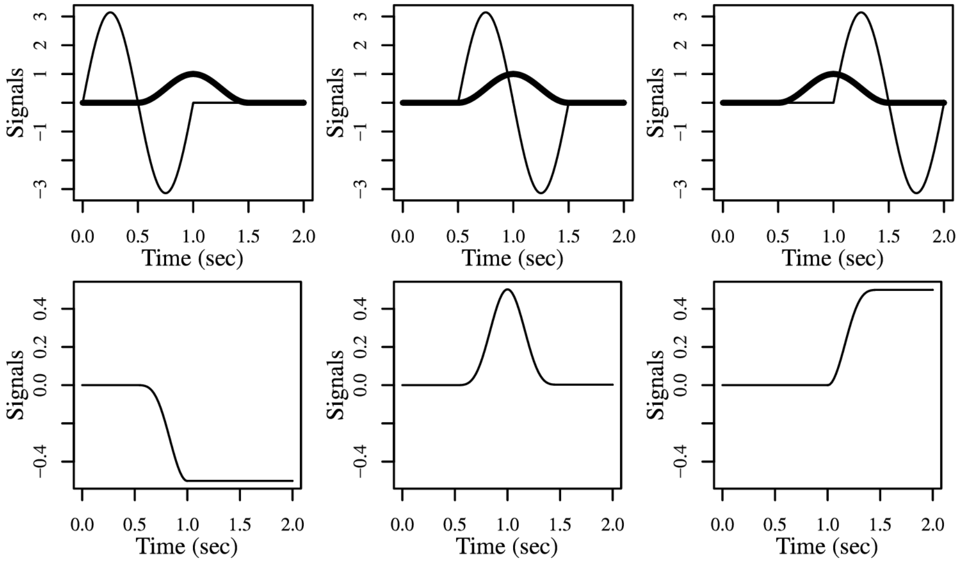

Figure 3. Porr-Wörgötter learning rule. Top: each graph shows the elements considered by the Porr-Wörgötter DHL rule [2], in particular the pre-synaptic neuron activation (bold curves) and the first derivative of the post-synaptic neuron activation (thin curves; the activation of both neurons was as the one in Fig. 1, top). Bottom: weight update resulting from the application of Porr-Wörgötter learning rule of Eq. 3 on the basis of the signals shown in the corresponding top graphs. The connection weight at a certain time T was computed with the same procedure and parameters used for Fig. 1.

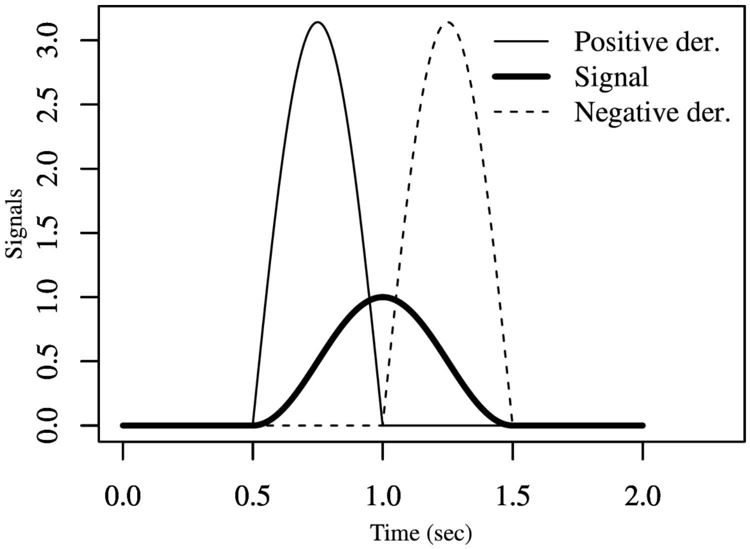

Figure 4. The three key elements used by the G-DHL rule: the event and its increasing and decreasing parts. An event involves a transient increase followed by a transient decrease of a neural signal in time. Here the event of the neural signal was generated with a cosine function as done in Fig. 1, starting at 0.5 sec and ending at 1.5 sec. The increasing part of the event, captured with the positive-part function of the neural signal derivative $[\dot{u}]^+$, starts at 0.5 sec and terminates at 1.0 sec. The decreasing part of the event, captured with the negative-part function $[\dot{u}]^-$, starts at 1.0 sec and terminates at 1.5 sec. Recall that the function $[.]^-$ changes the sign of its argument, so both the positive and negative parts of the derivative are always positive or null. Thus, when also the signal is positive (or null), as in many neural-network models, all the three elements are also positive (or null).

derivative.

Fig. 8 shows the same learning kernels for asymmetric events plotted in the same graph. The figure highlights how the kernels do not overlap and form a very regular set of learning basis functions that cover the inter-event time in a rather regular fashion around the zero value. With the α-function events, the Hebbian kernel overlaps (after rescaling) with the component based on the derivative negative parts of both events.

2 Methods: modelling STDP with G-DHL

This section presents the derivation of the explicit formulas presented in the Section 'Using G-DHL to fit STDP data sets' in the main article, related to the synaptic update resulting from the application of the eight components of the G-DHL rule to a pair of pre- and post-synaptic spikes. If the G-DHL rule is applied to events that have a tractable mathematical form, then it is possible to compute such explicit formulas. This section illustrates this by presenting the formulas that refer to a specific spike representation, the commonly used *Dirac δ-function*, and a specific eligibility trace formula, the *α-function* often used in the literature to represent the effect of a spike on the post-synaptic neuron membrane potential ('evoked post-synaptic spike potential' – EPSP). The formulas are computed for both the case where the τ coefficients of the pre- and post-synaptic neurons are different and for the case where they are the same. The section also reports the formulas for the

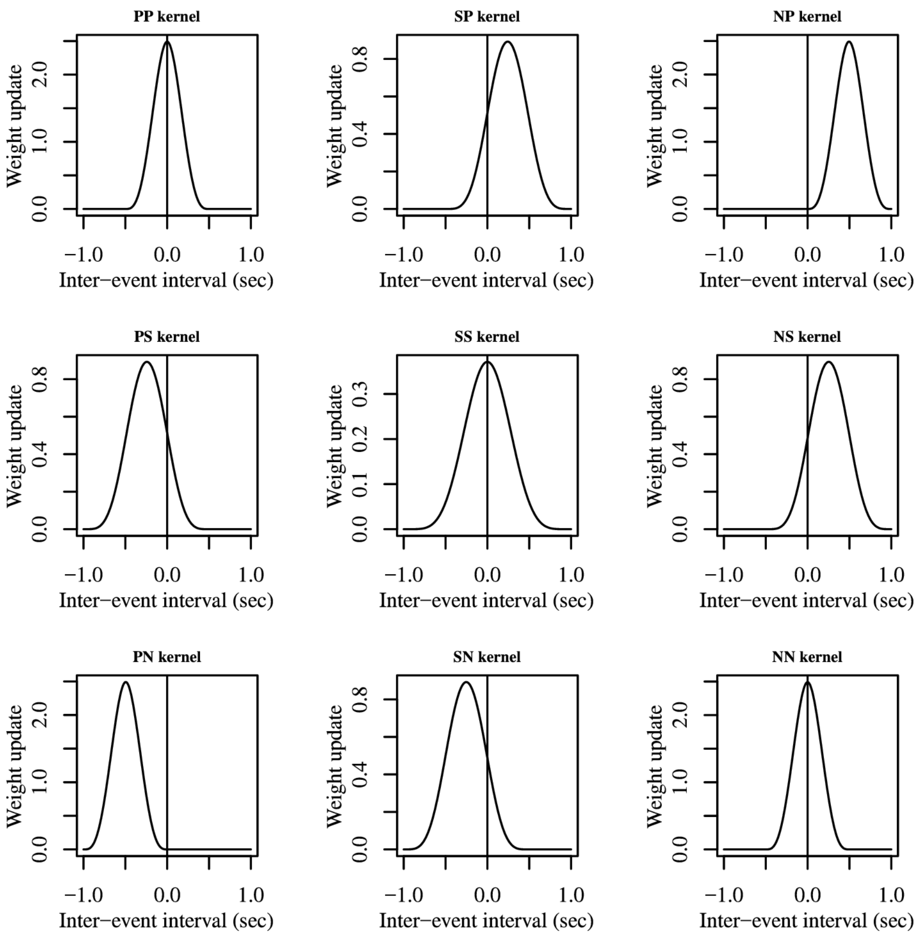

Figure 5. The learning kernels generated by the G-DHL rule components with symmetric events. The signals involved events generated with cosine functions as done in Fig. 1. The kernels are indicated with pairs of letters, for example 'PP', where the first letter refers to the pre-synaptic neuron and the second letter to the post-synaptic neuron. P stands for positive part of the first derivative of the neural signal; S stands for neural signal; and N stands for absolute value of the negative part of the first derivative of the neural signal. For completeness the figure also shows the learning kernel of the non-differential Hebb learning component (SS kernel).

computation of the Δt time interval that produces the maximum synaptic change, and the value of the latter. Finally, the section also reports the formulas for computing the integral of the learning kernels of the G-DHL rule components, indicating the overall tendency of the kernels to strengthen or weaken the synapse.

2.1 Computation of the explicit formulas of G-DHL applied to spikes

To apply the G-DHL rule to spiking neurons, we considered the spikes of the pre- and post-synaptic neurons as the target events causing the synaptic change. As often done in the literature [3, 4], we modelled a spike as a *Dirac δ-function* (in continuous time, this function can be conceived as the limit of an infinitely narrow function with an integral of 1, e.g. a standard normal function with a decreasing width; in discrete time, the function is implemented with a signal having a value of 1 for one specific

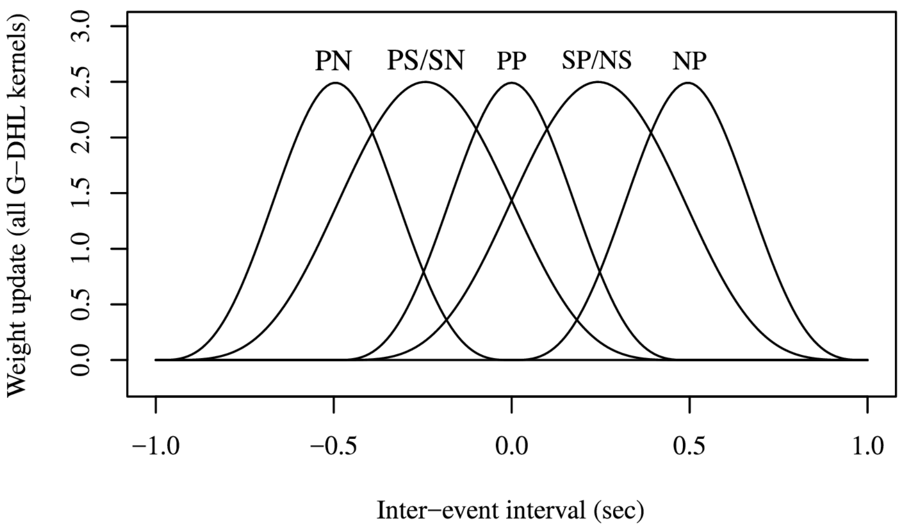

Figure 6. Superposition of learning kernels of the G-DHL rule applied to symmetric events. The learning kernels are those considered in Fig. 5.

time step and 0 otherwise [5]).

As we have seen, the G-DHL rule can capture time relations involving events only if these overlap in time. Voltage changes related to each neuron spike last few milliseconds, but empirical data on STDP show that the synaptic changes can be caused by pre- and post-synaptic spikes separated by several milliseconds. This implies that the first occurring spike must leave some electrical or chemical trace that overlaps with the second occurring spike or with a second trace depending on it.

Here we modelled each trace with an α-*function* as this is commonly used to capture the excitatory post-synaptic potentials (EPSP) evoked by a pre-synaptic spike [3, 4]. We also used it to mimic possible electrochemical events that might be caused by the post-synaptic spike, for example the back-propagating action potential (BAP) affecting the post-synaptic neuron [6]. The α-function actually represents a family of functions of the type $x \cdot exp(-x)$. Here we employed a commonly used form of the function based on a double application of the leaky integrator filter: (a) a first application creates the trace in time and forms the descending part of the event after the spike; (b) a second application creates the increasing part of the event. The two filters are mathematically expressed as follows:

$$\tau_a \dot{v}(t) = -v(t) + \kappa \; s(t) \; , \qquad \tau_b \dot{u}(t) = -u(t) + v(t) \; , \tag{4}$$

where $s(t)$ is the initial signal containing the spike, $v(t)$ is the output of the first filter, $u(t)$ is the output of the second filter, τ_a and τ_b are time constants, and κ is an amplification parameter. If we assume that the two filters have the same time constants ($\tau_a = \tau_b = \tau$) then they can be combined to obtain a single filter (this is done by computing $v(t)$ from the second equation and by substituting it in the first one):

$$\tau^2 \ddot{u}(t) = -2\tau \dot{u}(t) - u(t) + \kappa \, s(t) \; . \tag{5}$$

Assuming different decay constants of the two leaky integrator filters allow the independent regulation of the duration of the ascending and descending part of the event. This might be useful in some applications but is not further investigated here where we assume a unique τ for each neuron. However, we will assume a possibly different τ for the pre- and post- neurons as this allows the regulation of the temporal

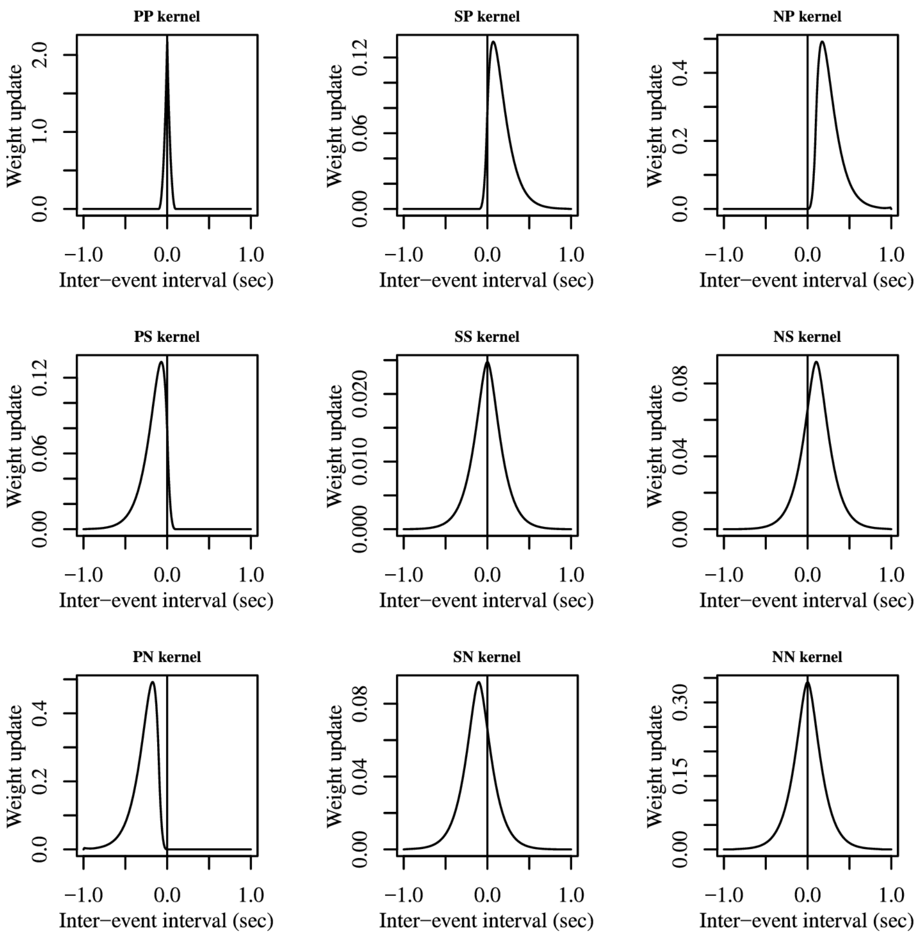

Figure 7. The learning kernels generated by the G-DHL rule components with non-symmetric events. The signals and kernels were computed as in Fig. 1 but they involved events that were formed by an α-function with equation $u = 10 \cdot t \cdot exp(-t/0.1)$.

window over which the G-DHL components operate: this is important as such parameters can be automatically estimated when fitting real data on STDP. The parameter κ (also possibly estimated in an automatic fashion) allows the amplification of the output of the filter as the leaky integrator filters tend to produce a signal with very low amplitude.

The formula used to form the events determines if the computation of the G-DHL synaptic update can be computed only numerically or both numerically and analytically. If the formula has a tractable mathematical form then we can compute the analytic form of the synaptic change produced by the G-DHL rule components. The form is tractable when it allows the computation of the finite integrals, and their ranges, generated by the application of the G-DHL components. We now show this by obtaining the formula of the G-DHL synaptic update when the α-function (Eq. 5) is used to generate the eligibility traces (events) caused by the pre- and post-synaptic spikes.

The pre- and post-synaptic spikes are assumed to take place at times t_i, with $i = 1, 2$, so they are separated by a time delay $\Delta t = t_2 - t_1$ (note how a positive Δt implies that the pre-synaptic spike occurs before the post-synaptic one). The solution of the second order nonhomogeneous differential equation of the trace (Eq. 5) for each

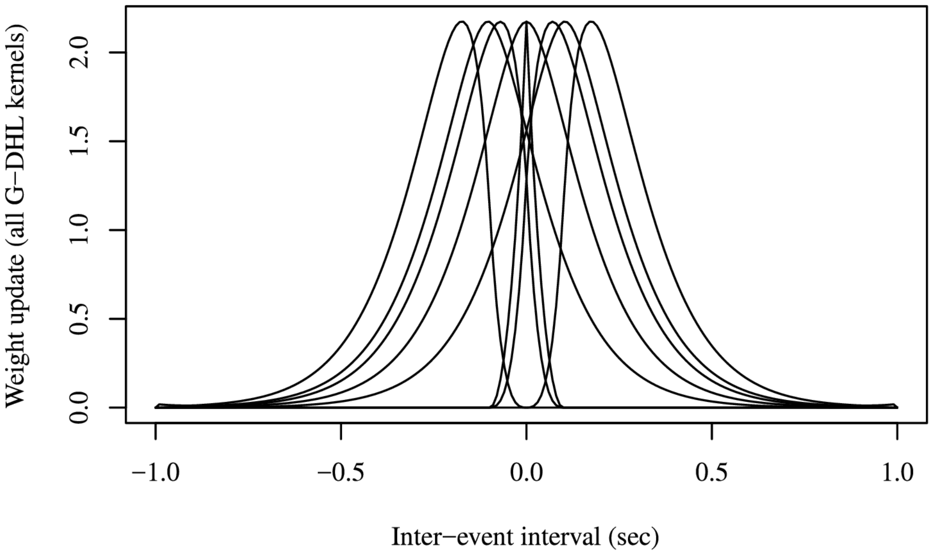

Figure 8. Superposition of learning kernels of the G-DHL rule appied to non-symmetric events. The learning kernels are those considered in Fig. 7.

of the two Dirac δ-function spikes gives the following explicit formula, expressing the spike-related events on which the G-DHL rule is applied:

$$u_i(t) = \begin{cases} \kappa \frac{t-t_i}{\tau_i} \mathrm{e}^{-\frac{t-t_i}{\tau_i}} & \text{if } t \geq t_i \\ 0 & \text{if } t < t_i \end{cases} \tag{6}$$

where the constant τ_i differs for the two neurons i ($i = 1, 2$). Note that in this formula the index i of u_i refers to both parameters τ_i and t_i on which u_i depends. To avoid confusion, also notice that for the first neuron $\tau_a = \tau_b = \tau_1$ and for the second neuron $\tau_a = \tau_b = \tau_2$.

Fig. 9a shows a neuron activation with one spike and the signals returned by applying the two leaky integral filters to it. The event signal has its maximum, equal to $\kappa\mathrm{e}^{-1}$, at the time step $t_i + \tau_i$. Below we show how this time step, at which the event stops increasing and starts decreasing, is important for the computation of the definite integrals needed for the analytic computation of the G-DHL synaptic update.

The G-DHL rule detects the increasing and decreasing parts of the signals u_i on the basis of the positive part $[\dot{u}_i]^+$ and negative part $[\dot{u}_i]^-$ of their derivative signals (Fig. 9b). The derivative applied to the event signal of Eq. (6) is:

$$\dot{u}_i(t) = \begin{cases} \frac{\kappa}{\tau_i} \mathrm{e}^{-\frac{t-t_i}{\tau_i}} \left(1 - \frac{t-t_i}{\tau_i}\right) & \text{if } t \geq t_i \\ 0 & \text{if } t < t_i \ . \end{cases} \tag{7}$$

We now compute the explicit formulas of the synaptic changes caused by the different G-DHL rule components as a function of the time Δt between the spikes, if one considers events as in Eq. (6) (see Fig. 10). What is relevant for applying the G-DHL rule is the time relation between the two events, namely $\Delta t = t_2 - t_1$, and not the absolute values of t_1 and t_2, so in the following we assume $t_1 = 0$ and $t_2 = \Delta t$ without loss of generality. The synaptic change can be obtained integrating, over the time of one trial, the product forming each G-DHL component. For each component, the definite integrals of such product, covering different portion of the time line, depend on the overlaps in time of the event signals or the positive/negative part of the

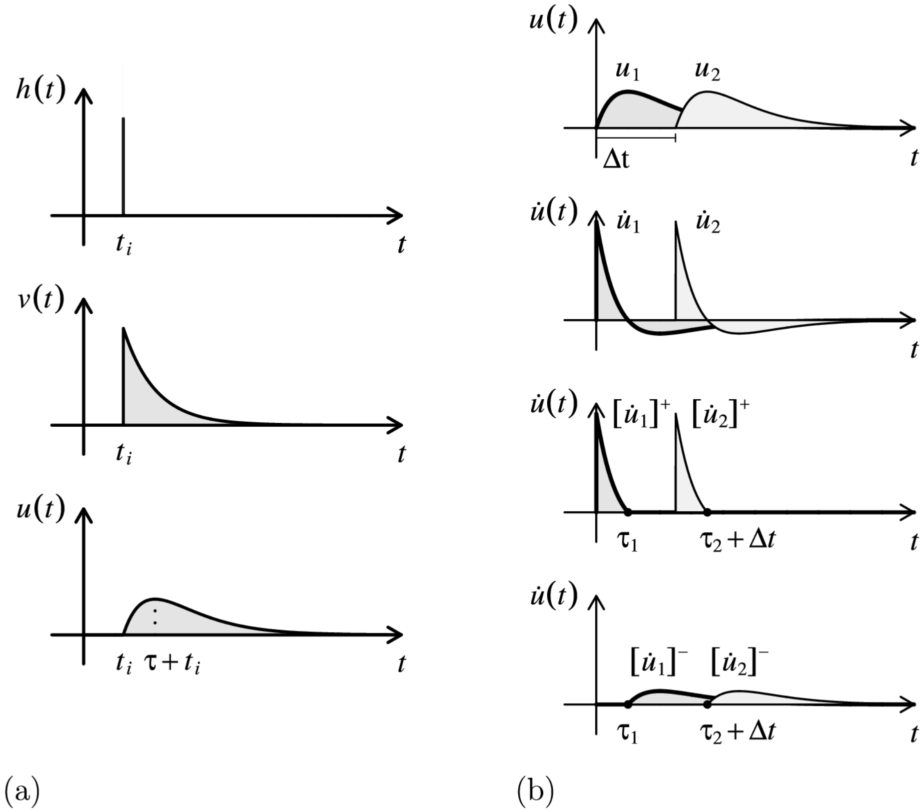

Figure 9. Spike, α-function, events, and their derivative positive/negative parts. (a) From top to bottom: a spike signal $h(t)$; the output $v(t)$ of a first leaky accumulator filter applied to the spike signal; the output $u(t)$ of a second leaky accumulator filter applied to $v(t)$. (b) From top to bottom: example of two event pre-synaptic and post-synaptic signals $u_i(t)$ characterised by $\tau_1 = \tau_2$ and obtained, as done in 'a', from two spikes happening respectively at $t_1 = 0$ and $t_2 = \Delta t$; their derivatives $\dot{u}_i(t)$; and the positive parts $[\dot{u}_i]^+$ and negative parts $[\dot{u}_i]^-$ of such derivatives needed to compute the G-DHL rule components.

event signal derivatives of the two neurons. Such overlaps can be defined on the basis of τ_1, τ_2 and Δt. Indeed, the derivative signal of each event is divided into the positive and a negative part by the time of the maximum of the event signal, corresponding to respectively τ_1 and $\tau_2 + \Delta t$ for the two neurons. Below we consider the case $\tau_1 \geq \tau_2$ as the case $\tau_1 < \tau_2$ involves similar computations. Moreover we assume that u_i with the smaller τ_i starts at $t_i = 0$, and u_i with the larger τ_i starts at $t_i = \Delta t$. The integrals related to all other possible conditions are computed in a similar way.

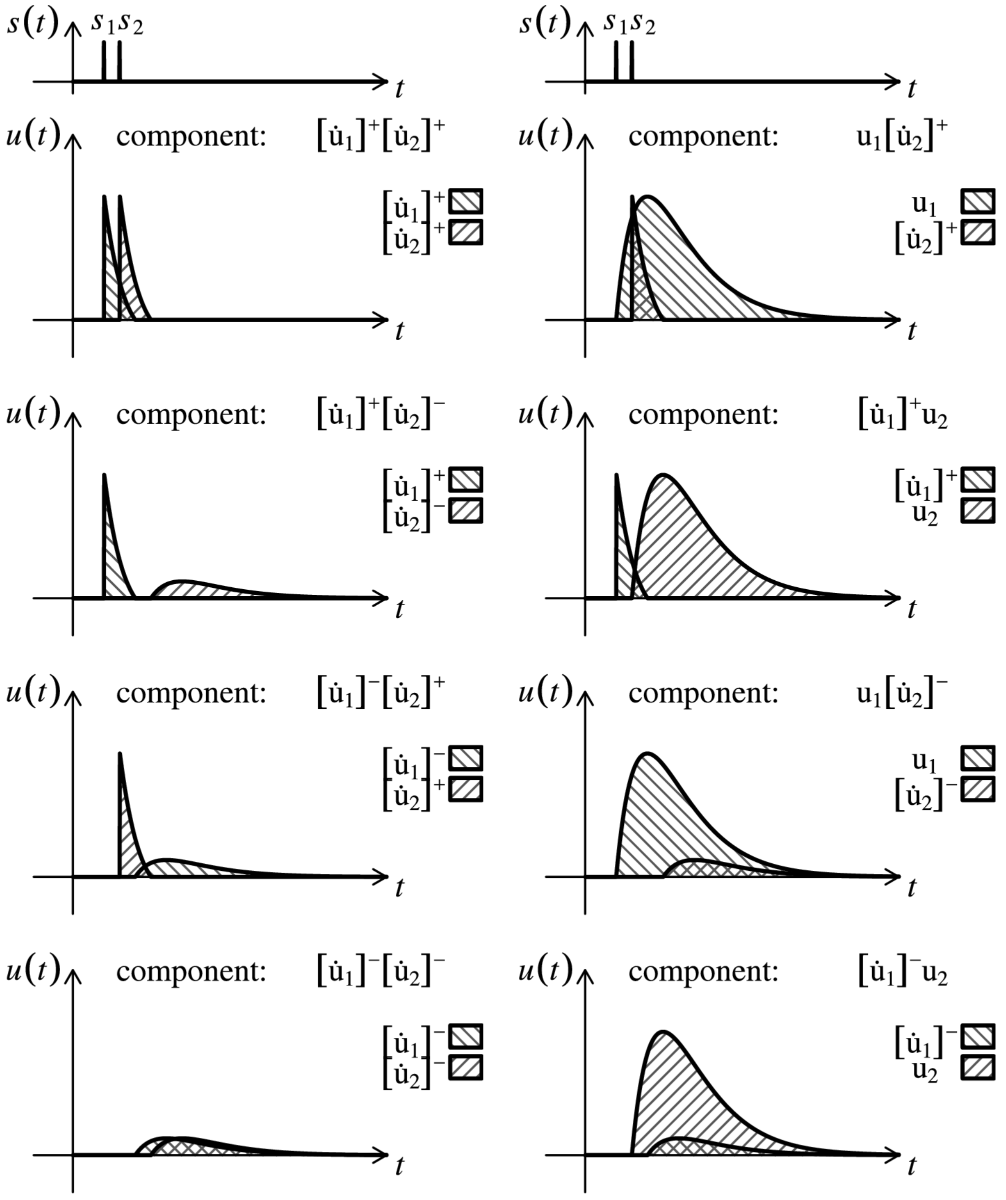

(a) (b)

Figure 10. Elements of the computation of the G-DHL components related to two spiking signals. In this example, the two spike signals s_1 and s_2 happen at respectively t_1 and $t_2 = t_1 + \Delta t$ and have $\tau_1 = \tau_2$. (a) Differential components of the G-DHL rule. The positive and negative parts of the event derivatives (Eq. (7)) used by each component are highlighted with lined areas. Recall that $[.]^-$ returns positive values when applied to the negative values of the derivatives. (b) Mixed components of the rule. The event (Eq. (6)) and the positive/negative parts of the event derivatives (Eq. (7)) used by each component are highlighted with lined areas. Notice how the different elements of the components can overlap in time, leading to a synaptic update, depending on Δt, τ_1, and $\tau_2 + \Delta t$.

The definite integrals of the four G-DHL differential components are hence:

$$\Delta w_{pp}(\Delta t) = \begin{cases} \int_0^{\tau_2+\Delta t} \dot{u}_1(s)\dot{u}_2(s)\,\mathrm{d}s & \text{if } -\tau_2 < \Delta t \leq 0 \\ \int_{\Delta t}^{\tau_2+\Delta t} \dot{u}_1(s)\dot{u}_2(s)\,\mathrm{d}s & \text{if } 0 < \Delta t \leq \tau_1 - \tau_2 \\ \int_{\Delta t}^{\tau_1} \dot{u}_1(s)\dot{u}_2(s)\,\mathrm{d}s & \text{if } \tau_1 - \tau_2 < \Delta t \leq \tau_1 \\ 0 & \text{elsewhere} \end{cases} \tag{8}$$

$$\Delta w_{nn}(\Delta t) = \begin{cases} \int_{\tau_1}^{\infty} \dot{u}_1(s)\dot{u}_2(s)\,\mathrm{d}s & \text{if } \Delta t \leq \tau_1 - \tau_2 \\ \int_{\Delta t+\tau_2}^{\infty} \dot{u}_1(s)\dot{u}_2(s)\,\mathrm{d}s & \text{if } \Delta t > \tau_1 - \tau_2 \end{cases} \tag{9}$$

$$\Delta w_{np}(\Delta t) = \begin{cases} -\int_{\tau_1}^{\Delta t+\tau_2} \dot{u}_1(s)\dot{u}_2(s)\,\mathrm{d}s & \text{if } \tau_1 - \tau_2 < \Delta t \leq \tau_1 \\ -\int_{\Delta t}^{\Delta t+\tau_2} \dot{u}_1(s)\dot{u}_2(s)\,\mathrm{d}s & \text{if } \Delta t > \tau_1 \\ 0 & \text{elsewhere} \end{cases} \tag{10}$$

$$\Delta w_{pn}(\Delta t) = \begin{cases} -\int_0^{\tau_1} \dot{u}_1(s)\dot{u}_2(s)\,\mathrm{d}s & \text{if } \Delta t \leq -\tau_2 \\ -\int_{\Delta t+\tau_2}^{\tau_1} \dot{u}_1(s)\dot{u}_2(s)\,\mathrm{d}s & \text{if } -\tau_2 < \Delta t \leq \tau_1 - \tau_2 \\ 0 & \text{elsewhere ,} \end{cases} \tag{11}$$

where $\Delta w_{pp}(\Delta t)$, $\Delta w_{nn}(\Delta t)$, $\Delta w_{np}(\Delta t)$, and $\Delta w_{pn}(\Delta t)$ are the synaptic changes due to the four components, with 'p' and 'n' indicating respectively the positive and negative parts of the onset-signal derivatives.

The integrals of the four G-DHL mixed components are as follows:

$$\Delta w_{sp}(\Delta t) = \begin{cases} \int_0^{\tau_2+\Delta t} u_1(s)\dot{u}_2(s)\,\mathrm{d}s & \text{if } -\tau_2 < \Delta t \leq 0 \\ \int_{\Delta t}^{\tau_2+\Delta t} u_1(s)\dot{u}_2(s)\,\mathrm{d}s & \text{if } \Delta t > 0 \\ 0 & \text{elsewhere} \end{cases} \tag{12}$$

$$\Delta w_{ps}(\Delta t) = \begin{cases} \int_0^{\tau_1} \dot{u}_1(s)u_2(s)\,\mathrm{d}s & \text{if } \Delta t \leq 0 \\ \int_{\Delta t}^{\tau_1} \dot{u}_1(s)u_2(s)\,\mathrm{d}s & \text{if } 0 < \Delta t \leq \tau_1 \\ 0 & \text{elsewhere} \end{cases} \tag{13}$$

$$\Delta w_{sn}(\Delta t) = \begin{cases} -\int_0^{\infty} u_1(s)\dot{u}_2(s)\,\mathrm{d}s & \text{if } \Delta t \leq -\tau_2 \\ -\int_{\Delta t+\tau_2}^{\infty} u_1(s)\dot{u}_2(s)\,\mathrm{d}s & \text{if } \Delta t > -\tau_2 \\ 0 & \text{elsewhere} \end{cases} \tag{14}$$

$$\Delta w_{ns}(\Delta t) = \begin{cases} -\int_{\tau_1}^{\infty} \dot{u}_1(s)u_2(s)\,\mathrm{d}s & \text{if } \Delta t \leq \tau_1 \\ -\int_{\Delta t}^{\infty} \dot{u}_1(s)u_2(s)\,\mathrm{d}s & \text{if } \Delta t > \tau_1 \\ 0 & \text{elsewhere ,} \end{cases} \tag{15}$$

where $\Delta w_{sp}(\Delta t)$, $\Delta w_{ps}(\Delta t)$, $\Delta w_{sn}(\Delta t)$, and $\Delta w_{ns}(\Delta t)$ are the synaptic changes due to the four mixed components, with 's' indicating the onset signals.

2.2 Explicit equations of the G-DHL components when $\tau_1 \neq \tau_2$

The G-DHL rule presented in the main article is a function that returns the instant synaptic change based on the value of the input signals and the positive and negative parts of their derivatives. Eq. (8)–(11) and (12)–15 return the synaptic changes caused by each component of the rule on the basis of the definite integral of the component computed over $t \in (-\infty, +\infty)$. In turn, the definite integral of each component is a sum of proper and improper integrals depending on the overlaps of the events and their positive/negative derivative parts. The explicit formulas of these proper/improper integrals have been computed with *Maxima*, an open-source computer algebra system. These formula can be used to directly compute the synaptic change caused by the

different components given the parameters Δt, τ_1, τ_2, and κ. The G-DHL rule then linearly combines such values on the basis of the σ and η parameters.

Given the following short notations:

$$\rho = \tau_2/\tau_1 \qquad \sigma_1 = e^{\frac{\Delta t}{\rho\,\tau_1}+\frac{\Delta t}{\tau_1}+\rho+1} \qquad \sigma_2 = e^{\rho+1}$$
$$\sigma_3 = e^{\frac{\Delta t}{\rho\,\tau_1}+\frac{\Delta t}{\tau_1}} \qquad \sigma_4 = e^{\frac{1}{\rho}+1} \qquad \sigma_5 = e^{\frac{\Delta t}{\rho\,\tau_1}+\frac{\Delta t}{\tau_1}+\rho}$$
$$\sigma_6 = e^{\frac{1}{\rho}} \qquad \sigma_7 = e^{\frac{-\tau_1\left(\rho^2+\rho+1\right)-\Delta t\,\rho}{\tau_1\,\rho}} \qquad \sigma_8 = e^{\frac{-\tau_1\left(\rho^2+\rho+1\right)-\Delta t\,\rho}{\tau_1\,\rho}}$$

the explicit formulas related to the differential components of the G-DHL rule are as follows:

$$\Delta w_{pp}(\Delta t) = \begin{cases} -\dfrac{\kappa^2\left(\tau_1\,\rho\left(\rho^2-2\,\sigma_1\,\rho+2\,\rho-1\right)+\Delta t\,(\rho+1)\,(\rho-\sigma_1)\right)e^{-\frac{\tau_1\,(\rho+1)+\Delta t}{\tau_1}}}{\tau_1^2\,\rho\,(\rho+1)^3} & \text{if } -\tau_2 < \Delta t \le 0 \\ -\dfrac{\kappa^2\left(\tau_1\left(\rho^2-2\,\sigma_2\,\rho+2\,\rho-1\right)+\Delta t\,(\rho+1)\,(\sigma_2\,\rho+1)\right)e^{-\frac{\tau_1\,(\rho+1)+\Delta t}{\tau_1}}}{\tau_1^2\,(\rho+1)^3} & \text{if } 0 < \Delta t \le \tau_1 - \rho\tau_1 \\ -\dfrac{\kappa^2\left(\Delta t\,(\rho+1)\,(\sigma_4\,\rho-\sigma_3)-\tau_1\left(\sigma_3\,\rho^2+2\,\sigma_4\,\rho-2\,\sigma_3\,\rho-\sigma_3\right)\right)e^{-\frac{\tau_1\,(\rho+1)+\Delta t\,\rho}{\tau_1\,\rho}}}{\tau_1^2\,(\rho+1)^3} & \text{if } \tau_1-\rho\tau_1 < \Delta t \le \tau_1 \\ 0 & \text{elsewhere} \end{cases} \tag{16}$$

$$\Delta w_{nn}(\Delta t) = \begin{cases} -\dfrac{\kappa^2\left(\tau_1\,\rho^2-\tau_1\,(2\,\rho+1)+\Delta t\,\rho+\Delta t\right)e^{\frac{\Delta t}{\tau_1\,\rho}-\frac{1}{\rho}-1}}{\tau_1^2\,(\rho+1)^3} & \text{if } \Delta t \le \tau_1-\rho\tau_1 \\ \dfrac{\kappa^2\left(\tau_1\,\rho^2+\tau_1\,(2\,\rho-1)+\Delta t\,\rho+\Delta t\right)e^{-\rho-\frac{\Delta t}{\tau_1}-1}}{\tau_1^2\,(\rho+1)^3} & \text{if } \Delta t > \tau_1-\rho\tau_1 \end{cases} \tag{17}$$

$$\Delta w_{np}(\Delta t) = \begin{cases} \dfrac{\sigma_7\,\kappa^2\left(\tau_1\left(\sigma_6\,\rho^2+\sigma_5\,\rho^2+2\,\sigma_6\,\rho-2\,\sigma_5\,\rho-\sigma_6-\sigma_5\right)+(\sigma_6+\sigma_5)\,\Delta t\,(\rho+1)\right)}{\tau_1^2\,(\rho+1)^3} & \text{if } \tau_1-\rho\tau_1 < \Delta t \le \tau_1 \\ \dfrac{\kappa^2\left(\tau_1\left(\rho^2-2\,\sigma_2\,\rho+2\,\rho-1\right)+\Delta t\,(\rho+1)\,(\sigma_2\,\rho+1)\right)e^{-\frac{\tau_1\,(\rho+1)+\Delta t}{\tau_1}}}{\tau_1^2\,(\rho+1)^3} & \text{if } \Delta t > \tau_1 \\ 0 & \text{elsewhere} \end{cases} \tag{18}$$

$$\Delta w_{pn}(\Delta t) = \begin{cases} -\dfrac{\kappa^2\left(\tau_1\,\rho\left(\rho^2+2\,\sigma_4\,\rho-2\,\rho-1\right)+\Delta t\,(\rho+1)\,(\rho+\sigma_4)\right)e^{-\frac{\tau_1\,(\rho+1)-\Delta t}{\tau_1\,\rho}}}{\tau_1^2\,\rho\,(\rho+1)^3} & \text{if } \Delta t \le -\rho\tau_1 \\ -\dfrac{\sigma_8\,\kappa^2\left(\tau_1\left(\sigma_6\,\rho^2+\sigma_5\,\rho^2+2\,\sigma_6\,\rho-2\,\sigma_5\,\rho-\sigma_6-\sigma_5\right)+(\sigma_6+\sigma_5)\,\Delta t\,(\rho+1)\right)}{\tau_1^2\,(\rho+1)^3} & \text{if } -\rho\tau_1 < \Delta t \le \tau_1-\rho\tau_1 \\ 0 & \text{elsewhere} \end{cases} \tag{19}$$

The synaptic changes resulting from these integrals are plotted in a figure in the main article.

The explicit equations related to the mixed components of the G-DHL rule are as follows:

$$\Delta w_{sp}(\Delta t) = \begin{cases} \dfrac{\kappa^2\,(\rho\,(\tau_1\,(\rho-\sigma_1+3)+\sigma_1\,\tau_1\,\rho+\sigma_1\,\Delta t+\Delta t)+(\sigma_1+1)\,\Delta t)\,e^{-\rho-\frac{\Delta t}{\tau_1}-1}}{\tau_1\,(\rho+1)^3} & \text{if } -\rho\tau_1 < \Delta t \le 0 \\ \dfrac{\kappa^2\,(\rho\,(\tau_1\,(\rho-\sigma_2+3)+\sigma_2\,\Delta t\,\rho+\tau_1\,\sigma_2\,\rho+\sigma_2\,\Delta t+\Delta t)+\Delta t)\,e^{-\rho-\frac{\Delta t}{\tau_1}-1}}{\tau_1\,(\rho+1)^3} & \text{if} \Delta t > 0 \\ 0 & \text{elsewhere} \end{cases} \tag{20}$$

$$\Delta w_{ps}(\Delta t) = \begin{cases} -\dfrac{\kappa^2\,(\rho\,(-\tau_1\,(3\,\rho+\sigma_4+1)+\Delta t\,\rho+\tau_1\,\sigma_4\,\rho+\sigma_4\,\Delta t+\Delta t)+\sigma_4\,\Delta t)\,e^{\frac{\Delta t}{\tau_1\,\rho}-\frac{1}{\rho}-1}}{\tau_1\,(\rho+1)^3} & \text{if} \Delta t \le 0 \\ -\dfrac{\kappa^2\,\rho\,(\tau_1\,(\sigma_4\,\rho-3\,\sigma_3\,\rho-\sigma_4-\sigma_3)+(\sigma_4+\sigma_3)\,\Delta t\,(\rho+1))\,e^{-\frac{\tau_1\,(\rho+1)+\Delta t\,\rho}{\tau_1\,\rho}}}{\tau_1\,(\rho+1)^3} & \text{if } 0 < \Delta t \le \tau_1 \\ 0 & \text{elsewhere} \end{cases} \tag{21}$$

$$\Delta w_{sn}(\Delta t) = \begin{cases} -\dfrac{\kappa^2\,((\tau_1\,(\rho-1)+\Delta t)\,\rho+\Delta t)\,e^{\frac{\Delta t}{\tau_1\,\rho}}}{\tau_1\,(\rho+1)^3} & \text{if } \Delta t \le -\rho\tau_1 \\ \dfrac{\kappa^2\,(\rho\,(\tau_1\,(\rho+3)+\Delta t)+\Delta t)\,e^{-\rho-\frac{\Delta t}{\tau_1}-1}}{\tau_1\,(\rho+1)^3} & \text{if } \Delta t > -\rho\tau_1 \end{cases} \tag{22}$$

$$\Delta w_{ns}(\Delta t) = \begin{cases} -\dfrac{\kappa^2\,\rho\,(\Delta t\,(\rho+1)-\tau_1\,(3\,\rho+1))\,e^{\frac{\Delta t}{\tau_1\,\rho}-\frac{1}{\rho}-1}}{\tau_1\,(\rho+1)^3} & \text{if } \Delta t \le \tau_1 \\ \dfrac{e^{-\frac{\Delta t}{\tau_1}}\,\kappa^2\,\rho\,(\Delta t\,(\rho+1)+\tau_1\,(\rho-1))}{\tau_1\,(\rho+1)^3} & \text{if } \Delta t > \tau_1 \end{cases} \tag{23}$$

The synaptic changes resulting from these integrals are plotted in a figure in the main article.

2.3 Maxima of the synaptic change

The components of the G-DHL rule have different learning kernels. For each kernel it is thus interesting to know the maximum synaptic change, and the Δt that causes it, in relation to the parameters of the α-function of the pre- and post-synaptic neurons (τ_1, τ_2, κ). This knowledge can be used for various purposes, for example to tune by hand the desired synaptic change in computational models using the formula.

The pair of values M, respectively indicating the maximum of the kernel and the corresponding Δt, are as follows for the differential components (the formulas use the short notations used in the previous section):

$$M_{pp} = \left(-\frac{\kappa^2\,(\rho\,(\rho-2\,\sigma_2+2)-1)}{\tau_1\,\sigma_2\,(\rho+1)^3}\right) \tag{24}$$

$$M_{nn} = \left(\frac{\kappa^2\,e^{-\frac{2}{\rho+1}-1}}{\tau_1\,(\rho+1)^2}\right) \tag{25}$$

$$M_{np} = \left(\frac{\kappa^2\,(\sigma_2\,\rho+1)\,e^{-\frac{\sigma_2\,\rho\,\left(\rho^2+3\,\rho+4\right)+\rho+3}{(\rho+1)\,(\sigma_2\,\rho+1)}}}{\tau_1\,(\rho+1)^2}\right) \tag{26}$$

$$M_{pn} = \left(\frac{\kappa^2\,(\rho+\sigma_4)\,e^{-\frac{\sigma_4\,\left(4\,\rho^2+3\,\rho+1\right)+\rho^2\,(3\,\rho+1)}{\rho\,(\rho+1)\,(\rho+\sigma_4)}}}{\tau_1\,(\rho+1)^2}\right) \tag{27}$$

Notice how the kernel maximum values change for different τ_1 if the ratio ρ between τ_1 and τ_2 is kept constant. In this case, the formulas show that all maximum values have τ_1 at the denominator, so the maximum synaptic change decreases with the increase of τ_1 and τ_2.

Regarding the values of the Δt that produce the maximum values of the components, it is important to notice when they are positive, zero, or negative as this marks the LTP/LTD nature of the component. Thus: (a) for M_{pp}: the maximum is always on $\Delta t = 0$; (b) for M_{nn}: the maximum is at the left of $\Delta t = 0$ for $\tau_1 < \tau_2$, on it for $\tau_1 = \tau_2$, and on its right for $\tau_1 > \tau_2$; (c) for M_{np}: the maximum is always on the positive semi-axis of Δt and when τ_1 gets larger it decreases with an inverse proportionality while Δt increases with a direct proportionality: this implies that the point follows a hyperbolic curve; (d) for M_{pn}: the maximum is always on the negative semi-axis of Δt and when τ_1 gets larger it decreases with an inverse proportionality together with Δt: this implies that the point follows a hyperbolic curve.

For the mixed components of the G-DHL rule, the maximum values and the related Δt are as follows:

$$M_{sp} = \left(\frac{\kappa^2\,(\sigma_2\,\rho+1)\,e^{-\frac{\sigma_2\,\rho\,\left(\rho^2+2\,\rho+3\right)+2}{(\rho+1)\,(\sigma_2\,\rho+1)}}}{(\rho+1)^2}\right) \tag{28}$$

$$M_{ps} = \left(\frac{\kappa^2\,\rho\,(\rho+\sigma_4)\,e^{-\frac{2\,\rho^3+\sigma_4\,\left(3\,\rho^2+2\,\rho+1\right)}{\rho\,(\rho+1)\,(\rho+\sigma_4)}}}{(\rho+1)^2}\right) \tag{29}$$

$$M_{sn} = \left(\frac{\kappa^2\,e^{-\frac{2}{\rho+1}}}{(\rho+1)^2}\right) \tag{30}$$

$$M_{ns} = \left(\frac{\kappa^2\,\rho\,e^{-\frac{2}{\rho+1}}}{(\rho+1)^2}\right) \tag{31}$$

In this case the formulas show that the maximum value does not depend on the value of a specific time constant, but only on their ratio ρ: if this ratio is kept constant

the maximum does not change. Instead, the Δt corresponding to the maximum changes as follows for an increasing τ_1: (a) M_{sp}: the value shifts to the right; (b) M_{ps}: the value shifts to the left; (c) M_{sn}: the value shifts to the left; (d) M_{ns}: the value shifts to the right;

2.4 Synaptic change when $\tau_1 = \tau_2$

In the case of equal time constants of the neurons, $\tau_1 = \tau_2 = \tau$ (hence $\rho = 1$), the formulas for the synapse update, illustrated in Section 2.2, become much simpler:

$$\Delta w_{pp}(\Delta t) = \begin{cases} \kappa^2 \mathrm{e}^{\frac{\Delta t}{\tau}} \frac{\mathrm{e}^2 - \mathrm{e}^{-\frac{\Delta t}{\tau}}}{4\mathrm{e}^2\tau^2}(\tau + \Delta t) & \text{if } -\tau < \Delta t \leq 0 \\ \kappa^2 \mathrm{e}^{-\frac{\Delta t}{\tau}} \frac{\mathrm{e}^2 - \mathrm{e}^{\frac{\Delta t}{\tau}}}{4\mathrm{e}^2\tau^2}(\tau - \Delta t) & \text{if } 0 < \Delta t \leq \tau \\ 0 & \text{elsewhere} \end{cases} \tag{32}$$

$$\Delta w_{pn}(\Delta t) = \begin{cases} \kappa^2 \mathrm{e}^{\frac{\Delta t}{\tau}} \frac{-\Delta t - \mathrm{e}^2\Delta t + \tau - \mathrm{e}^2\tau}{4\mathrm{e}^2\tau^2} & \text{if } \Delta t \leq -\tau \\ \kappa^2 \frac{-\Delta t \cosh\frac{\Delta t}{\tau} + \tau \sinh\frac{\Delta t}{\tau}}{2\mathrm{e}^2\tau^2} & \text{if } -\tau < \Delta t \leq 0 \\ 0 & \text{if } \Delta t > 0 \end{cases} \tag{33}$$

$$\Delta w_{np}(\Delta t) = \begin{cases} 0 & \text{if } \Delta t \leq 0 \\ \kappa^2 \frac{\Delta t \cosh\frac{\Delta t}{\tau} - \tau \sinh\frac{\Delta t}{\tau}}{2\mathrm{e}^2\tau^2} & \text{if } 0 < \Delta t \leq \tau \\ \kappa^2 \mathrm{e}^{-\frac{\Delta t}{\tau}} \frac{\Delta t + \mathrm{e}^2\Delta t + \tau - \mathrm{e}^2\tau}{4\mathrm{e}^2\tau^2} & \text{if } \Delta t > \tau \end{cases} \tag{34}$$

$$\Delta w_{nn}(\Delta t) = \begin{cases} \kappa^2 \mathrm{e}^{\frac{\Delta t}{\tau}} \frac{\tau - \Delta t}{4\mathrm{e}^2\tau^2} & \text{if } \Delta t \leq 0 \\ \kappa^2 \mathrm{e}^{-\frac{\Delta t}{\tau}} \frac{\tau + \Delta t}{4\mathrm{e}^2\tau^2} & \text{if } \Delta t > 0 \end{cases} \tag{35}$$

$$\Delta w_{sp}(\Delta t) = \begin{cases} \kappa^2 \mathrm{e}^{-\frac{\Delta t}{\tau}} \frac{\Delta t + \mathrm{e}^{2 + \frac{2\Delta t}{\tau}}\Delta t + 2\tau}{4\mathrm{e}^2\tau} & \text{if } -\tau < \Delta t \leq 0 \\ \kappa^2 \mathrm{e}^{-\frac{\Delta t}{\tau}} \frac{\Delta t + \mathrm{e}^2\Delta t + 2\tau}{4\mathrm{e}^2\tau} & \text{if } \Delta t > 0 \end{cases} \tag{36}$$

$$\Delta w_{sn}(\Delta t) = \begin{cases} -\kappa^2 \mathrm{e}^{\frac{\Delta t}{\tau}} \frac{\Delta t}{4\tau} & \text{if } \Delta t \leq -\tau \\ \kappa^2 \mathrm{e}^{-\frac{\Delta t}{\tau}} \frac{\Delta t + 2\tau}{4\mathrm{e}^2\tau} & \text{if } \Delta t > -\tau \end{cases} \tag{37}$$

$$\Delta w_{ps}(\Delta t) = \begin{cases} -\kappa^2 \mathrm{e}^{\frac{\Delta t}{\tau}} \frac{\mathrm{e}^2\Delta t + \Delta t - 2\tau}{4\mathrm{e}^2\tau} & \text{if } \Delta t \leq 0 \\ -\kappa^2 \mathrm{e}^{\frac{\Delta t}{\tau}} \frac{\mathrm{e}^2\Delta t + \mathrm{e}^{\frac{2\Delta t}{\tau}}(\Delta t - 2\tau)}{4\mathrm{e}^2\tau} & \text{if } 0 < \Delta t \leq \tau \end{cases} \tag{38}$$

$$\Delta w_{ns}(\Delta t) = \begin{cases} \kappa^2 \mathrm{e}^{\frac{\Delta t}{\tau}} \frac{2\tau - \Delta t}{4\mathrm{e}^2\tau} & \text{if } \Delta t \leq \tau \\ \kappa^2 \mathrm{e}^{-\frac{\Delta t}{\tau}} \frac{\Delta t}{4\tau} & \text{if } \Delta t > \tau \end{cases} \tag{39}$$

2.5 Maxima when $\tau_1 = \tau_2$

When $\tau_1 = \tau_2$ the formulas of the maximum values of the kernels, and the Δt causing them, are as follows (note how they are much simpler with respect to case of $\tau_1 \neq \tau_2$):

$$
\begin{aligned}
M_{p,p} &= \left(0, \kappa^2 \frac{\mathrm{e}^2 - 1}{4\mathrm{e}^2 \tau}\right) && (40)\\
M_{n,n} &= \left(0, \kappa^2 \frac{1}{4\mathrm{e}^2 \tau}\right) && (41)\\
M_{n,p} &= \left(\frac{2\mathrm{e}^2 \tau}{1+\mathrm{e}^2}, \kappa^2 \frac{\mathrm{e}^{-\frac{2\mathrm{e}^2}{1+\mathrm{e}^2}}(1+\mathrm{e}^2)}{4\mathrm{e}^2 \tau}\right) && (42)\\
M_{p,n} &= \left(\frac{2\mathrm{e}^2 \tau}{1+\mathrm{e}^2}, \kappa^2 \frac{\mathrm{e}^{-\frac{2\mathrm{e}^2}{1+\mathrm{e}^2}}(1+\mathrm{e}^2)}{4\mathrm{e}^2 \tau}\right) && (43)\\
M_{s,p} &= \left(\tau \frac{\mathrm{e}^2 - 1}{\mathrm{e}^2 + 1}, \frac{\kappa^2}{4} \mathrm{e}^{-\frac{1+3\mathrm{e}^2}{\mathrm{e}^2+1}}(1+\mathrm{e}^2)\right) && (44)\\
M_{p,s} &= \left(-\tau \frac{\mathrm{e}^2 - 1}{\mathrm{e}^2 + 1}, \frac{\kappa^2}{4} \mathrm{e}^{-\frac{1+3\mathrm{e}^2}{\mathrm{e}^2+1}}(1+\mathrm{e}^2)\right) && (45)\\
M_{s,n} &= \left(-\tau, \kappa^2 \frac{1}{4\mathrm{e}}\right) && (46)\\
M_{n,s} &= \left(\tau, \kappa^2 \frac{1}{4\mathrm{e}}\right) \ . && (47)
\end{aligned}
$$

2.6 The integrals of the synaptic changes over all Δt

The overall effect of each G-DHL rule component over the entire range of Δt can be computed with an integral computed over all values of component learning kernel. This information is relevant to characterise the total amount of enhancement or depression caused by each component when Δt is a stochastic variable with a flat distribution. This can be used to have a broad indication of the strength with which a G-DHL component, or a given combination of them, tend to cause a progressive drift of the synapse towards positive or negative values when Δt values vary, as it is normally the case in neural networks.

Interestingly, these integrals have a very simple form that depends only on κ for the differential components, and on κ and either τ_1 or τ_2 for the mixed components:

$$
\begin{aligned}
\int_{-\infty}^{\infty} \Delta w_{pp}(\Delta t)\,\mathrm{d}\,\Delta t &= \int_{-\infty}^{\infty} \Delta w_{nn}(\Delta t)\,\mathrm{d}\,\Delta t = \frac{\kappa^2}{\mathrm{e}^2} && (48)\\
\int_{-\infty}^{\infty} \Delta w_{np}(\Delta t)\,\mathrm{d}\,\Delta t &= \int_{-\infty}^{\infty} \Delta w_{pn}(\Delta t)\,\mathrm{d}\,\Delta t = \frac{\kappa^2}{\mathrm{e}^2} && (49)\\
\int_{-\infty}^{\infty} \Delta w_{sp}(\Delta t)\,\mathrm{d}\,\Delta t &= \int_{-\infty}^{\infty} \Delta w_{sn}(\Delta t)\,\mathrm{d}\,\Delta t = \frac{\kappa \tau_1}{\mathrm{e}} && (50)\\
\int_{-\infty}^{\infty} \Delta w_{ps}(\Delta t)\,\mathrm{d}\,\Delta t &= \int_{-\infty}^{\infty} \Delta w_{ns}(\Delta t)\,\mathrm{d}\,\Delta t = \frac{\kappa \tau_2}{\mathrm{e}} \ . && (51)
\end{aligned}
$$

2.7 Automatic search of the G-DHL rule components

The best G-DHL model to fit a target STDP data set can be found through an automatic procedure searching the best combination of the rule components, their

parameters σ and η, and the parameters κ, τ_1, and τ_2. We illustrate the approach used here to do this but other approaches are possible. To find the optimal combination of the G-DHL components for each target STDP data set we used a model comparison technique based on the *Bayesian Information Criterion* (BIC; [7]). This technique selects the best model based on a principled trade-off between (a) a low number of parameters used and (b) a low residual variance unexplained by the model after the fit. In particular, for each target STDP data set the automatic procedure runs all possible regressions of the target data set using a certain number c of components ($1 \leq c \leq 8$). Then for each combination of components, the regression procedure estimates: (a) the σs and ηs parameters of the selected components of the G-DHL rule, establishing the shape of the learning kernel; (b) the τ_1 and τ_1 time constants of the pre- and post-synaptic neurons establishing the horizontal size of the kernel; (c) the amplification parameter κ (Eq. (5)) establishing the vertical size of the kernel. Thus the number of the estimated parameters is $k = c + 3$, hence $4 \leq k \leq 11$.

Regarding the specific technique used to perform the regression, it is important to notice that although the G-DHL rule is linear in its components the needed regression is non-linear due to the interactions between the σs and ηs parameters, on one side, and the κ, τ_1, and τ_2 parameters, on the other. Any non-linear regression approach could be used to this purpose. Here we used a technique based on *genetic algorithms* [8] due to its simplicity, computational power, and availability of software (the genetic algorithm used here is explained in detail in Section 2.8).

For each data set, the best model (i.e., the best G-DHL rule combination of components and parameters) was considered the one minimising the BIC index:

$$BIC = n \cdot ln\left(\frac{1}{n}\sum_{i=1}^{n}\left(\Delta w_i(\Delta t) - \Delta w_i^p(\Delta t)\right)^2\right) + k \cdot ln\,(n) \tag{52}$$

where n is the number of data points, $ln(.)$ is the natural logarithm, $\Delta w_i(\Delta t)$ is the i experimental data point corresponding to the synaptic change measured at Δt, $\Delta w_i^p(\Delta t)$ is the corresponding synaptic change predicted by the model for Δt, and k is the number of estimated parameters of the model. Having the minimum BIC, the best found model has a low residual variance and at the same time a low number of free parameters.

Although the whole procedure involves a non-linear regression it tends to not fall into local minima, a desirable features typical of linear optimisation techniques. The reasons are that: (a) for each data set, the procedure runs a separated regression for each possible component combination, thus excluding the possibility of getting trapped into a particular combination as a local minimum; (b) the specific regression for each combination tends to not fall into local minima as the curves to be fit generally present few synapse strengthening/weakening peaks (one to three); local minima can also be easily excluded by direct inspection after getting the results of the search.

After finding the best model for each STDP data set, its capacity to accurately fit the data was measured as fraction of variance unexplained (FVU):

$$FVU = 1 - R^2 = 1 - \frac{\sum_i \left(\Delta w_i(\Delta t) - \Delta w_i^p(\Delta t)\right)^2}{\sum_i \left(\Delta w_i(\Delta t) - \Delta \bar{w}\right)^2}, \tag{53}$$

where R^2 is the *determination coefficient*, capturing the quality of the fit, and $\Delta \bar{w}$ is the mean of all data points.

2.8 Genetic algorithm to fit STDP data

To search for the parameters producing the best fit of each given data set we used a *genetic algorithm* optimisation technique [9, 10]. Genetic algorithms [8, 11–13] are a

popular optimisation methods and have been successfully used to estimate the parameters of non-linear models applied to a variety of biological data, including data on brain (e.g., [14–18]). The optimisation was here performed using the *Genetic Algorithm Toolbox* of *Matlab*^{TM}. The range of the parameters was normalised in $[0, 1]$. Each GA was run for 100 'generations' and individuals were reproduced with a 0.1 probability of crossover. The mutation was set to 'mutationadaptfeasible' (this chooses mutations that respect parameter boundaries) and the selection to 'selectionuniform'. The initial population of the GA used for one regression was chosen so that the individuals covered the space of the chosen parameters as much as possible in a uniform way.

The genetic algorithm was used in combination with a model comparison technique based on the *Bayesian Information Criterion* (BIC; [7]) described in Section 1.7. In particular, for each target STDP data set we considered all possible combinations of c $(1 \leq c \leq 8)$ G-DHL components having coefficients σ and η different from zero: for each combination, these parameters were then optimised with the genetic algorithm. To face the problem of possible local minima affecting non-linear optimisations, for each of such combinations we ran ten different independent regressions using different seeds of the random number generator, and chose the parameter set from the ten repetitions that produced the best fit in terms of minimum FVU (fraction of variance unexplained, see main article). We repeated this process for all combinations of parameters corresponding to a given c, and selected the best model among them. Finally, we selected the best model among those corresponding to different c on the basis of the BIC model comparison procedure. The overall number of GA regressions run for each experimental data set was therefore 2550 (see table 1).

Table 1. The number of regressions run for each data set organised for groups, where each group involved c components of the rule ($c = \{1, \ldots, 8\}$) combined in all possible ways. For each combination of components the regression was repeated 10 times.

Number of components:	1	2	3	4	5	6	7	8	
Number of combinations:	8	28	56	70	56	28	8	1	
Number of simulations:	80	280	560	700	560	280	80	10	**Tot: 2550**

3 Results: STDP regressions

The following figures show the details of the regressions reported in the main article for the different STDP data sets.

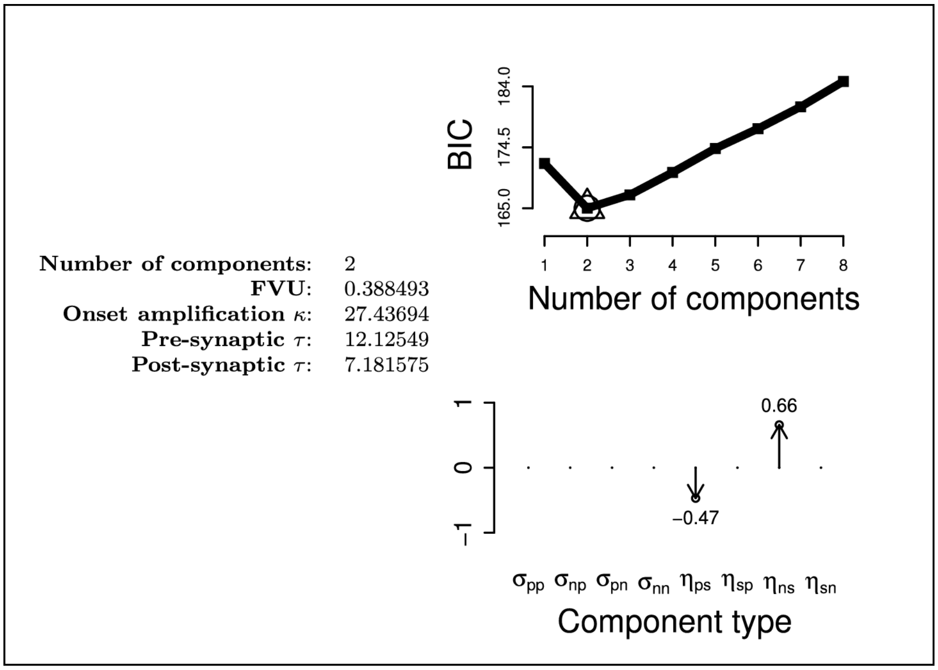

(a)

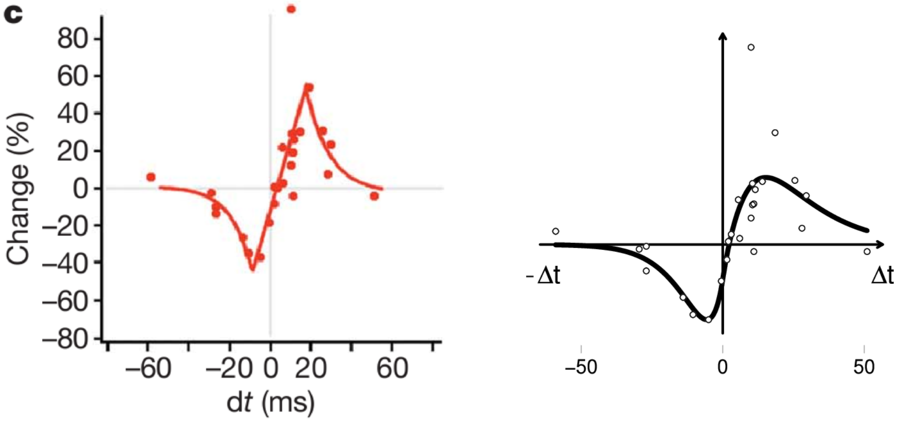

(b)

Figure 11. Results of the regression performed with the model selected with the BIC model comparison procedure applied to the STDP data from [19]. (a) Left column: number of components used by the best G-DHL-based regression; FVU (fraction of variance unexplained); values of the amplification parameter κ and of the pre/post-synaptic τs of the onset function (Eq. 13). Top-right graph: BIC values obtained using a number of G-DHL components from 1 to 8 and considering the best component combination for each number (the little triangle indicates the model used in the shown regression). Bottom-right graph: identity and size of the parameters found by the optimisation. (b) Left graph: data and exponential regression model from [19] (reprinted with permission). Right graph: fitting curve using the parameters reported in ‘a’.

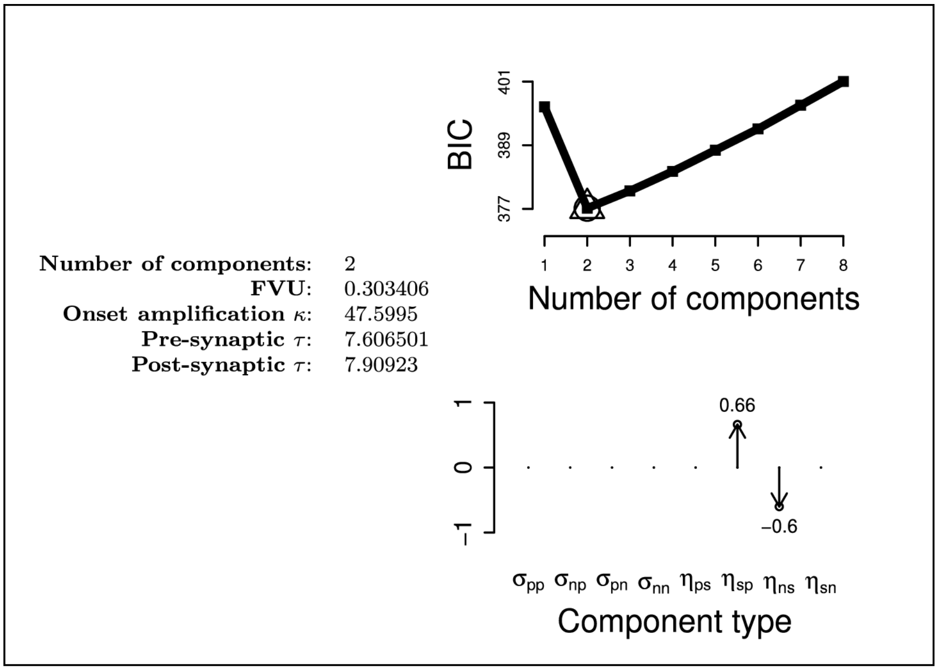

(a)

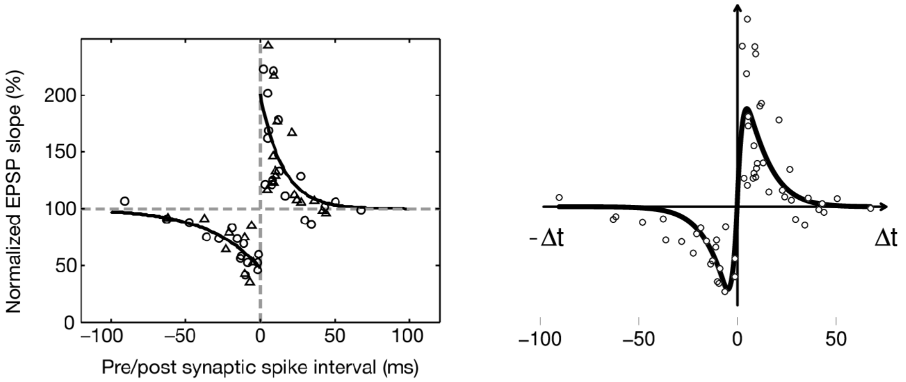

(b)

Figure 12. Results of the best G-DHL regression, selected with the BIC model comparison, applied to the STDP data from [20] (the left graph reported in 'b' is reprinted with permission). Data plotted as in Fig. 11.

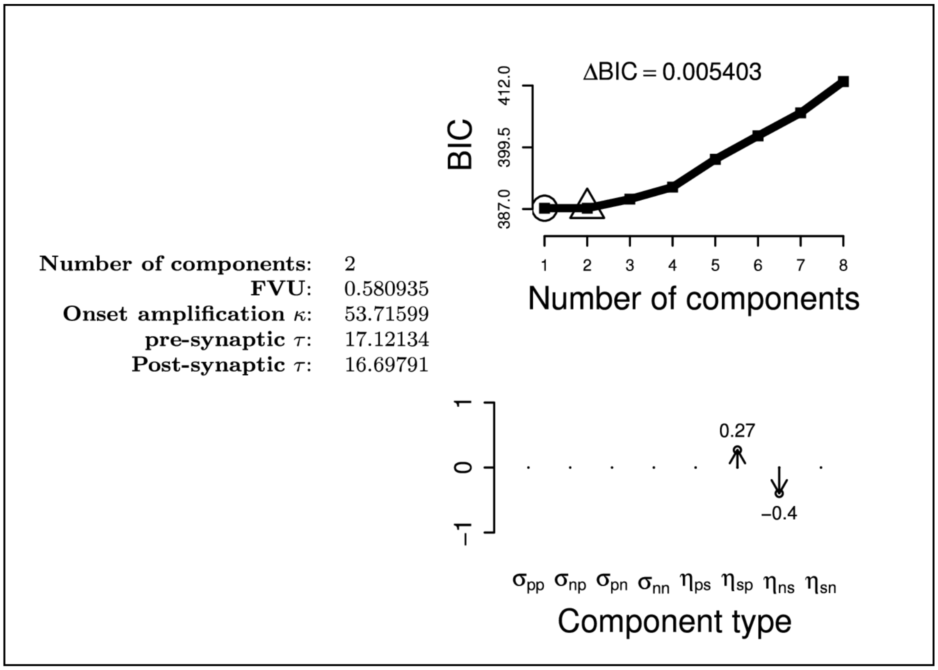

(a)

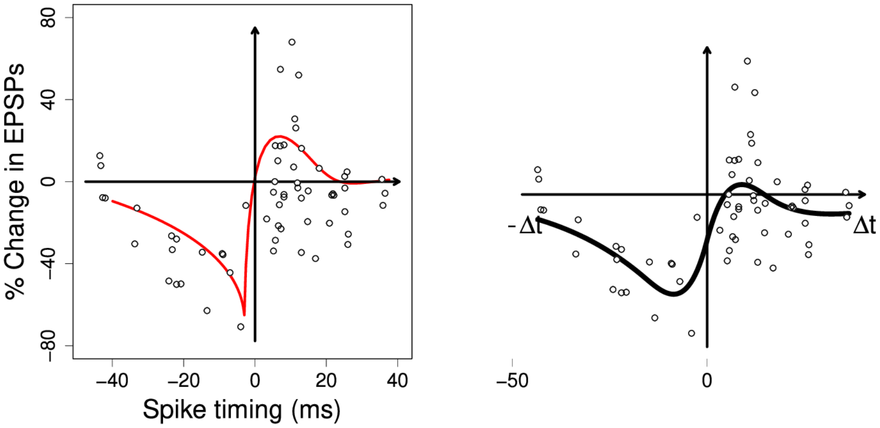

(b)

Figure 13. Results of the best G-DHL regression, selected with the BIC model comparison, applied to the STDP data from [21] (the left graph reported in 'b' was reproduced based on data and the original figure). Data plotted as in Fig. 11. Note that the graph at the top-right shows low BIC values for the regressions with 1 and 2 components: the regression with 1 component (little circle) has the minimum BIC value but the regression with 2 components (little triangle) was used as its BIC value is basically as the other but has a higher fitting power.

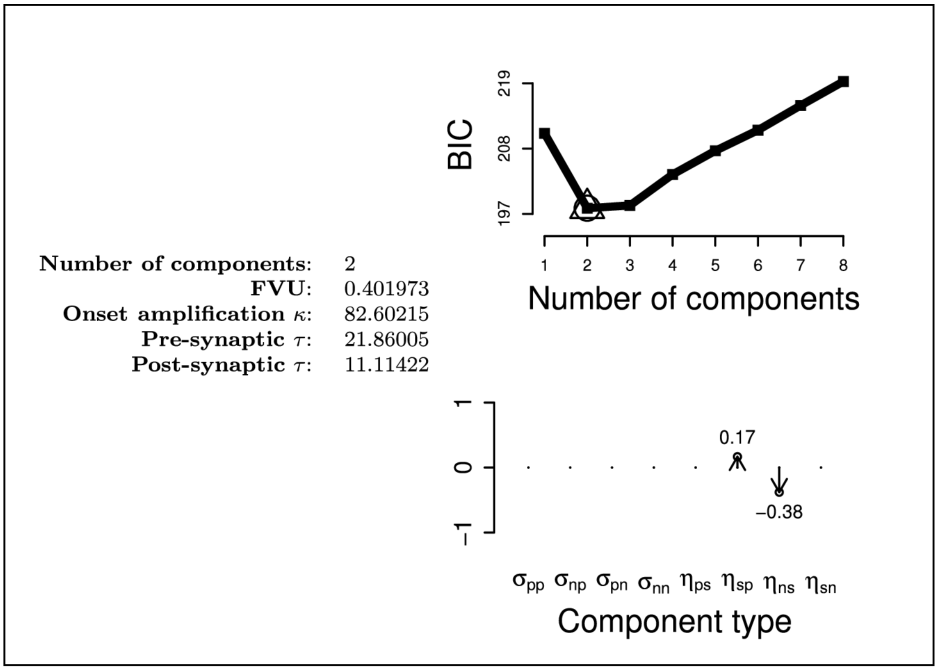

(a)

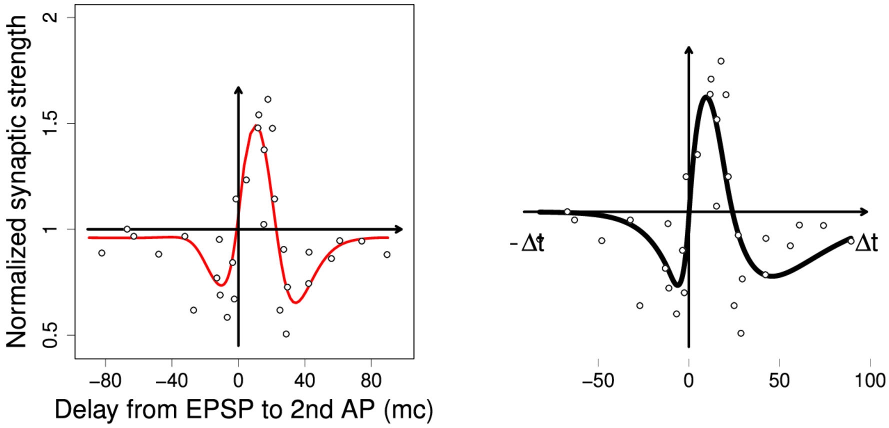

(b)

Figure 14. Results of the best G-DHL regression, selected with the BIC model comparison, applied to the STDP data from [22] (the left graph reported in 'b' was reproduced based on data and the original figure). Data plotted as in Fig. 11.

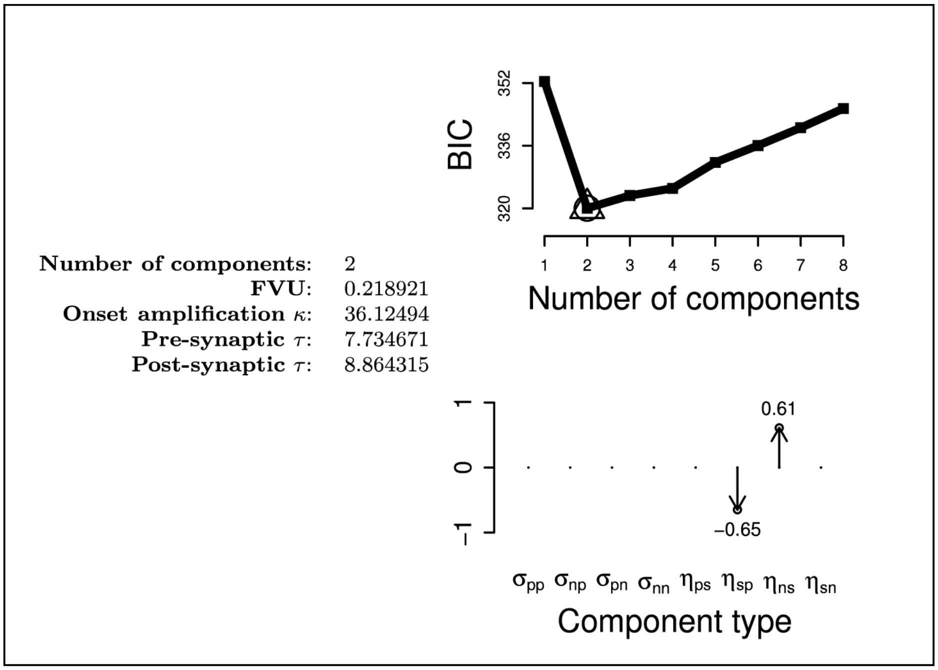

(a)

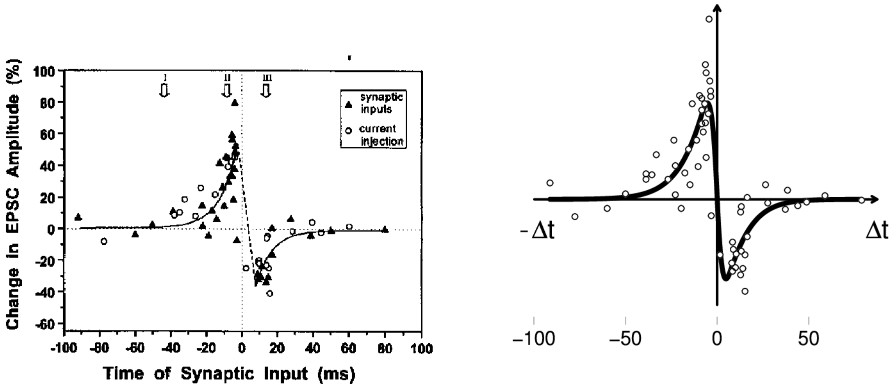

(b)

Figure 15. Results of the best G-DHL regression, selected with the BIC model comparison, applied to the STDP data from [23] (the left graph reported in 'b' is reprinted with permission). Data plotted as in Fig. 11.

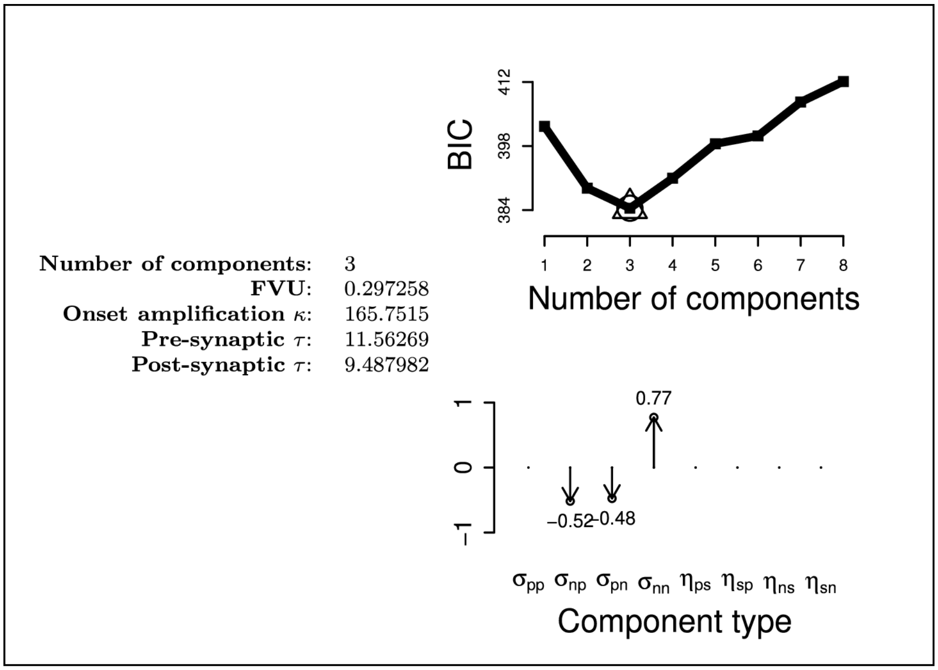

(a)

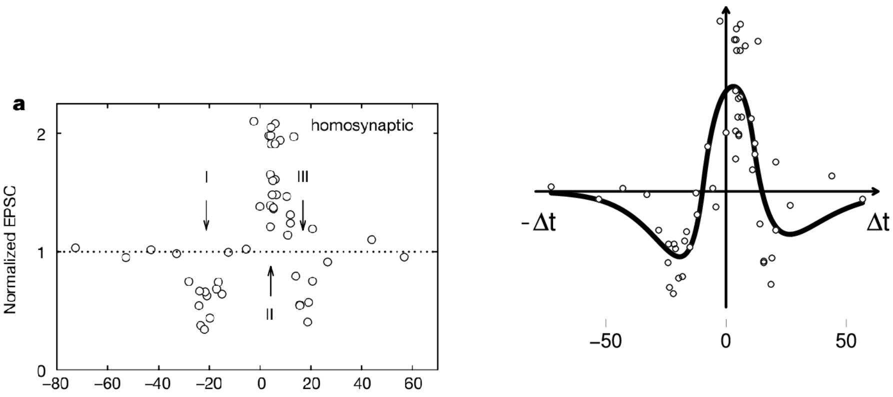

(b)

Figure 16. Results of the best G-DHL regression, selected with the BIC model comparison, applied to the STDP data from [24] (the left graph reported in 'b' is reprinted with permission). Data plotted as in Fig. 11.

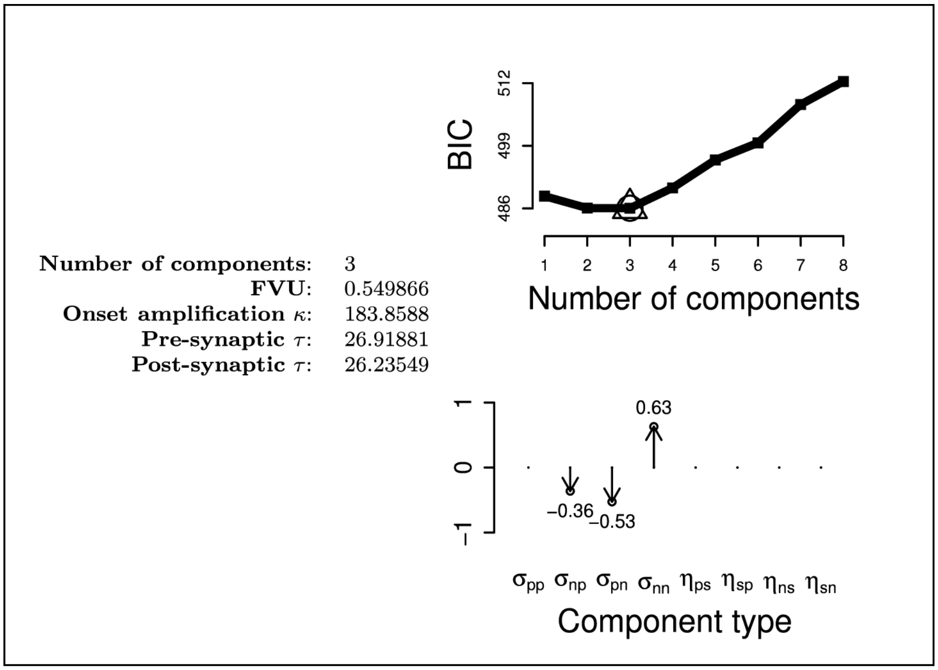

(a)

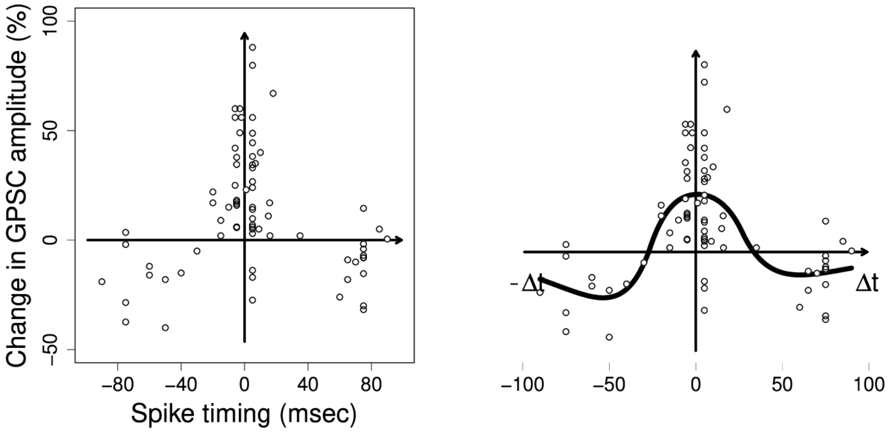

(b)

Figure 17. Results of the best G-DHL regression, selected with the BIC model comparison, applied to the STDP data from [25] (the left graph reported in 'b' was reproduced based on data). Data plotted as in Fig. 11.

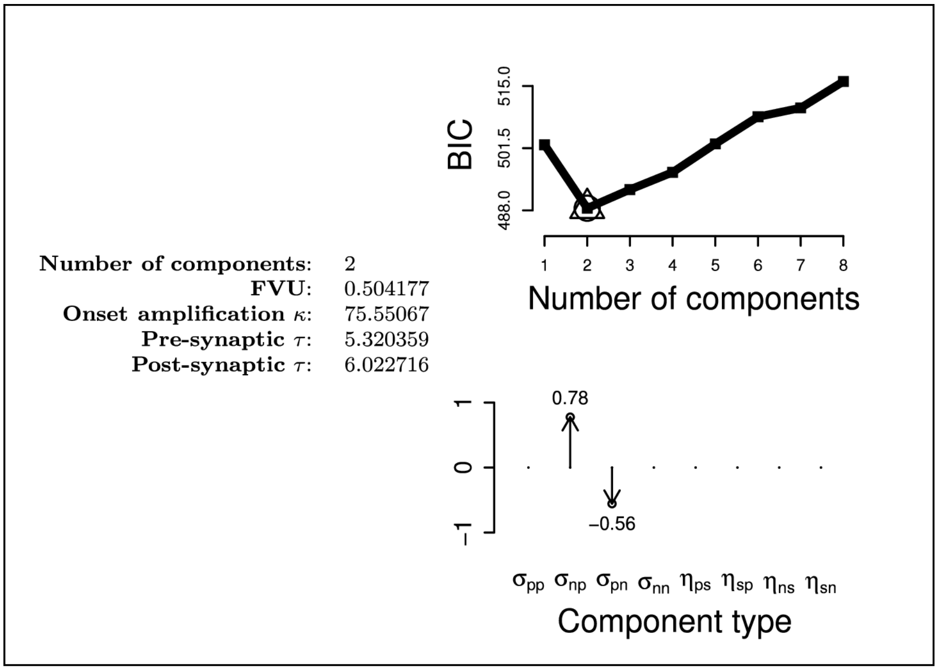

(a)

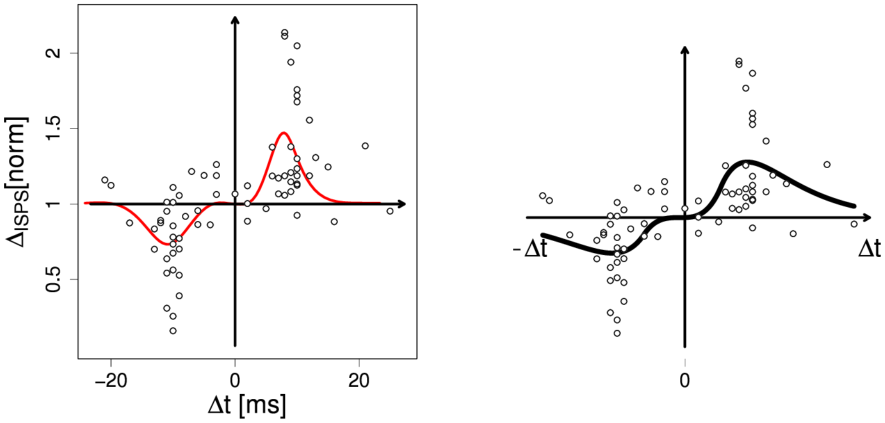

(b)

Figure 18. Results of the best G-DHL regression, selected with the BIC model comparison, applied to the STDP data from [26] (the left graph reported in 'b' was reproduced based on data and the original figure). Data plotted as in Fig. 11.

References

1. Kosko B. Differential Hebbian learning. AIP Conference Proceedings. 1986;151(1):277—282. doi:10.1063/1.36225.
2. Porr B, Wörgötter F. Isotropic sequence order learning. Neural Comput. 2003;15:831–864.
3. Gerstner W, Kistler WM. Spiking neuron models: single neurons, populations, plasticity. Cambridge: Cambridge University Press; 2002.
4. Dayan P, Abbott LF. Theoretical Neuroscience. Cambridge, MA: The MIT Press; 2001.
5. Oppenheim AV, Schafer RW. Discrete-time signal processing. Third edition. ed. Edinburgh, UK: Pearson; 2014.
6. Stuart GJ, Sakmann B. Active propagation of somatic action potentials into neocortical pyramidal cell dendrites. Nature. 1994;367(6458):69–72. doi:10.1038/367069a0.
7. Schwarz G. Estimating the dimension of a model. The annals of statistics. 1978;6(2):461–464.
8. Mitchell M. An introduction to genetic algorithms. Cambridge, MA: The MIT Press; 1996.
9. Yang XS. Metaheuristic optimization. Scholarpedia. 2011;6(8):11472.
10. Talbi EG. Metaheuristics: from design to implementation. vol. 74. Wiley; 2009.
11. Holland JH. Adaptation in natural and artificial systems: an introductory analysis with applications to biology, control, and artificial intelligence. Ann Arbor, MI: University of Michigan Press; 1975.
12. Goldberg DE. Genetic Algorithms in Search, Optimization, and Machine Learning. Addison-Wesley Professional; 1989.
13. Fogel DB. Evolutionary Computation: The Fossil Record. Wiley-IEEE Press; 1998.
14. VanderNoot TJ, Abrahams I. The use of genetic algorithms in the non-linear regression of immittance data. Journal of Electroanalytical Chemistry. 1998;448(1):17–23. doi:10.1016/S0022-0728(97)00593-7.
15. Gulsen M, Smith AE, Tate DM. A genetic algorithm approach to curve fitting. International Journal of Production Research. 1995;33(7):1911–1923. doi:10.1080/00207549508904789.
16. Yao L, Sethares WA. Nonlinear parameter estimation via the genetic algorithm. IEEE Transactions on Signal Processing. 1994;42(4):927–935. doi:10.1109/78.285655.
17. Kapanoglu M, Koc IO, Erdogmus S. Genetic algorithms in parameter estimation for nonlinear regression models: an experimental approach. Journal of Statistical Computation and Simulation. 2007;77(10):851–867. doi:10.1080/10629360600688244.

18. Viejo G, Khamassi M, Brovelli A, Girard B. Modelling choice and reaction time during arbitrary visuomotor learning through the coordination of adaptive working memory and reinforcement learning. Frontiers in Behvaioural Neuroscience. 2015;9(225):1–19. doi:10.3389/fnbeh.2015.00225.

19. Cassenaer S, Laurent G. Hebbian STDP in mushroom bodies facilitates the synchronous flow of olfactory information in locusts. Nature. 2007;448(7154):709–713. doi:10.1038/nature05973.

20. Froemke RC, Dan Y. Spike-timing-dependent synaptic modification induced by natural spike trains. Nature. 2002;416(6879):433–438. doi:10.1038/416433a.

21. Zhou YD, Acker CD, Netoff TI, Sen K, White JA. Increasing Ca2+ transients by broadening postsynaptic action potentials enhances timing-dependent synaptic depression. P Natl Acad Sci USA. 2005;102(52):19121–19125. doi:10.1073/pnas.0509856103.

22. Wittenberg GM, Wang SSH. Malleability of spike-timing-dependent plasticity at the CA3-CA1 synapse. J Neurosci. 2006;26(24):6610–6617. doi:10.1523/JNEUROSCI.5388-05.2006.

23. Zhang LI, Tao HW, Holt CE, Harris WA, Poo MM. A critical window for cooperation and competition among developing retinotectal synapses. Nature. 1998;395(6697):37–44. doi:10.1038/25665.

24. Nishiyama M, Hong K, Mikoshiba K, Poo MM, Kato K. Calcium stores regulate the polarity and input specificity of synaptic modification. Nature. 2000;408(6812):584–588. doi:10.1038/35046067.

25. Woodin MA, Ganguly K, Poo MM. Coincident pre- and postsynaptic activity modifies GABAergic synapses by postsynaptic changes in Cl- transporter activity. Neuron. 2003;39(5):807–820.

26. Haas JS, Nowotny T, Abarbanel HDI. Spike-timing-dependent plasticity of inhibitory synapses in the entorhinal cortex. J Neurophysiol. 2006;96(6):3305–3313. doi:10.1152/jn.00551.2006.
